# Classical and intermediate monocytes scavenge non-transferrin-bound iron and damaged erythrocytes

**DOI:** 10.1172/jci.insight.98867

**Published:** 2019-04-18

**Authors:** David Haschka, Verena Petzer, Florian Kocher, Christoph Tschurtschenthaler, Benedikt Schaefer, Markus Seifert, Sieghart Sopper, Thomas Sonnweber, Clemens Feistritzer, Tara L. Arvedson, Heinz Zoller, Reinhard Stauder, Igor Theurl, Guenter Weiss, Piotr Tymoszuk

**Affiliations:** 1 Department of Internal Medicine II,; 2 Department of Internal Medicine V, and; 3 Department of Internal Medicine I, Medical University of Innsbruck, Innsbruck, Austria.; 4 Department of Oncology, Amgen Inc., Thousand Oaks, California, USA.

**Keywords:** Immunology, Metabolism, Innate immunity, Monocytes

## Abstract

Myelomonocytic cells are critically involved in iron turnover as aged RBC recyclers. Human monocytes are divided in 3 subpopulations of classical, intermediate, and nonclassical cells, differing in inflammatory and migratory phenotype. Their functions in iron homeostasis are, however, unclear. Here, we asked whether the functional diversity of monocyte subsets translates into differences in handling physiological and pathological iron species. By microarray data analysis and flow cytometry we identified a set of iron-related genes and proteins upregulated in classical and, in part, intermediate monocytes. These included the iron exporter ferroportin (FPN1), ferritin, transferrin receptor, putative transporters of non-transferrin-bound iron (NTBI), and receptors for damaged erythrocytes. Consequently, classical monocytes displayed superior scavenging capabilities of potentially toxic NTBI, which were augmented by blocking iron export via hepcidin. The same subset and, to a lesser extent, the intermediate population, efficiently cleared damaged erythrocytes in vitro and mediated erythrophagocytosis in vivo in healthy volunteers and patients having received blood transfusions. To summarize, our data underline the physiologically important function of the classical and intermediate subset in clearing NTBI and damaged RBCs. As such, these cells may play a nonnegligible role in iron homeostasis and limit iron toxicity in iron overload conditions.

## Abstract 2

Human classical and intermediate monocytes mediate clearance of non-transferrin-bound iron and erythrophagocytosis.

## Graphical abstract

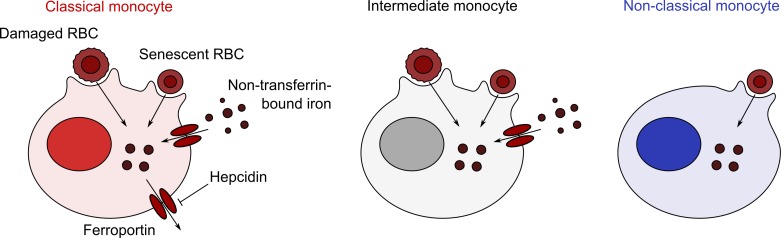

## Introduction

Iron is essential for numerous metabolic processes, such as oxygen transport, oxidative phosphorylation, and DNA synthesis (1). Tissue-resident macrophages orchestrate systemic iron flow by phagocytosing aged RBCs and delivering iron needed for erythropoiesis and metabolism via ferroportin (FPN1) (2–4). FPN1 is the sole known mammalian iron exporter, which is regulated at multiple levels by iron, cytokines, and the hormone hepcidin (1, 2, 5–8). Hepcidin binds to FPN1 and induces its internalization and degradation, leading to decreased iron efflux from macrophages and reduced dietary iron absorption (7, 9). Hepcidin expression is stimulated by high systemic iron levels (1, 4, 10) and diverse inflammatory cytokines (4, 6, 10). As such, hepcidin is responsible for the systemic iron sequestration during inflammation and infection (4, 6, 9–13). This mechanism is believed to have evolved as an antimicrobial defense measure and contributes to the so-called nutritional immunity (4).

Physiologically, the majority of extracellular iron is tightly bound by the transport protein transferrin (TF) — constituting so-called TF-bound iron (TBI) (1). The TF receptor 1 (TfR1) mediates uptake of iron-loaded holo-TF by the cell where surplus iron is stored in a chemically stable ferritin complex (1). Under pathological conditions, such as hemochromatosis, hemolysis, or transfusional stress, iron availability and damaged erythrocyte numbers can exceed the storage and recycling capabilities of the organism. This leads to expansion of loosely protein-bound, redox-active iron in the cell cytoplasm, so-called labile iron pool (LIP) (14), as well as accumulation of non-transferrin-bound iron (NTBI) and free heme in the serum (2, 15–17). Such chemically reactive iron forms cause cell and organ damage by catalyzing formation of toxic radicals (2, 15, 16) and can be directly utilized by some microbial pathogens (11, 12, 18, 19).

Monocytes are blood leukocytes that play important roles in innate immunity and development (20). Human monocytes can be divided into 3 subgroups: classical (CD14^+^CD16^–^), intermediate (CD14^+^CD16^+^), and nonclassical (CD14^–^ CD16^+^) monocytes (21), each displaying functional differences, e.g., in inflammatory (22, 23), migratory, and phagocytic capabilities (22). For decades monocytes have been thought to contribute to the systemic iron turnover as direct progenitors of macrophages (24). Recent research suggests that monocytes, with few exceptions, contribute to the macrophage pool only during inflammation and injury (25). However, monocytes are equipped with erythrocyte-scavenging receptors and have been shown to phagocytose damaged RBCs (26–30), pointing toward their stand-alone role in iron homeostasis.

In this study, we found that the classical and intermediate monocyte subpopulations expressed diverse iron turnover proteins and were able to efficiently scavenge NTBI and deposit it in ferritin-bound form. This later process was further bolstered when iron export was blocked by hepcidin. Mechanistically, the NTBI uptake in classical cells involved most likely both reductive and nonreductive pathways. Importantly, classical monocyte FPN1 levels and LIP were substantially regulated by iron availability in individuals suffering from genetic and transfusional iron overload. Finally, classical and intermediate monocytes proved to efficiently phagocytose erythrocytes in steady state and, especially, under transfusional stress. Therefore, classical and intermediate monocytes may play a previously unrecognized role in orchestrating local and systemic iron turnover and in protection from toxicity and infection risk caused by excessive iron accumulation.

## Results

### Human monocyte subsets differentially express iron metabolism genes.

**Figure 1:**
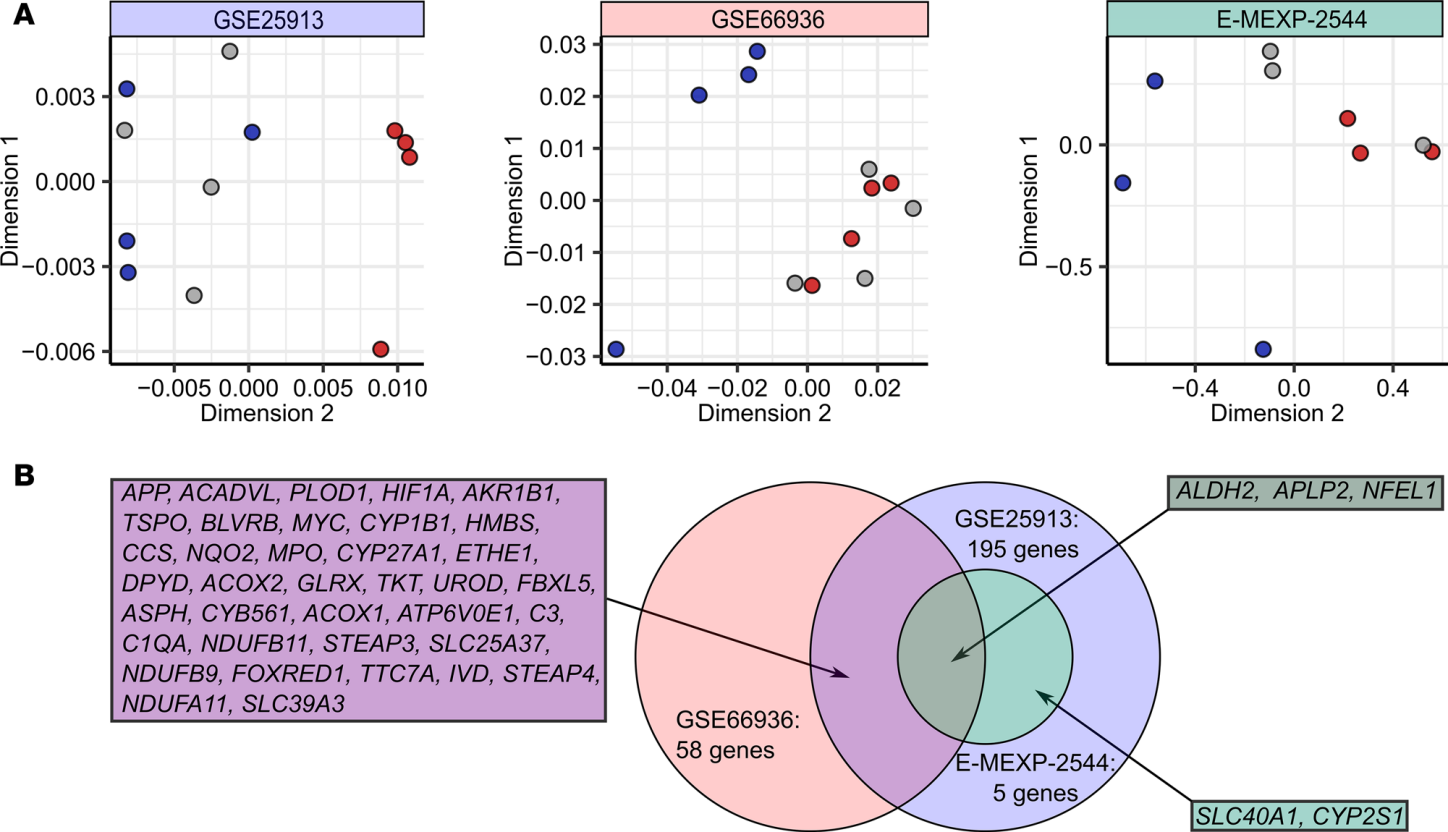
Differential expression of iron-related genes in human monocyte subsets. Monocyte subset-specific differences in expression of iron metabolism–related genes were analyzed in 3 whole-genome microarray studies using ANOVA, as described in Methods, Supplemental Figure 1A, and Supplemental Table 1 (GSE25913: *n* = 4, GSE66936: *n* = 4, and E-MEXP-2544: *n* = 3 healthy monocyte donors). Genes proving significant in at least 2 studies were further investigated. (**A**) Relationship among monocyte populations in terms of iron-related gene expression was visualized with principal coordinate analysis. Expression values for the entire iron-related gene set in each study were transformed to *Z*-scores and used to calculate Pearson’s distance between human monocyte samples in a 2-dimensional space. Each point represents 1 sample. Red: classical monocytes; gray: intermediate monocytes; blue: nonclassical monocytes. (**B**) Venn diagram showing significantly regulated iron-related genes.

To gain insight into potential differences in the iron-handling phenotype among human blood monocyte subpopulations, we investigated expression of over 500 iron-related genes in monocyte subpopulations in 3 published whole-genome expression studies (Supplemental Tables 1 and 2 and Supplemental Figure 1A; supplemental material available online with this article; https://doi.org/10.1172/jci.insight.98867DS1). In a 2-dimensional principal coordinate analysis, the classical monocyte and nonclassical monocyte samples formed separate clusters due to profound differences in the repertoire of iron-linked transcripts. The intermediate monocyte samples were found to group either with classical (2 studies) or nonclassical monocyte samples (1 study) (Figure 1A). This suggests, apart from the study-inherent differences in definition of the intermediate subpopulation, an iron metabolism phenotype displaying similarities with both remaining subsets.

**Figure 2:**
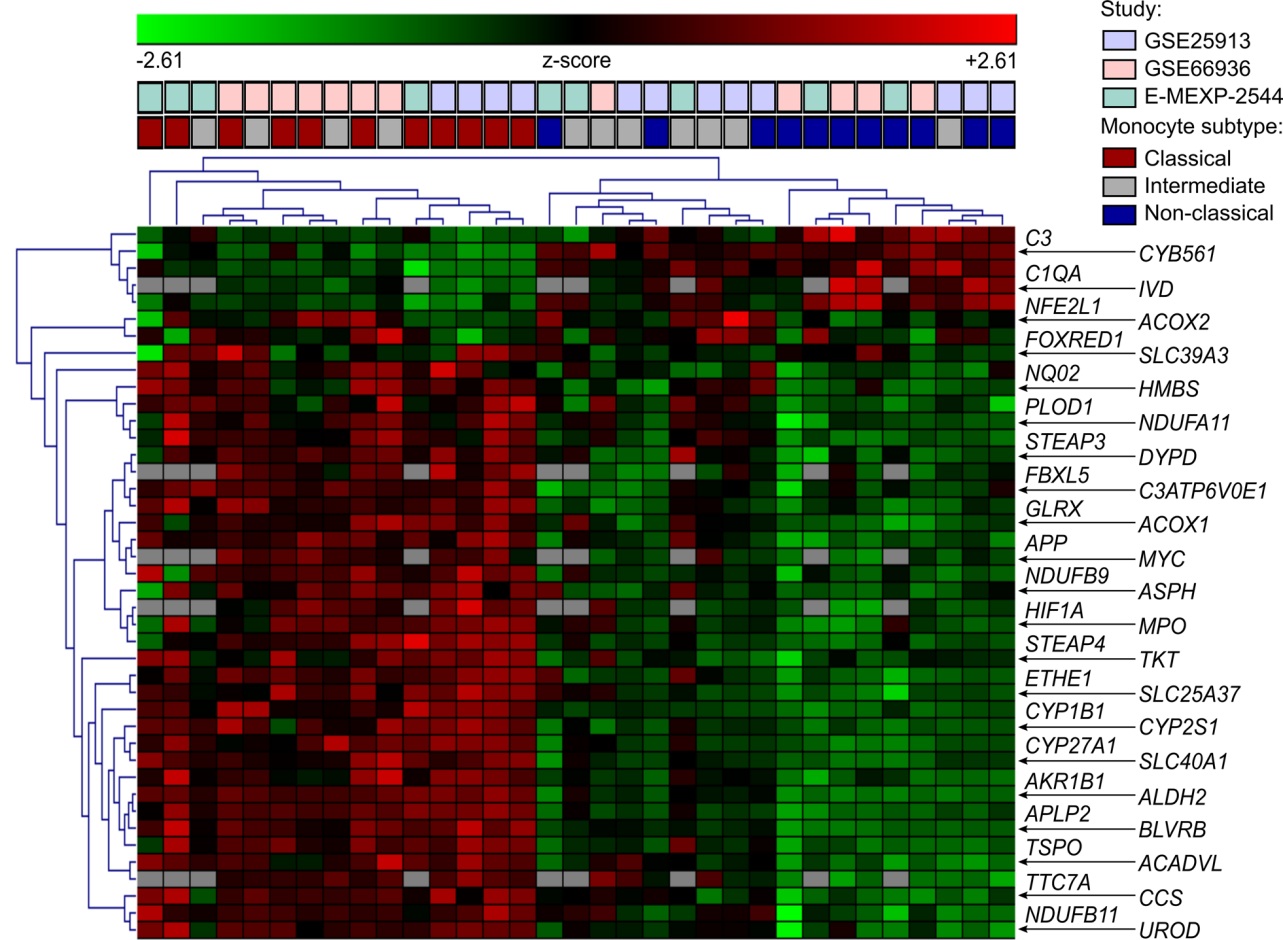
Preferential expression of iron-related genes in classical monocytes. Differentially regulated iron-related genes in 3 microarray studies (GSE25913: *n* = 4, GSE66936: *n* = 4, and E-MEXP-2544: *n* = 3 healthy monocyte donors) were identified by 1-way ANOVA as presented in Supplemental Figure 1A and Supplemental Table 2. Gene expression values in each study were transformed to *Z*-scores and presented as a gene- and sample-clustered heatmap (average linkage, euclidean distance). Color bars above the heatmap code for the study and monocyte subset.

Depending on the study, we detected between 5 and 195 iron-related genes, with expression that significantly differed between monocyte subtypes; those identified in at least 2 studies were further investigated (42 genes, Figure 1B). Expression of most of them peaked in the classical subset (35 genes, Figure 2). This classical monocyte-specific gene group encoded, among others, for proteins involved in iron import and intracellular trafficking (e.g., *FBXL5*, *SLC25A37*, *STEAP3*, *STEAP4*), iron export (*SLC40A1* coding for the sole iron exporter FPN1), energy and steroid metabolism enzymes utilizing iron as cofactor (e.g., *NDUFA11*, *NDUFB9*, *NQO1*, *CYP1B1*), and hypoxia and inflammatory response proteins (e.g., *HIF1A* and *MPO*). Only a few transcripts peaked in the intermediate (2 genes) and nonclassical subpopulation (5 genes) (Figure 2).

To identify shared iron turnover pathways and functional similarities in all 3 monocyte subsets, we searched for commonly expressed iron-linked genes in each monocyte subset. To this end, we identified 100 iron-linked transcripts demonstrating the lowest expression variability between monocyte subsets in each study; again, those identified in at least 2 of the studies were further investigated (Supplemental Figure 1B). This procedure yielded 54 transcripts (Supplemental Figure 1C). Diverse cytochrome P mRNAs constituted the majority of them (14 genes), along with iron-dependent oxygenases involved in detoxification (*DAO* and *MAO*), radical neutralizing enzymes (*PXDNL*), and fatty acid and heme metabolism genes (*ALAS2*, *ALOXE3*, *ACADL*, *ACAD10*, *BBOX1*, *AKR1C4*), which may point to activity of detoxification, biosynthesis and lipid β-oxidation pathways in all monocyte subpopulations. Genes involved in antimicrobial defense were overrepresented in the common gene set as well. These included heme-containing enzymes for reactive oxygen and nitrogen species synthesis (*NOS1*, *DUOX1*, *NOX5*, *CYBB*), iron chelator–binding protein (*LCN2*), and heme scavenger (*HPX*), fitting well to the primary antimicrobial function of pan-monocytes. We found also a group of hemoglobin transcripts in the common gene set — their presence might result from unspecific erythrocyte impurities in monocyte samples (31) or from more specific monocyte-erythrocyte association and RBC phagocytosis.

**Figure 3:**
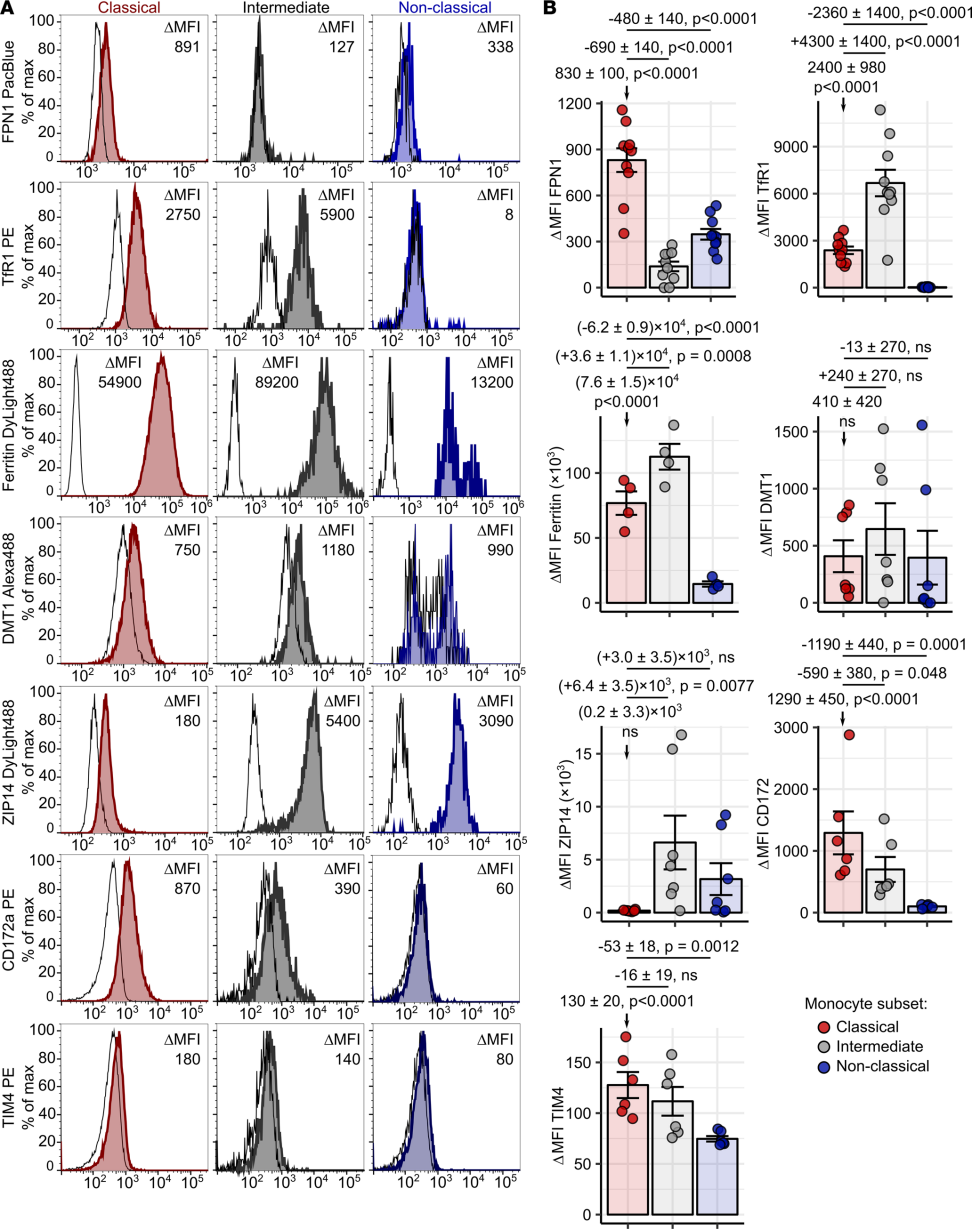
Surface expression of iron turnover proteins in monocyte subsets. Whole blood samples and PBMCs were obtained from healthy individuals. Expression of surface FPN1 (*n* = 10), TfR1 (*n* = 10), DMT1 (*n* = 7), ZIP14 (*n* = 7), CD172a (*n* = 6), TIM4 (*n* = 6), and intracellular ferritin (*n* = 4) in monocyte subsets was measured by flow cytometry. Blood monocyte subpopulations were defined as presented in Supplemental Figure 2A (red: classical; gray: intermediate; blue: nonclassical monocytes). (**A**) Representative signal histograms (open histograms: isotype; tinted histograms: specific antibody). (**B**) In graphs with ΔMFI values, each point represents 1 measurement, bars denote mean, and error bars represent SEM. Statistical significance was assessed with first-order linear models. Estimates for protein expression in classical monocytes and for differences in expression between subsets are presented with 95% CI. Estimate *P* values were calculated with 2-tailed *t* test. ANOVA statistics for each marker are shown in Supplemental Table 3.

To corroborate the results of the microarray analysis, we delved into surface and intracellular levels of iron turnover proteins in monocyte subtypes by flow cytometry (Supplemental Figure 2). In particular, we measured protein expression of FPN1, ferritin, the TBI importer TfR1, and candidate NTBI importers divalent metal transporter 1 (DMT1) (32) and Zrt- and Irt-like protein 14 (ZIP14) (33) as well as receptors for senescent and damaged erythrocytes signal regulatory protein 1 α (CD172a) (34) and T cell membrane protein 4 (TIM4) (35) (Figure 3 and Supplemental Figure 3). In line with the gene expression data (Figure 2), the classical subset expressed by far the highest levels of surface FPN1 protein among monocyte subpopulations. Classical and intermediate cells abundantly expressed ferritin, TfR1, and CD172a and showed detectable amounts of both investigated NTBI transporters. The nonclassical monocyte subtype, in turn, proved clearly positive for ferritin and ZIP14 (Figure 3).

Importantly, these results could be verified using another flow cytometry strategy defining pan-monocytes by HLA-DR positivity (Supplemental Figure 2B and Supplemental Figure 3). Taken together, the superior expression of diverse iron turnover transcripts and proteins in classical and intermediate monocytes may render those 2 subpopulations capable of uptake of various iron forms, efficient intracellular storage, and export of iron.

### Surface FPN1 protein on classical monocytes is regulated by hepcidin and iron.

**Figure 4:**
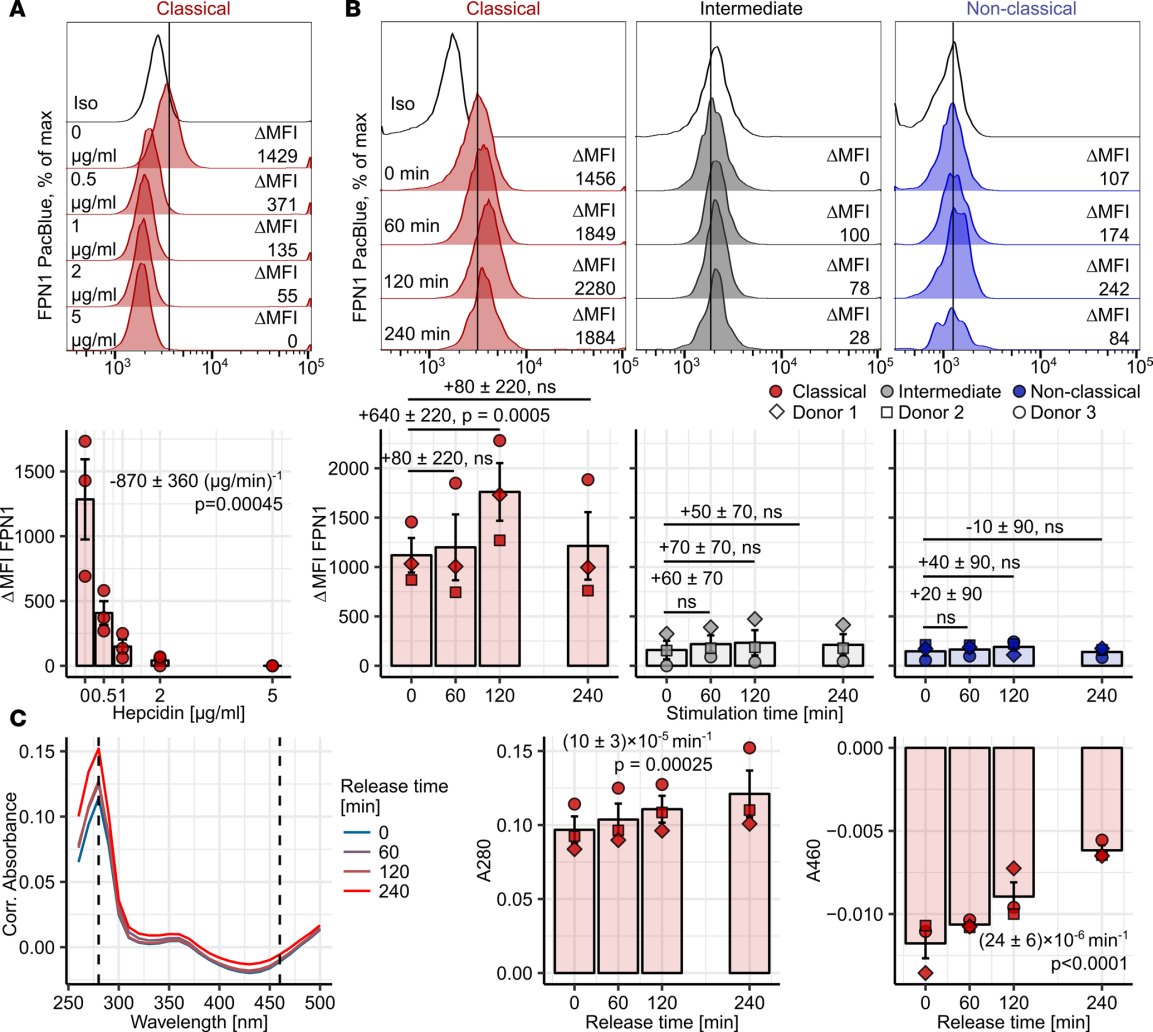
Regulation of classical monocyte FPN1 by hepcidin and iron and its functionality. (**A** and **B**) PBMCs (*n* = 3 healthy donors) were incubated with the indicated hepcidin concentrations (**A**) or 10 μM Fe^3+^ [**B**, Fe_2_(SO_4_)_3_]. Surface FPN1 levels in monocyte subsets were determined by flow cytometry. Monocyte subpopulations were defined as described in Supplemental Figure 2A (red: classical; gray: intermediate; blue: nonclassical monocytes). Representative signal histograms are shown (open histograms: isotype; tinted histograms: FPN1). Graphs display ΔMFI values; each point represents 1 measurement, bars denote mean, and error bars represent SEM. The cell donor is represented by symbol shape. Statistical significance was assessed with second-order (**A**) and first-order linear models (**B**); a separate model was applied to each monocyte subset. Estimates for the first-order hepcidin term (**A**) and for changes in FPN1 ΔMFI at particular time points (**B**) are shown with 95% CI. Estimate *P* values were calculated with 2-tailed *t* test. ANOVA for the first-order hepcidin term: *P* = 0.00023 (F_1,12_ = 27). ANOVA for the iron terms (**B**): classical monocytes: *P* = 0.00039 (F_3,9_ = 18); intermediate monocytes: *P* = 0.045 (F_3,9_ = 4); nonclassical monocytes: *P* = NS (F_3,9_ = 0.64). (**C**) 10 μM Fe^3+^-loaded CD14^+^ monocytes (*n* = 3 healthy donors) were incubated with 0.5 mg/ml apo-TF for the indicated time points. Apo-TF: holo-TF conversion in culture supernatant was monitored by absorbance measurements at 280 (A280) and 460 nm (A460). Culture medium without apo-TF served as a blank sample. Representative absorbance spectra are shown. Graphs depict absorbance values. Each point represents 1 measurement, bars denote mean, and error bars represent SEM. The cell donor is represented by symbol shape. Statistical significance was assessed with second-term linear models. Estimates for absorbance change rate are shown with 95% CI. Estimate *P* values were calculated with 2-tailed *t* test. ANOVA for the rate term: A_280_: *P* = 0.00013 (F_1,9_ = 40); A_460_: *P* < 0.0001 (F_1,9_ = 63).

Binding of hepcidin to FPN1 causes its internalization and blocks iron efflux (7). Surface FPN1 in classical monocytes was significantly downregulated by hepcidin in a concentration-dependent manner (Figure 4A). Intracellular iron concentration is another factor influencing FPN1 expression (3, 8). Challenge of monocytes with 10 μM Fe^3+^ [as Fe_2_(SO_4_)_3_] led to a strong transient upregulation of surface FPN1 levels specifically in the classical subset (Figure 4B).

Next, we sought to verify the functionality of FPN1 in classical monocytes. In the case when iron-loaded FPN1-expressing cells actively export iron, a conversion of apo-TF to holo-TF in the culture media should take place. This reaction can be tracked by measuring changes in absorbance at 280 and 480 nm in the TF-containing culture supernatant (Supplemental Figure 4, B and C) (36). MACS-purified CD14^+^ cells, consisting of classical and intermediate cells (Supplemental Figure 4A), which had been previously incubated in iron-rich medium, induced a clearly detectable conversion of apo-TF to holo-TF, demonstrating the export activity of monocytic FPN1 (Figure 4C).

### NTBI is preferentially taken up by classical and intermediate monocytes.

Efficient NTBI removal is crucial for avoiding radical-mediated cell damage and constitutes an important line of antimicrobial defense (15, 19). Recent reports highlight the contribution of hepcidin to this process, but a hepcidin effector cell population has not been defined so far (11, 12). As demonstrated above, classical monocytes express fully functional and hepcidin-sensitive FPN1 together with two putative NTBI importers, DMT1 and ZIP14, on their surface (Figures 3 and 4 and Supplemental Figure 3). We questioned whether those fairly abundant leukocytes could react to hepcidin by reducing export and increasing iron deposition in the cell and, hence, contribute to the systemic NTBI-scavenging properties of hepcidin.

**Figure 5:**
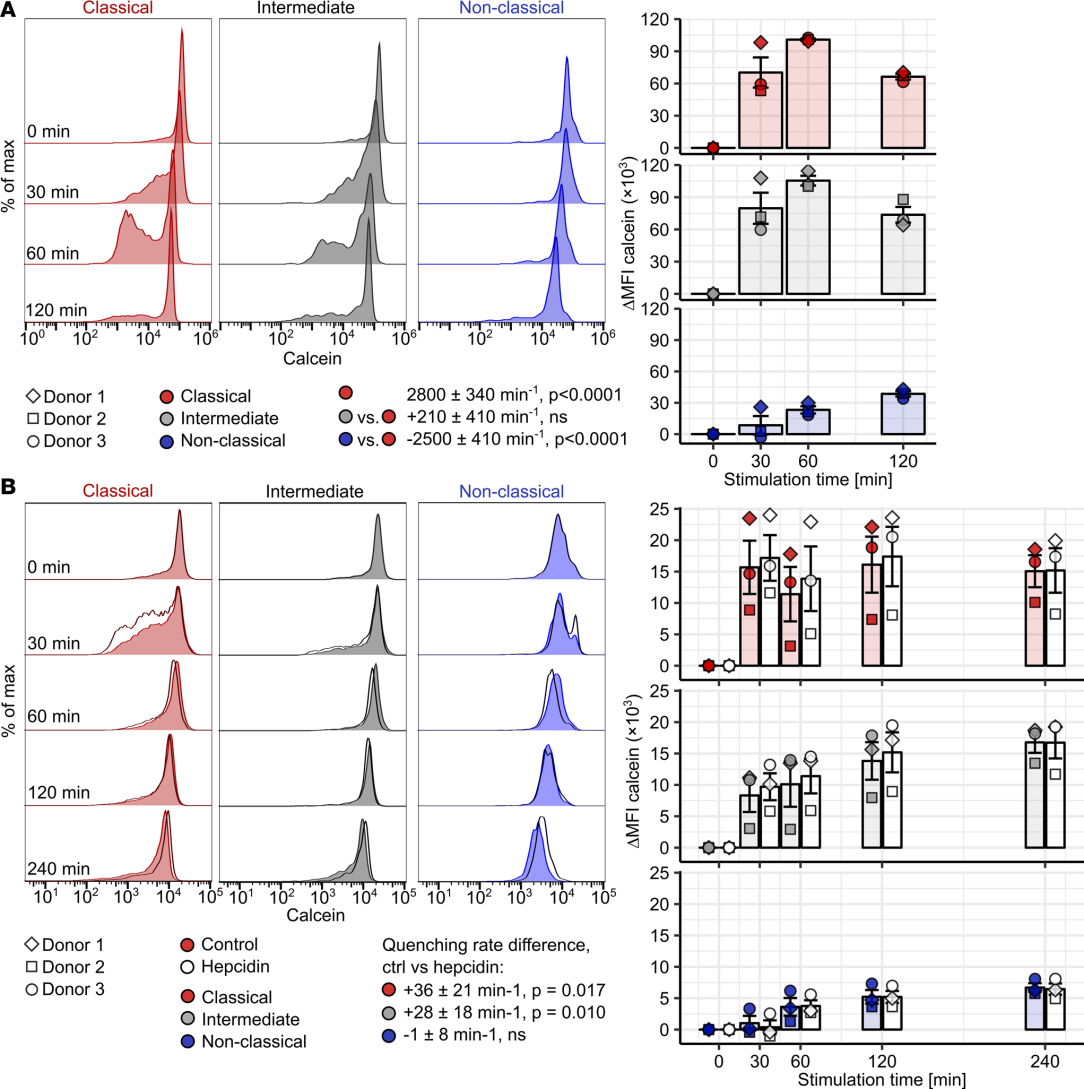
Hepcidin-enhanced LIP increase in NTBI-challenged classical and intermediate monocytes. (**A** and **B**) Calcein-labeled PBMCs (*n* = 3 healthy donors) were cultured with (**A**) 10 μM Fe^3+^ or (**B**) 10 μM Fe^3+^ in form of Fe_2_(SO_4_)_3_ with/without 2 μg/ml hepcidin for the indicated time points. Calcein fluorescence in monocyte subpopulations was measured by flow cytometry. Monocyte subpopulations were defined as described in Supplemental Figure 2A (red: classical; gray: intermediate; blue: nonclassical monocytes). Representative calcein histograms are shown (tinted histograms: control; open histograms: hepcidin). Graphs show ΔMFI values (colored symbols: control; open symbols: hepcidin). Each point represents 1 measurement, bars denote mean, and error bars represent SEM. The cell donor is represented by symbol shape. Rate of calcein quenching was determined with second-order linear models. In **B**, a separate model was applied to each monocyte subset. All estimates are shown with 95% CI. Estimate *P* values were calculated with 2-tailed *t* test. (**A**) Estimates for quenching rate in classical monocytes and differences in quenching rate between particular monocyte subsets. (**B**) Estimates for differences in quenching rate between control and hepcidin-stimulated cells. ANOVA statistics are presented in Supplemental Table 4.

Iron, after entering the cell, forms cytoplasmic LIP, the amount of which can be gauged by the iron-quenched fluorophore calcein (14, 37, 38). Human monocytes challenged with NTBI [10 μM Fe^3+^ as Fe_2_(SO_4_)_3_, Supplemental Figure 4D] expanded LIP with a rapid, saturable kinetics (Figure 5A). The LIP accumulation rate peaked in classical and intermediate monocytes and drastically surmounted the LIP expansion kinetics of nonclassical monocytes (Figure 5A). In a direct comparison among major blood leukocyte populations, classical monocytes demonstrated the most rapid NTBI-induced LIP accumulation, followed by neutrophils and B cells (Supplemental Figure 5). Importantly, LIP expansion rates in CD4^+^ and CD8^+^ T cells described before as NTBI scavengers (39, 40) were found to be significantly lower than in classical monocytes.

Subsequently, we asked which of the 2 species, TBI or NTBI, is preferentially taken up by monocytes. Monocytes exposed to fluorescently labeled holo-TF endocytosed it, clearly indicating TBI import abilities. TBI import rates were, as with NTBI, the highest in classical and intermediate cells (Supplemental Figure 6). However, when we exposed classical monocytes to NTBI and TBI with equimolar Fe^3+^ amounts, NTBI increased LIP significantly faster than TBI (Supplemental Figure 7A). Analogically, NTBI bolstered surface FPN1 with a faster kinetics than TBI in those cells (Supplemental Figure 7B). To verify, that classical monocytes remove NTBI more efficiently from their surrounding than TBI, we incubated CD14^+^ monocytes with TBI and NTBI containing identical amounts of radioactive Fe^3+^. In this setting, NTBI uptake clearly exceeded TBI absorption in a 4-hour assay time frame (Supplemental Figure 7C).

**Figure 6:**
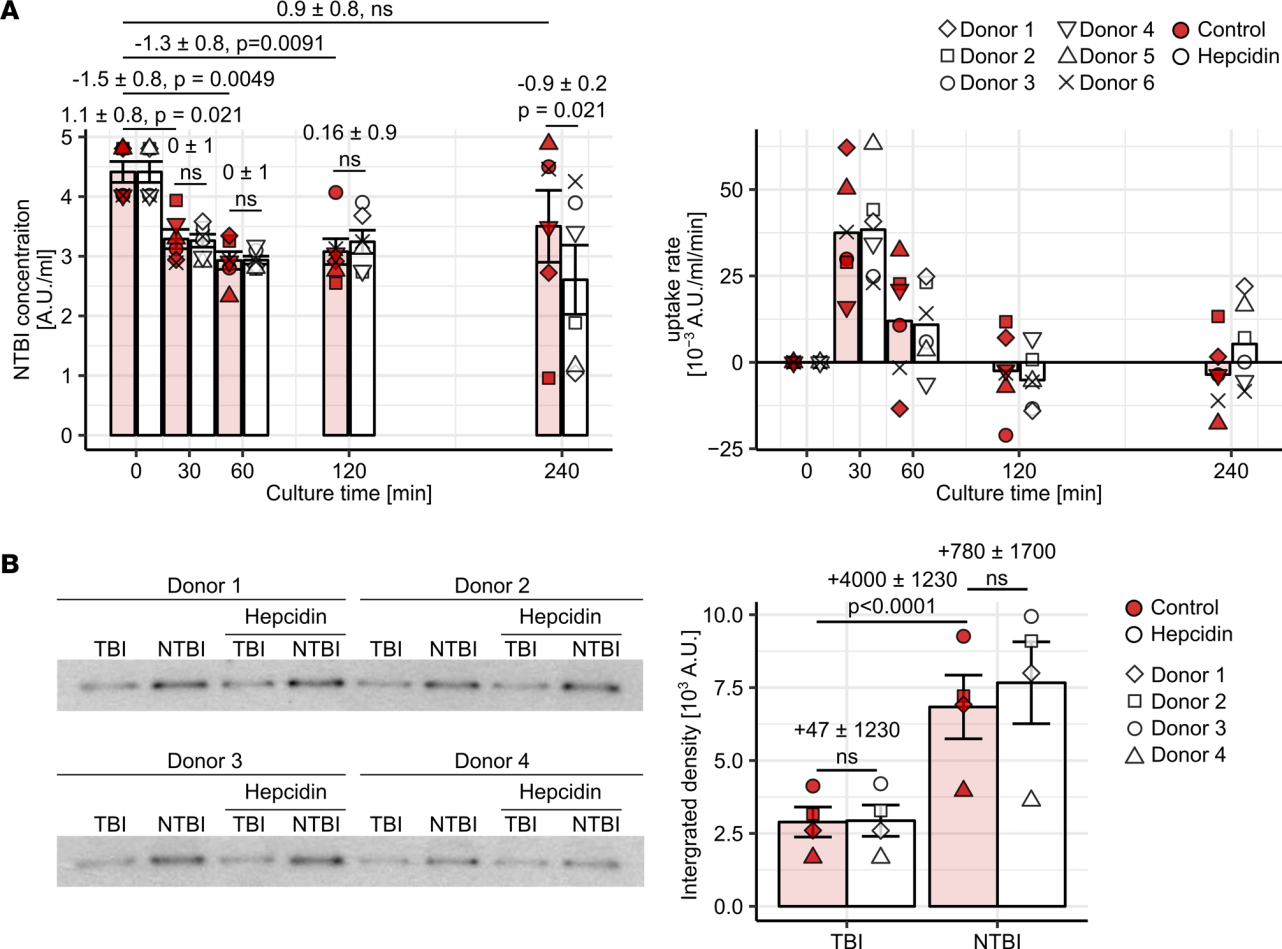
Hepcidin-enhanced NTBI clearance by classical and intermediate monocytes. (**A**) CD14^+^ monocytes (*n* = 6 healthy donors) were cultured with 10 μM Fe^3+^ [Fe_2_(SO_4_)_3_] with/without 2 μg/ml hepcidin. NTBI concentration in supernatant and NTBI uptake rate at the indicated time points were determined. On the graphs, each point represents 1 measurement, bars denote mean, and error bars represent SEM. The cell donor is represented by symbol shape. Statistical significance was assessed with a first-order linear model. Estimates for the baseline NTBI concentration, changes in concentration at the particular time points, and for differences in concentration between control and hepcidin-stimulated samples are shown with 95% CI. Estimate *P* values were calculated with 2-tailed *t* test. ANOVA for the time and time-hepcidin interaction terms: *P*_time_ = 0.00057 (F_5,30_ = 6.7), *P*_time-hepcidin_ = NS (0.13) (F_5,30_ = 1.9). (**B**) CD14^+^ monocytes (*n* = 4 healthy donors) were stimulated with 0.5 μM ^59^Fe^3+^ (TBI) or 0.5 μM ^59^Fe^3+^ and 10 μM ^56^Fe^3+^ (NTBI) with/without 2 μg/ml hepcidin for 4 hours. ^59^Fe-ferritin in cell lysates was visualized by autoradiography. Autoradiograms and densitometry results are shown (colored symbols: control, open symbols: hepcidin). On the graphs, each point represents 1 measurement, bars denote mean, and error bars represent SEM. The cell donor is represented by symbol shape. Statistical significance was estimated with a first-order linear model. Estimates for changes in ^59^Fe-ferritin levels between culture conditions are shown with 95% CI. Estimate *P* values were calculated with 2-tailed *t* test. ANOVA for the NTBI, hepcidin, and NTBI-hepcidin interaction terms: *P*_NTBI_ < 0.0001 (F_1,12_ = 113), *P*_hepcidin_ = NS (F_1,12_ = 1.2), *P*_NTBI:_ _hepcidin_ = NS (F_1,12_ = 0.92).

Next, we focused on the contribution of hepcidin to NTBI clearance by monocytes. Blocking of cellular iron export by hepcidin alone could hardly change monocyte LIP (Supplemental Figure 7A). In turn, the combination of NTBI and hepcidin, as compared with NTBI alone, significantly enhanced LIP accumulation in classical and intermediate monocytes but not in the nonclassical subset (Figure 5B). Analogically, hepcidin was found to augment NTBI scavenging in cultures of CD14^+^ monocytes (Figure 6A). In this experiment, both control- and hepcidin-challenged monocytes imported NTBI at a comparable initial speed. In control cells, however, iron export overbalanced the uptake over time. In hepcidin-stimulated monocytes, the net NTBI import was sustained, resulting in a doubled cumulative NTBI clearance (0.9 units in control vs. 1.8 units in hepcidin-stimulated cells, Figure 6A).

Labile cellular iron can be removed from the cell via FPN1 and/or incorporated into ferritin. This second path is preferentially triggered when LIP abundance exceeds export capacities or FPN1 stays inactive due to high hepcidin concentrations (1, 4, 14). In the studied time frame (4 hours), monocyte ferritin levels remained virtually unchanged in cells stimulated with NTBI, hepcidin, or the combination thereof (Supplemental Figure 8). However, NTBI strikingly increased iron incorporation into the existing ferritin molecules in classical and intermediate cells during the 4-hour exposure. This effect was further substantially enhanced by hepcidin in 3 of 4 studied monocyte donors (Figure 6B).

Altogether, we describe classical and intermediate monocytes as robust NTBI scavengers, with a superior capability of LIP accumulation and iron storage in ferritin-bound form that is further bolstered by hepcidin.

### NTBI import in classical monocytes involves reductive and nonreductive routes and is proton coupled.

**Figure 7:**
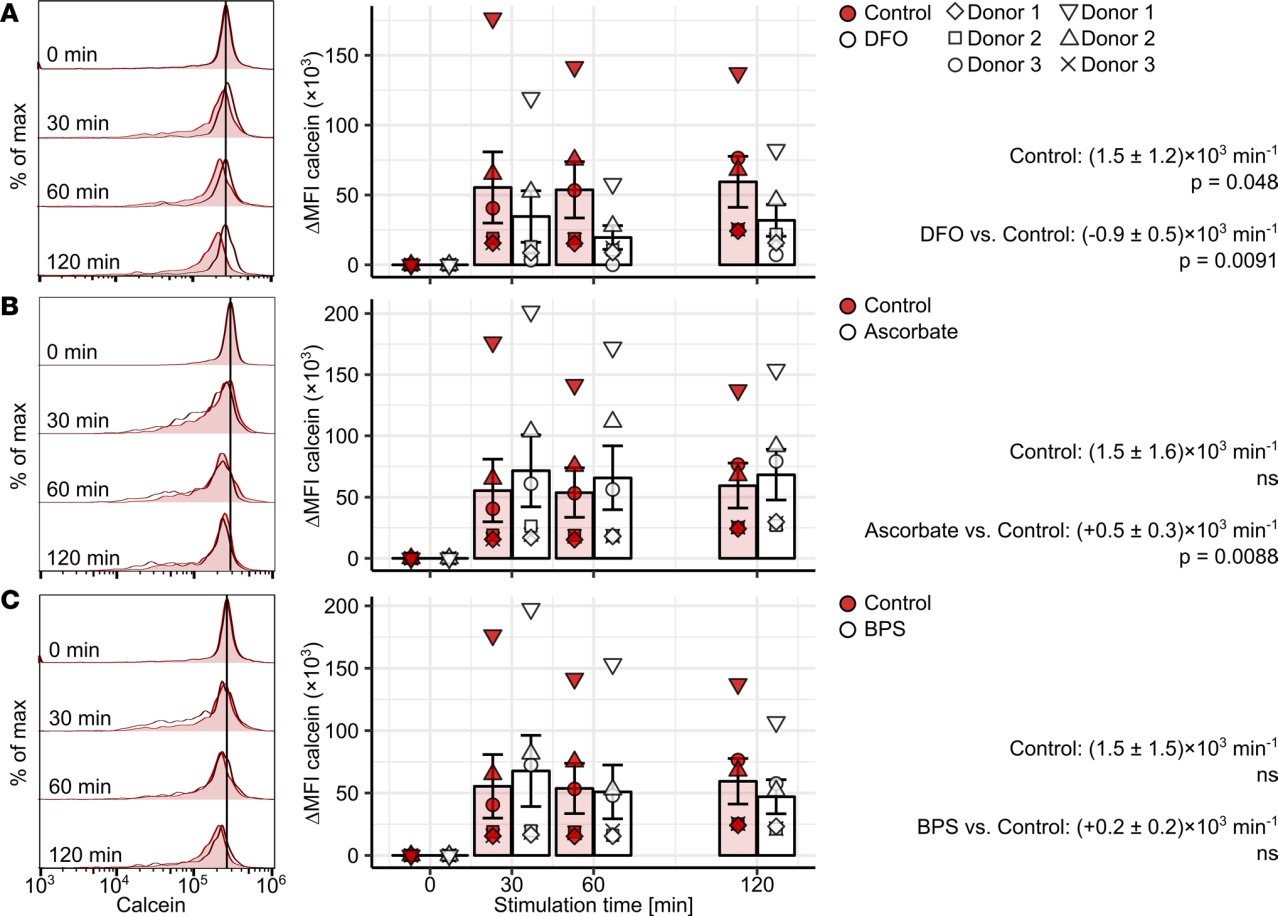
Activity of iron reduction–dependent and –independent NTBI uptake pathways in classical monocytes. Calcein-labeled PBMCs (*n* = 5 healthy individuals) were cultured in presence of 10 μM Fe^3+^ [Fe_2_(SO_4_)_3_] with/without DFO (100 μM, **A**), L-ascorbate (100 μM, **B**), or BPS (100 μM, **C**) for the indicated time points. Monocyte subpopulations were defined as described in Supplemental Figure 2A. Calcein fluorescence in classical monocytes was measured by flow cytometry. Representative calcein signal histograms are shown (tinted histograms: Fe^3+^-only-stimulated cells, open histograms: costimulation). Graphs show ΔMFI values. Each point represents 1 measurement, bars denote mean, and error bars represent SEM. The cell donor is represented by symbol shape. Rate of calcein ΔMFI change was determined with second-order linear models. Estimates for calcein quenching rate in Fe^3+^-only-stimulated cells and differences in quenching rate between Fe^3+^-only and costimulated cells are shown with 95% CI. Estimate *P* values were calculated with 2-tailed *t* test. ANOVA statistics are presented in Supplemental Table 5.

To study the NTBI uptake pathways in classical cells, we treated calcein-loaded monocytes with 10 μM Fe^3+^ NTBI and known modulators of iron import. Canonically, ferric NTBI needs to be reduced for the transport via DMT1 (32, 41) or ZIP14 (33) (Supplemental Figure 9A). In classical monocytes, NTBI-induced LIP expansion was effectively downregulated by the cell-impermeable ferric iron chelator deferoxamine (DFO) and, hence, depended on Fe^3+^ presence (Figure 7A). Iron reduction by ascorbate moderately augmented the uptake (Figure 7B). In contrast, the cell-impermeable Fe^2+^-specific chelator bathophenanthrolinedisulfonate (BPS) could hardly affect the NTBI import (Figure 7C). Based on these observations, we speculated, that NTBI may be taken up to some extent without prior extracellular reduction.

**Figure 8:**
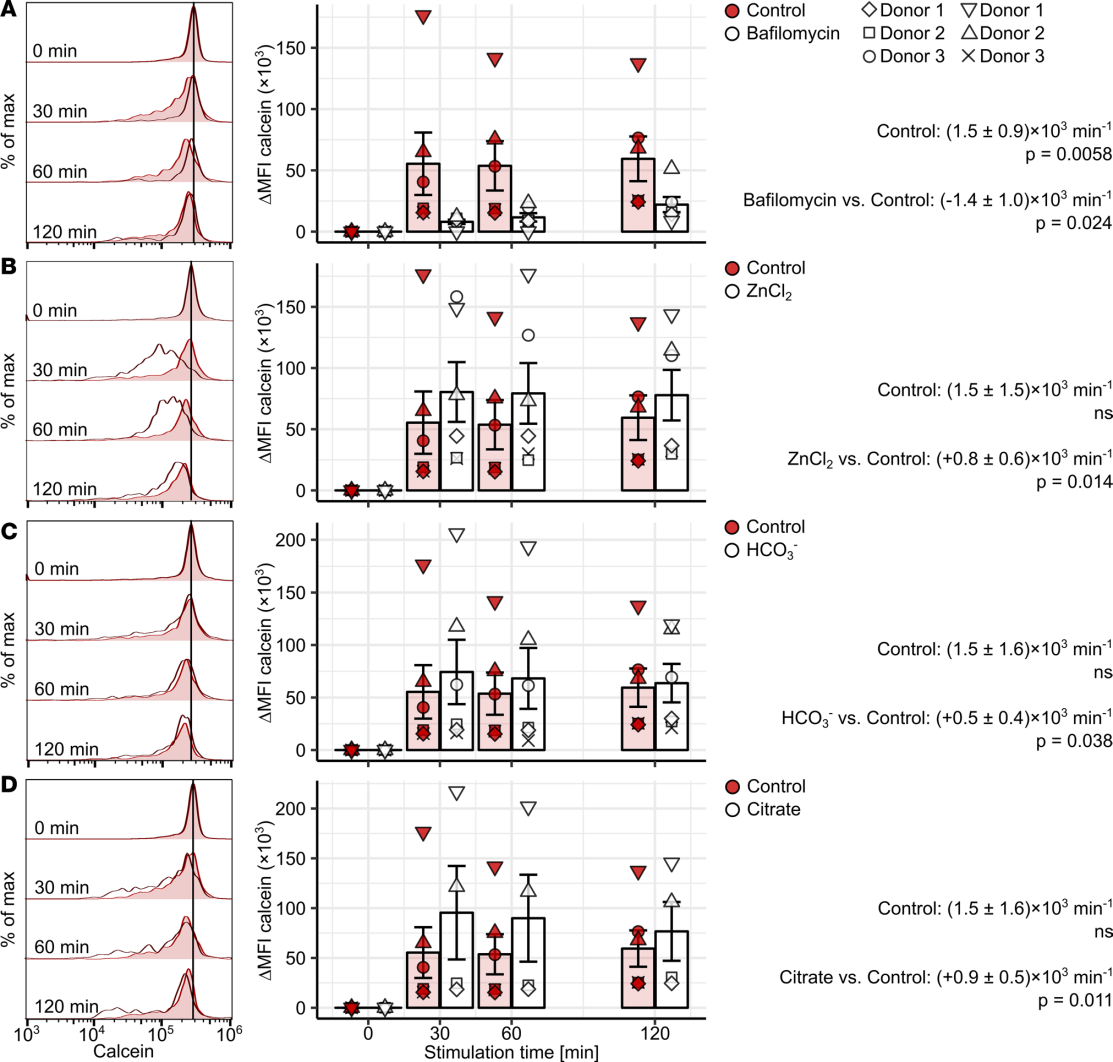
Activity of DMT1-, ZIP-, and citrate-dependent NTBI uptake pathways in classical monocytes. Calcein-labeled PBMCs (*n* = 5 healthy individuals) were cultured in presence of 10 μM Fe^3+^ [Fe_2_(SO_4_)_3_] with/without bafilomycin A (0.5 μM, **A**), ZnCl_2_ (100 μM, **B**), NaHCO_3_ (bicarbonate, 100 μM, **C**), or citrate (10 μM, **D**). Monocyte subpopulations were defined as described in Supplemental Figure 2A. Calcein fluorescence in classical monocytes was measured by flow cytometry. Representative calcein signal histograms are shown (tinted histograms: Fe^3+^-only-stimulated cells; open histograms: costimulation). Graphs show ΔMFI values. Each point represents 1 measurement, bars denote mean, and error bars represent SEM. The cell donor is represented by symbol shape. Rate of calcein ΔMFI change was determined with second-order linear models. Estimates for calcein quenching rate in Fe^3+^-only-stimulated cells and differences in quenching rate between Fe^3+^-only and costimulated cells are shown with 95% CI. Estimate *P* values were calculated with 2-tailed *t* test. ANOVA statistics are presented in Supplemental Table 5.

Iron import via DMT1 is proton coupled (32, 41). Addition of the V-ATPase proton pump inhibitor bafilomycin A dramatically reduced the LIP expansion (Figure 8A). This suggests that the uptake in classical monocytes is a proton-facilitated process, likely involving DMT1. ZIP proteins function as bicarbonate-metal shuttles, with iron import activity that can be bolstered by HCO_3_^–^ and blocked by excess Zn^2+^ (33) (Supplemental Figure 9A). We expected, hence, that a 10-fold molar excess of Zn should inhibit NTBI uptake, but instead we found a significant synergistic uptake stimulation (Figure 8B). Note, that Zn^2+^ alone does not cause calcein quenching (see Supplemental Figure 9B). NTBI-bicarbonate cotreatment increased LIP to some extent compared with NTBI alone (Figure 8C). Taken together, it is unlikely, that transporters with higher affinity toward Zn than Fe, such as ZIP, participate in the NTBI import in classical monocytes.

In a study on T lymphocytes, Arezes and colleagues could demonstrate the existence of a DMT- and ZIP-independent, citrate-facilitated NTBI uptake pathway (40) (Supplemental Figure 9A). In our hands, combination of NTBI with equimolar exogenous citrate robustly elevated the LIP expansion rate, suggesting a possible involvement of a ferric citrate transporter in the monocyte-mediated NTBI uptake (Figure 8D). Collectively, NTBI import in classical monocytes is a proton-, citrate-, and, surprisingly, zinc-stimulated process, which only partially relies on the iron extracellular reduction.

### Monocyte FPN1 and LIP are regulated by cellular and systemic iron levels in iron overload disorders.

Next, we intended to corroborate the hypothesis stating that classical and, possibly, intermediate monocytes take part in iron turnover in steady state and disease. To this end, we investigated surface FPN1 expression and LIP levels in monocytes from healthy individuals, subjects with genetic overload conditions and myelodysplastic syndrome (MDS) patients. Of note, each study group demonstrated a similar monocyte subtype distribution pattern (Supplemental Figure 10, A and B); plasma NTBI could not be detected in any of the studied individuals (data not shown).

**Figure 9:**
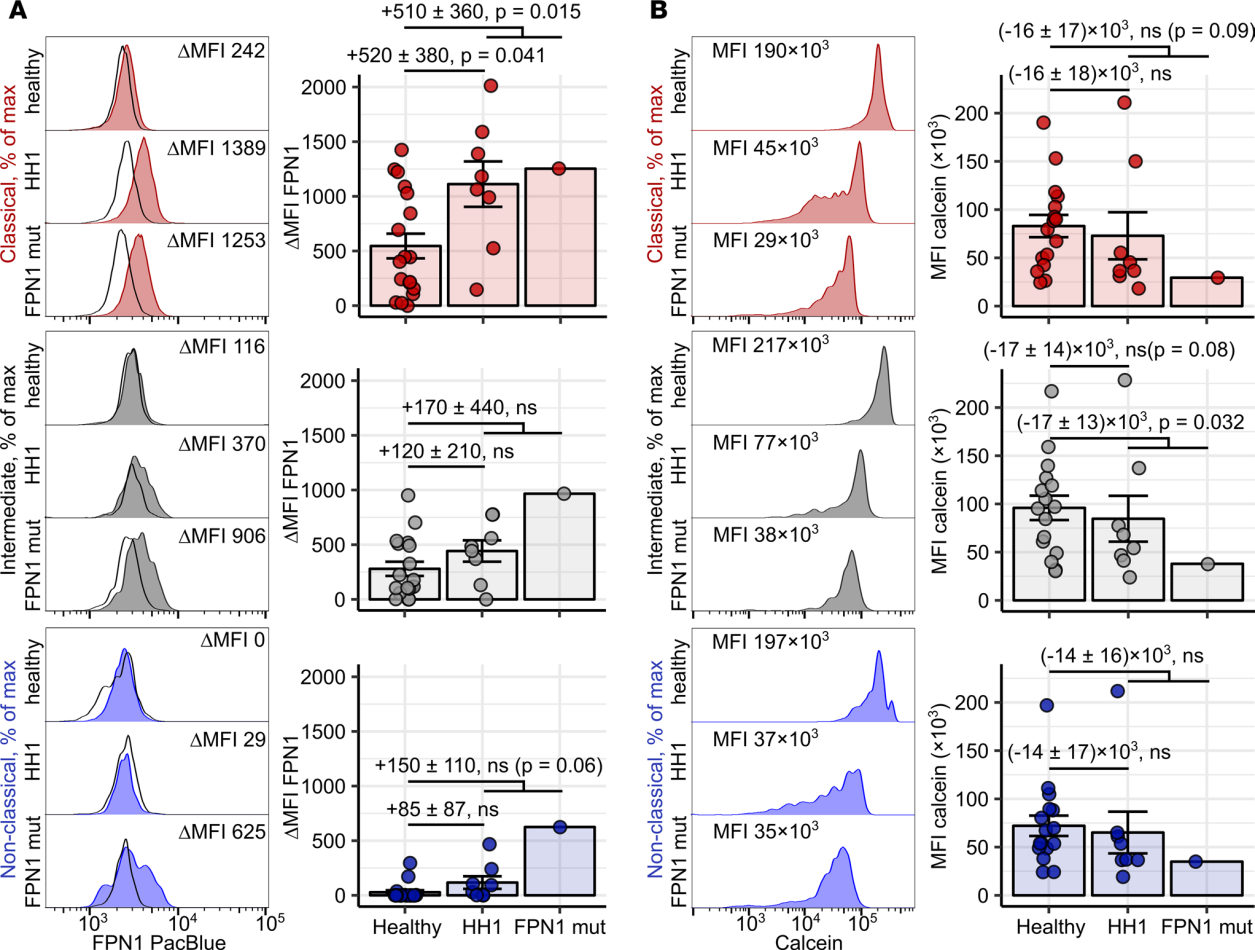
Accumulation of labile iron and regulation of FPN1 in blood monocytes in iron overload diseases. (**A** and **B**) Blood samples (FPN: whole-blood leukocytes, calcein: PBMCs) were obtained from healthy controls (*n* = 18), type 1 hemochromatosis patients (HH1, *n* = 8), and a FPN1 loss-of-function heterozygous individual (FPN1 mut, *n* = 1). Surface FPN1 expression (**A**) and calcein fluorescence (**B**) in monocyte subpopulations were determined by flow cytometry. Monocyte subpopulations were defined as described in Supplemental Figure 10AB (red: classical; gray: intermediate; blue: nonclassical monocytes). Calcein MFI is assumed to be inversely proportional to LIP levels. Representative signal FPN1 and calcein histograms are shown (tinted histograms: specific staining, open histograms: isotype). Graphs show ΔMFI and MFI values. Each point represents 1 measurement, bars denote mean, and error bars represent SEM. Statistical significance for the healthy vs. HH1 and healthy vs. iron overload (HH1 and FPN1 mutant) comparisons were determined with first-order linear models. Separate models were applied to each monocyte subset. Estimate values with 95% CI and *P* values are shown. Estimate *P* values were calculated with 2-tailed *t* test. ANOVA statistics are presented in Supplemental Table 6.

In type I hemochromatosis (HH1), loss-of-function mutations in the *HFE* gene lead to hepcidin deficiency and systemic iron overload, which manifests mostly in the nonleukocytic tissue compartment (42). Classical FPN1 disease is caused by mutations in the FPN1-coding *SLC40A1* gene (e.g., *V162del*), which reduce iron transport efficiency of FPN1 without affecting its hepcidin sensitivity (42–44). As a result, excessive iron accumulation in organ-resident leukocytes can be observed in the affected individuals (42, 43). In our study, HH1 individuals displayed clearly elevated systemic iron levels in comparison to healthy participants, as demonstrated by increased TF saturation and serum ferritin levels, with concomitantly reduced hepcidin concentrations. In turn, the amounts of serum ferritin and hepcidin were substantially higher in the FPN1 mutation carrier than in healthy controls, while TF saturation stayed within the normal range (Supplemental Figure 11A and Supplemental Table 9). In accord with the observed iron and hepcidin sensitivity of the classical monocyte FPN1 (Figure 4), surface FPN1 levels in the classical subset were significantly higher in the HH1 group than in controls. Additionally, in HH1 donors, FPN1 was detectable in the remaining monocyte subsets (Figure 9A). Surprisingly, the FPN1 levels in all monocyte subsets of the FPN1 disease patient were greatly elevated, despite the presence of increased amounts of serum hepcidin (Figure 9A and Supplemental Figure 11A). Monocytes from the majority of HH1 patients (6 of 8) and the FPN1 disease individual showed a profound reduction of calcein fluorescence, indicating elevated LIP (Figure 9B). Of note, a similar pattern of calcein signal dilution was found in iron-challenged monocytes (Figure 5). Furthermore, the greatest differences in LIP between blood leukocytes from healthy controls and genetic iron overload subjects were discerned in monocytes, followed by neutrophils and B cells. In contrast, similar LIP levels were observed in T cells of healthy and affected donors (Supplemental Figure 11C). This suggests superior iron uptake abilities of blood monocytes and corroborates the results of our in vitro NTBI uptake experiments (Supplemental Figure 5B).

**Figure 10:**
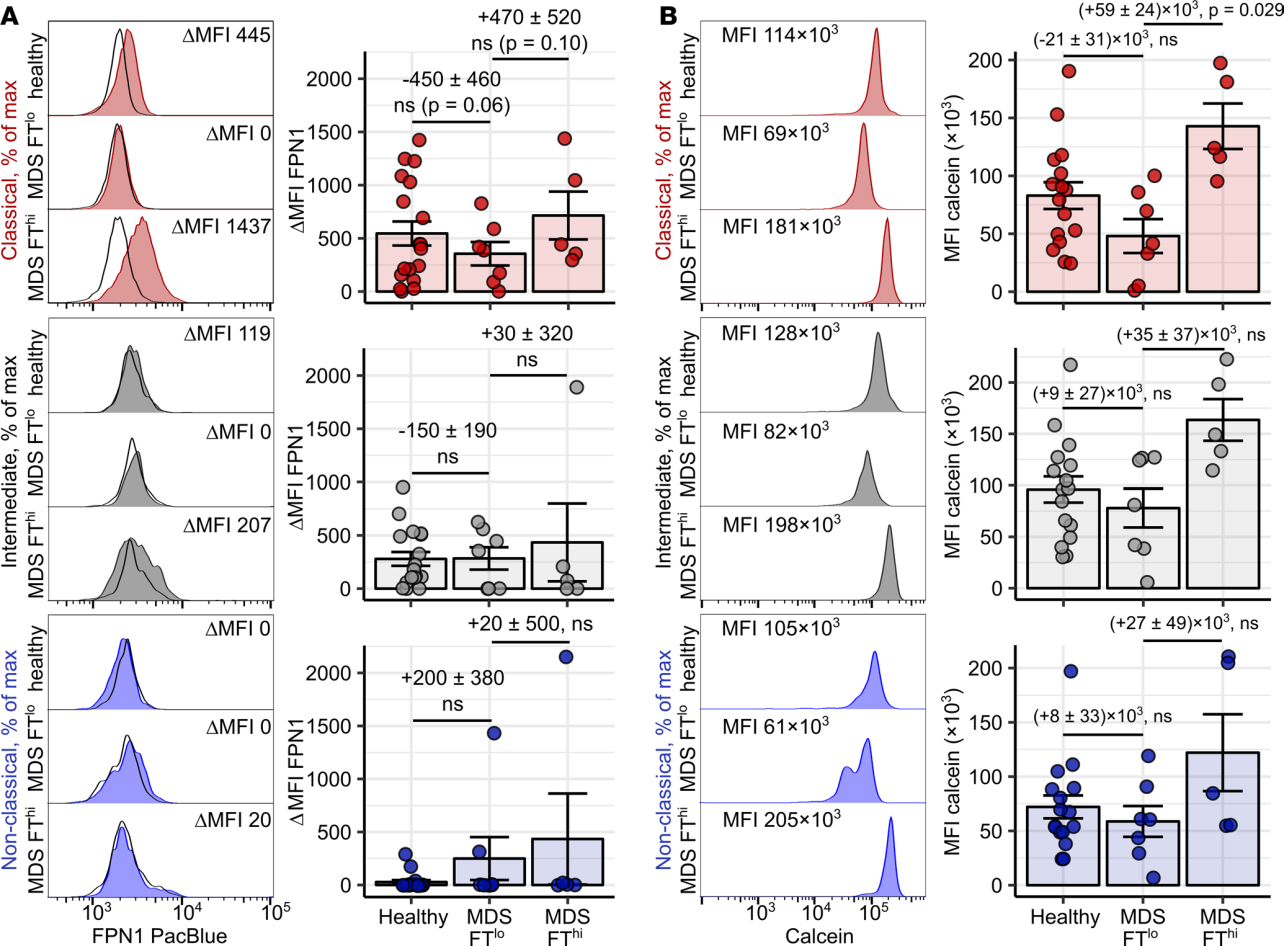
Accumulation of labile iron and regulation of FPN1 in blood monocytes in iron overload diseases. (**A** and **B**) Blood samples (FPN: whole-blood leukocytes, calcein: PBMCs) were obtained from healthy controls (*n* = 18) and myelodysplastic syndrome (MDS) patients with or without hyperferritinemia (FT^lo^: serum ferritin <400 ng/ml, *n* = 7 and FT^hi^: serum ferritin <400 ng/ml, *n* = 5). Surface FPN1 expression (**A**) and calcein fluorescence (**B**) in monocyte subpopulations were determined by flow cytometry. Monocyte subpopulations were defined as described in Supplemental Figure 10AB (red: classical; gray: intermediate; blue: nonclassical monocytes). Calcein MFI is assumed to be inversely proportional to LIP levels. Representative signal FPN1 and calcein histograms are shown (tinted histograms: specific staining, open histograms: isotype). Graphs show ΔMFI and MFI values. Each point represents 1 measurement, bars denote mean, and error bars represent SEM. Statistical significance for the healthy vs. MDS FT^lo^ and MDS FT^lo^ vs. MDS FT^hi^ comparisons were determined with first-order linear models. Separate models were applied to each monocyte subset. Estimate values with 95% CI are shown. Estimate *P* values were calculated with 2-tailed *t* test. ANOVA statistics are presented in Supplemental Table 6.

MDS comprises a spectrum of bone marrow dysplastic conditions manifesting with anemia, myelocytopenia, and systemic iron overload caused by defective erythropoiesis and therapy with RBC transfusions (45–47). To study the effect of iron overload on FPN1 and LIP levels in monocytes of MDS subjects, we dichotomized the patient collective according to serum ferritin (ferritin <400 ng/ml and ferritin >400 ng/ml), which is a widely accepted readout of iron overload in MDS (45, 46). In our study, the entire MDS group displayed signs of ineffective erythropoiesis, such as lowered hemoglobin and RBC counts, paralleled by high serum ferritin, TF saturation, and hepcidin concentration. The signs of systemic iron overload were the most accentuated in patients with hyperferritinemia (Supplemental Table 10 and Supplemental Figure 11B). Surface FPN1 in classical monocytes from the normal-range ferritin MDS group was substantially downregulated in comparison to healthy controls and high ferritin MDS subjects (Figure 10A). Calcein fluorescence measurements in monocytes indicate the highest LIP levels in cells from the normal-range ferritin MDS patients (Figure 10B). Strikingly, monocytes of the high-ferritin MDS group were found labile-iron poor, which might be explained by the restoration of FPN1 expression and export capacities (Figure 10).

Thus, in genetic iron overload conditions and MDS, classical monocyte FPN1 levels are primarily affected by cellular and systemic iron stores rather than by hepcidin concentrations. Furthermore, monocyte LIP seems to depend on the functionality and amount of cellular FPN1, along with the size of systemic iron stores.

### Damaged and senescent erythrocytes are ingested by classical and intermediate monocytes.

Monocyte subpopulations, particularly the classical subset, were found to express molecules involved in erythrophagocytosis, such as CD172a (Figure 3 and Supplemental Figure 3). Hence, we sought to investigate if human blood monocytes may supervise the integrity of the erythrocyte compartment by screening for and removing dysfunctional and senescent RBCs.

**Figure 11:**
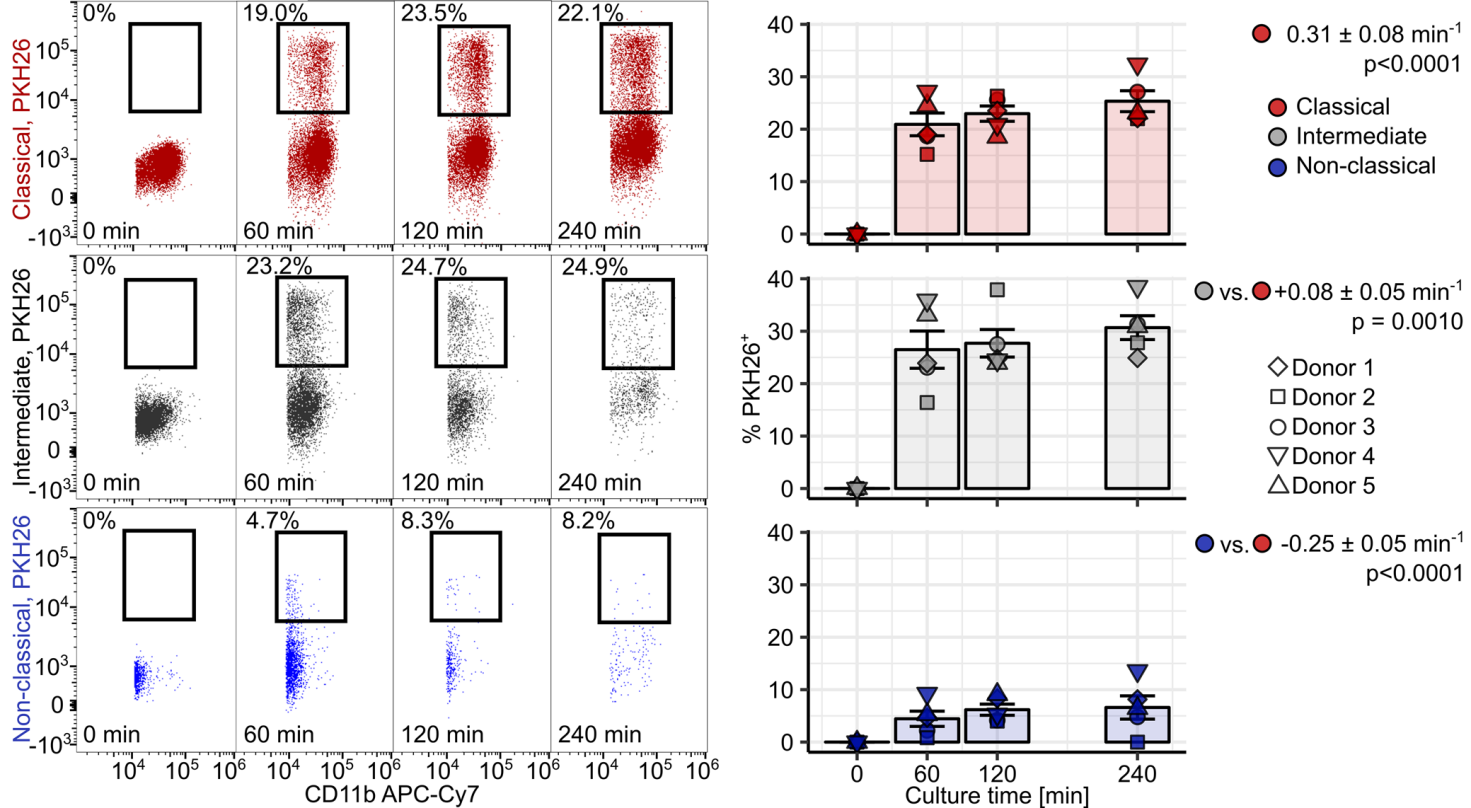
Uptake of damaged erythrocytes by human monocytes in vitro. PBMCs were incubated with PKH26-labeled heat-stressed RBCs (**A**, *n* = 5 healthy donors) at a ratio of 10 RBCs per PBMC for the indicated time points. PKH26 positivity in monocyte subpopulations was determined by flow cytometry. Monocyte subpopulations were defined as described in Supplemental Figure 2A (red: classical; gray: intermediate; blue: nonclassical monocytes). Representative cytometry plots are shown. Graphs show percentages of PKH26^+^ cells in each monocyte subset. Each point represents 1 measurement, bars denote mean, and error bars represent SEM. The donor is represented by symbol shape. RBC uptake rate in classical monocytes and differences in uptake rate between classical monocytes and the particular subset were determined with a second-order linear model. All estimates are shown with 95% CI. Estimate *P* values were calculated with 2-tailed *t* test. ANOVA statistics are presented in Supplemental Table 7.

**Figure 12:**
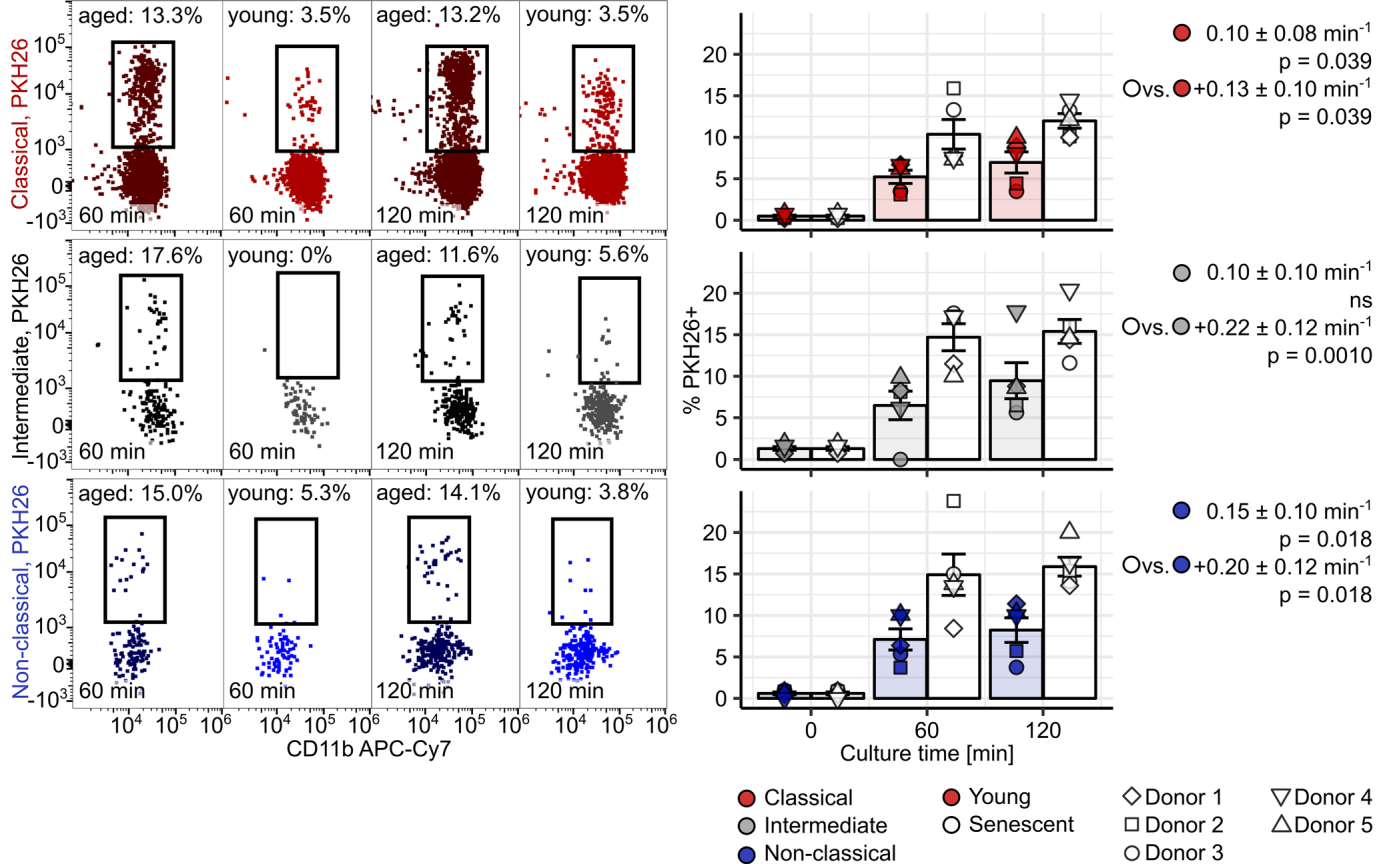
Uptake of senescent erythrocytes by human monocytes in vitro. PBMCs were incubated with young or physiologically senescent PKH26-labeled erythrocytes (*n* = 5 healthy donors) at a ratio of 10 RBCs per PBMC for the indicated time points. PKH26 positivity in monocyte subpopulations was determined by flow cytometry. Monocyte subpopulations were defined as described in Supplemental Figure 2A (red: classical; gray: intermediate; blue: nonclassical monocytes). Representative cytometry plots are shown. Graphs show individual percentages of PKH26^+^ cells in each monocyte subset (colored symbols: young RBCs; open symbols: senescent RBCs). Each point represents 1 measurement, bars denote mean, and error bars represent SEM. The donor is represented by symbol shape. RBC uptake rate in cells cultured with young RBCs and differences in uptake rate between cells cultured with senescent and young RBCs were determined with second-order linear models (a separate model was applied to each monocyte subset). All estimates are shown with 95% CI. Estimate *P* values were calculated with 2-tailed *t* test. ANOVA statistics are presented in Supplemental Table 7.

First, we assessed the ability of healthy donor monocytes to phagocytose PKH26-labeled RBCs damaged by heat exposition (26, 27). With extracellular staining for the RBC-specific marker CD235a, we could demonstrate that classical and intermediate cells not only associated with stressed erythrocytes, but also phagocytosed them. Nonclassical monocytes, in turn, were found predominantly to adhere to erythrocytes without internalizing them (Supplemental Figure 12). Accordingly, classical and intermediate cells phagocytosed heat-stressed RBC with the fastest kinetics (Figure 11). Next, similar uptake assays were performed with senescent RBCs isolated by density gradient centrifugation from whole blood of healthy individuals. In this setting, senescent erythrocytes, rather than young red cells were phagocytosed by monocytes. In contrast to heat-stressed RBCs, the rate of senescent erythrocyte uptake was comparable among monocyte subtypes (Figure 12). Interestingly, the uptake of heat-stressed and senescent RBCs by classical and intermediate monocytes was essentially unaffected by addition of blocking antibodies against 2 prominent RBC receptors, CD172 (34) and TIM4 (35), and the “eat-me” signaling molecule CD47 (48) (Supplemental Figure 13).

**Figure 13:**
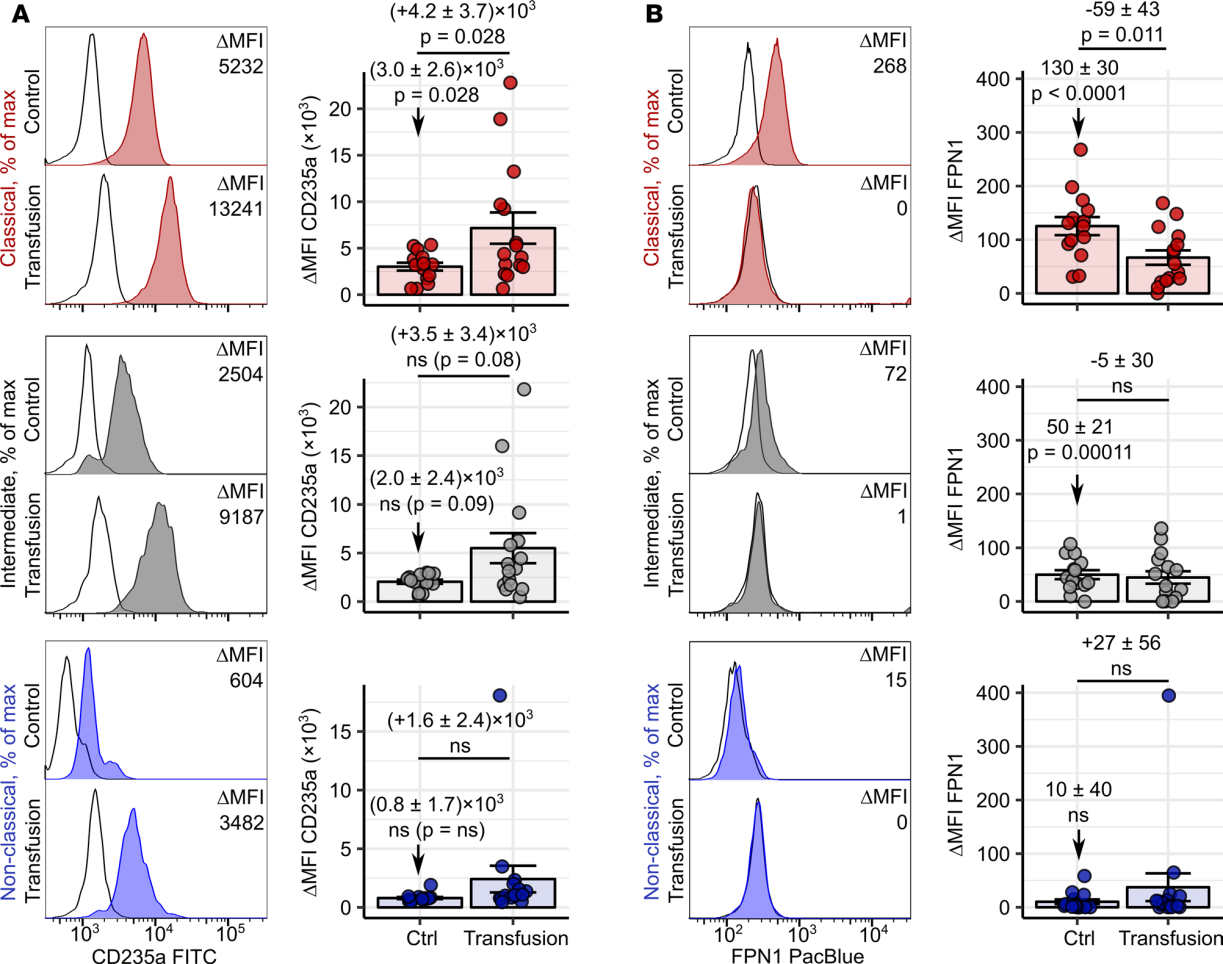
Erythrophagocytosis by blood monocytes in homeostasis and under hematological stress. PBMCs were obtained from healthy individuals (*n* = 16) and patients who had received blood transfusions within the past 24 hours (*n* = 15). Intracellular CD235a levels (**A**) and surface FPN1 expression (**B**) in each monocyte subpopulation were determined by flow cytometry. Monocyte subpopulations were defined as described in Supplemental Figure 14AB (red: classical; gray: intermediate; blue: nonclassical monocytes). Representative CD235a and FPN1 histograms are shown (tinted histograms: specific antibody staining; open histograms: isotype). Graphs show ΔMFI values. Each point represents 1 measurement, bars denote mean, and error bars represent SEM. Statistical significance for basal CD235a and FPN1 levels in controls and the control-transfused differences were determined with first-order linear models. Separate models were applied to each monocyte subset. Estimate values with 95% CI are shown. Estimate *P* values were calculated with 2-tailed *t* test. ANOVA statistics are presented in Supplemental Table 8.

To verify these results in an ex vivo system, we established intracellular staining for the erythrocyte marker CD235a in blood monocytes. Notably, CD235a was localized predominantly inside the cell, as shown by a far higher signal intensity in permeabilized than in intact monocytes, indicating RBC phagocytosis rather than association (Supplemental Figure 14). In line with the in vitro erythrophagocytosis assay results (Figures 11 and 12), we found most intracellular CD235a in classical and intermediate monocytes of healthy donors and only traces of the RBC marker in the nonclassical subset (Figure 13A and Supplemental Figure 14). This suggests that classical and intermediate cells can ingest circulating RBCs under steady-state conditions. Blood transfusions are unavoidably associated with the delivery of numerous damaged red cells into the circulation (2, 16). In transfused individuals, the CD235a staining intensity in classical, intermediate, and, in part, in nonclassical monocytes was increased compared with that of healthy cell donors (Figure 13A). This implies that blood monocytes may augment their RBC uptake capabilities during hematological stress.

FPN1 upregulation and erythrocyte uptake in macrophages are intimately interwoven phenomena (2–4). We could, however, observe reduced FPN1 levels in classical monocytes of transfused patients (Figure 13B). This discrepancy may be explained by the inflammatory background and/or iron deficiency in the oncology patient cohort (Supplemental Table 11) as well as systemic inflammation caused by the transfusion itself, culminating in high hepcidin serum concentration (2, 13, 16).

In sum, we provide strong evidence that human blood monocytes, in particular classical and intermediate ones, act as professional erythrophagocytes in steady state and under transfusion-induced hematological stress.

## Discussion

Our microarray data analysis and gene and protein expression studies as well as functional assays revealed distinct iron-handling phenotypes of 3 monocyte subsets. Classical monocytes expressed sustained levels of ferritin, TfR1, the putative NTBI importers DMT1 (33) and ZIP14 (32), and the RBC receptor CD172a. The characteristic feature of this population was the highest surface expression of hepcidin-sensitive, functional FPN1 as compared with other subpopulations. The classical monocyte FPN1 levels were transiently upregulated by iron in general and particularly by NTBI. Notably, regulation of human monocyte FPN1 by inflammation and systemic and autocrine hepcidin has been reported previously (6, 13). Furthermore, we could prove excellent TBI and NTBI uptake capabilities of classical monocytes. In terms of the iron-related transcript and protein repertoire, intermediate monocytes ranked between classical and nonclassical cells. Functionally, the intermediate subset demonstrated similar handling of TBI and NTBI to the classical population. However, intermediate cells displayed high ferritin levels and only sparse surface FPN1 expression, suggesting rather inefficient iron export and preferential iron storage. The nonclassical monocytes showed the lowest levels of iron-related transcripts. Their readily detectable expression of the iron importer ZIP14 as well as TfR1 did not translate into efficient NTBI and TBI uptake.

Import of NTBI into the cell is believed to be accomplished by 2 ferrous iron transporters, DMT1 (32) and ZIP14 (33), which require reduction of ferric NTBI. Our observations made in classical monocytes exposed to NTBI along with the strong reductant ascorbate or the cell-impermeable ferrous chelator BPS suggest, however, that only a part of NTBI is taken up in ferrous form. Furthermore, the addition of the extracellular ferric chelator DFO could, in turn, block 60% of the uptake. A 10-fold molar excess of Zn^2+^ boosted NTBI import in classical monocytes instead of inhibiting it, and HCO_3_^–^ addition only slightly improved the uptake arguing against the contribution of ZIP14 (33). To our knowledge, a synergy between Zn and Fe cellular import, rather than an inhibition, has been described in only very few publications. Yamaji et al. found such an interaction for the DMT-mediated import pathway in an enterocyte cell line (49). Bishop and colleagues reported it for NTBI-challenged astrocytes (50). However, mechanistic details of this process still call for an explanation. As evidenced by experiments with a proton pump inhibitor, NTBI import in classical cells is proton dependent, suggesting the involvement of DMT1 (32, 41) or another proton-driven iron shuttle. Since the uptake was enhanced by equimolar concentrations of citrate this latter NTBI transporter could function as a Fe^3+^: citrate symporter, as proposed by Arezes et al. (40). Collectively, we postulate that NTBI scavenging in classical monocytes employs both a reductive route, most likely via DMT1, and a nonreductive pathway. The existence of such a “noncanonical” Fe^3+^ import mechanism was proposed by several reports (50–54) and supported by the fact that the NTBI-induced cytoplasmic LIP consists of both Fe^2+^ and Fe^3+^ (14, 51). Alternatively, the NTBI import may involve a reduction step which takes place in intracellular, BPS- and ascorbate-inaccessible compartments. The data of Sohn and colleagues suggesting the uptake of ferric iron: citrate: albumin complexes by macropinocytosis may also pose a mechanistic explanation for the NTBI import in classical monocytes (55).

As already stressed, NTBI and damaged erythrocytes exert direct toxicity toward cells and organs (2, 15, 16) and increase iron availability to pathogens (11, 12, 18, 19). Hepcidin has recently been postulated to orchestrate NTBI clearance in response to danger signals and mediate protection against infections with siderophilic bacteria (11, 12). Similarly, in humans exposed to high doses of oral iron, the iron-mediated induction of hepcidin excellently correlated with NTBI clearance from the circulation (56). However, the specific cell type removing NTBI in response to hepcidin has not been identified so far. Three points speak in favor of classical monocytes as such candidate hepcidin effectors: (a) the surface expression of active, hepcidin-sensitive FPN1; (b) an exceptionally fast, hepcidin-boosted LIP increase and scavenging of NTBI from the surrounding; and (c) preferential uptake of NTBI, even in the presence of abundant holo-TF. Conceivably, high circulating monocyte numbers, e.g., in course of inflammation or infection, may facilitate the removal of NTBI and damaged RBCs locally as they occur — which could not be easily accomplished by known sessile scavenging cells, such as hepatocytes (57), liver and spleen macrophages (2, 16). In our hands, classical monocytes showed the fastest LIP accumulation rates upon NTBI challenge among the major blood leukocyte types. CD4^+^ and CD8^+^ T cells described before as NTBI scavenging cells (39, 40) demonstrated only a moderate LIP increase. Importantly, the largest relative expansion of LIP in monocytic cells of genetic iron overload patients supports these in vitro data. However, to evaluate the contribution of quantitatively different circulating and sessile leukocyte populations to the systemic NTBI clearance, depletion of the particular populations, e.g., with specific antibodies, would be required. This intervention is only possible in a murine model with known confounding differences in monocyte biology (58), for example, no clearly defined intermediate population and absence of FPN1 expression in classical monocytes under steady state (2).

Data obtained in our study with iron overload due to genetic disorders, bone marrow dysplasia, and blood transfusion corroborate the role of monocytes in iron turnover and removal of toxic iron forms. Steadily increased iron availability together with lowered hepcidin levels in HH1 patients (42) culminated in a doubling of classical monocyte FPN1 levels. Notably, in HH1 individuals, the FPN1 upregulation may not compensate for the elevated iron uptake, resulting in a LIP increase in the major monocyte subset. Analogically elevated LIP levels were also detected in the remaining monocyte subtypes in HH1 and FPN1 disease subjects, which can be explained by the relative longevity of these cells and prolonged exposition to iron (59). The observed labile iron accumulation in leukocytes from HH1 patients apparently contradicts previous reports describing rather a reduced import (30, 60), enhanced export (30, 61, 62), and a net iron depletion from monocytes and macrophages (63, 64). This discrepancy may primarily be based on different methodology (calcein vs. activity of the iron-responsive proteins [IRP]) and has been discerned by Lee and colleagues in neurons (65). Differences in cell-specific iron handling may also explain those discrepancies, as most of the previously published experiments were performed with differentiated cells. Apart from that, a drastic upregulation of monocyte ferritin in HH1 is well described, supporting our finding that monocytes indeed accumulate iron in the affected individuals (63, 66). The critical effect of FPN1 activity on the regulation of monocyte LIP levels can be observed in cells carrying the heterozygous loss-of-function *SLC40A1* *V162del* mutation (43, 44). Even in the presence of normal circulating iron amounts (42, 43), monocytes of that patient demonstrated a pronounced LIP rise. In all 3 monocyte subtypes of the *SLC40A1* mutant individual elevated FPN1 levels could be found, despite increased circulating hepcidin amounts. Of note, the *SLC40A1* *V162del* mutation preserves normal hepcidin sensitivity of FPN1 (44). This suggests that regulatory signals mediated by intracellular iron may override the hepcidin-mediated FPN1 inhibition, at least under pathological cellular iron overload.

In MDS, iron overload caused by defective erythropoiesis and therapy with RBC transfusions accompanies disease progression and further worsens the erythroid output (45–47, 67). Serum ferritin was demonstrated to correlate with marrow iron accumulation (47, 67), function (67), and genomic instability (68) in myelodysplasia and is hence regarded as a readout of systemic iron overload in MDS used in diagnosis and therapy decisions (45, 46). Elevated surface FPN1 in classical monocytes was observed in MDS individuals with iron overload defined by hyperferritinemia compared with the MDS group with near-normal ferritin levels. Notably, both MDS group strata had similar serum hepcidin concentrations, which suggests that the presence of iron overload overbalances the negative regulatory signals via hepcidin in this particular condition. Lower FPN1 and, presumably, the reduced iron export rate in the ferritin-high MDS cohort translated into a labile iron accumulation in all monocyte subsets. To our knowledge, no studies investigating iron turnover in blood leukocytes of MDS individuals have been published before. Observations made by Nybakken and colleagues suggest that bone marrow macrophages pose the main site of iron deposition in MDS, even before the onset of transfusional therapy (47). Importantly, macrophage iron deposits significantly correlated with shortened survival (47). The question of whether expression of monocyte iron turnover proteins and LIP correlates with the extent of pathological iron accumulation and response to therapeutic measures, such as phlebotomy or iron chelation, warrants further investigation in a larger patient collective.

Tissue-resident macrophages orchestrate iron homeostasis as professional erythrophagocytes, recycling the hemoglobin-derived iron via FPN1-mediated export (1, 2, 4). The function of circulating monocytes in this context is far less clear. Depending on the context (homeostasis, inflammation, RBC opsonization), classical (2, 26, 27), intermediate (69, 70) as well as nonclassical monocytes (70) were reported as erythrophagocytes. In our experiments, classical and intermediate monocytes demonstrated the superior uptake of heat-stressed RBCs. In contrast, all 3 monocyte subsets endocytosed physiologically altered erythrocytes with a comparable kinetics. Additionally, the uptake of neither heat-stressed nor senescent RBCs could be modulated by blocking of the CD172a- and TIM4-mediated pathways (48). At least 2 other prominent erythrophagocytosis pathways are known, the Band3/IgG-mediated and phosphatidyl serine–mediated (PS-mediated) routes (71). Whereas the Band3 pathway was proposed to mediate removal of senescent RBCs under steady state (71–74) and parasite infection (69, 70, 75), PS exposition was postulated to be an erythrocyte eat-me signal under stress stimuli, such as calcium ionophores or transfusion (71, 76, 77). In our experiments with senescent RBCs, the involvement of the Band3/IgG route may explain similar uptake rates between the subsets, since all 3 monocyte subsets were attributed high expression of diverse IgG receptors (69, 70). Conversely, the Band3 neoantigen formation and IgG opsonization could be excluded in case of heat-stressed RBCs, since neither the temperature shock nor the uptake assay was conducted in presence of human serum (74). The contribution of the PS route to the phagocytosis of stressed RBCs by classical and intermediate cells poses an attractive alternative, which needs to be experimentally verified.

Utilizing an intracellular staining for the erythrocyte marker CD235a, we could demonstrate clearance of aged and stressed RBCs by classical and intermediate monocytes in vivo. Erythrophagocytic activity observed with this technique was the highest in classical and intermediate monocytes and detectable not only in blood-transfused but also in untreated healthy individuals. This suggests that, under steady-state conditions, classical and intermediate monocytes scan the bloodstream for damaged and senescent erythrocytes, remove them, and recycle iron needed for erythropoiesis. The preferential RBC uptake by classical monocyte in vivo apparently contradicts the comparable affinity of all monocyte subtypes toward senescent erythrocytes observed in vitro. However, because of the 10-fold higher absolute numbers of circulating classical monocytes in comparison with nonclassical cells, removal of dysfunctional RBCs by the classical cell is a far more probable event. Furthermore, here and in our previous report (2), we describe augmented erythrophagocytosis by classical and intermediate monocytes in vivo after blood transfusion and cardiopulmonary bypass pointing toward a crucial role of these cells in RBC removal when the number of damaged erythrocytes exceeds the scavenging capacities of tissue-resident phagocytes.

In summary, we provide evidence for cell-specific differences in iron-handling modalities among the 3 monocyte subsets in humans. We identify fairly abundant classical monocytes as FPN1-expressing erythrophagocytes and NTBI scavengers, which suggests their possible role in monitoring the integrity of the erythrocyte compartment and iron homeostasis at the local and, likely, also systemic level. In addition, we put forward classical monocytes as putative hepcidin effector cells in the process of hepcidin-dependent NTBI removal. Notably, intermediate monocytes demonstrate similar iron and RBC uptake capabilities to the major monocyte subset. However, due to their quantitative inferiority, the contribution of the intermediate subset to the systemic iron homeostasis is questionable.

## Methods

### Human study.

Study participants were recruited in 2 batches: the iron overload/MDS and transfusion substudies.

The iron overload/MDS substudy population encompassed healthy individuals (*n* = 18), phlebotomy-treated HH1 patients carrying the homozygous *HFE* *C282Y* mutation (*n* = 8), a classical FPN1 disease subject (heterozygous for the *SLC40A1 V162del* mutation, *n* = 1), and chelation therapy-naive myelodysplastic syndrome (MDS) patients with normal serum ferritin levels (MDS FT^lo^, ferritin <400 ng/ml, *n* = 7) and hyperferritinemia (MDS FT^hi^, ferritin >400 ng/ml, *n* = 5). The transfusion substudy population consisted of healthy control individuals (*n* = 16) and oncological patients from the University Clinic of Innsbruck, who received blood transfusion for study-independent indications 16–24 hours prior to blood withdrawal (*n* = 15). Demographic characteristics and blood parameters of the study participants are summarized in Supplemental Table 9 (genetic iron overload), Supplemental Table 10 (MDS), and Supplemental Table 11 (transfusion).

Healthy volunteers from both substudies served as blood donors in most experiments. Each study participant donated up to 35 ml blood.

### Analysis of microarray data.

For details on bioinformatic analysis of microarray data see the Supplemental Methods. Microarray data analysis was made with R (R Foundation for Statistical Computing). Three publicly available microarray studies, GSE66936 (78), GSE25913 (79), M-EXP-2544 (22) (Supplemental Table 1), were reanalyzed. Significance of expression level differences for 523 iron-related genes between monocyte subsets was determined by 1-way ANOVA with inclusion of the donor effect. *P* values were adjusted for multiple comparisons with the Benjamini-Hochberg method (Supplemental Table 2). Genes with expression differences that were found significant at the *P* < 0.1 level in at least 2 of 3 studies were further investigated. Heatmap visualization (euclidean distance, average linkage algorithm) was performed with Genesis (80). Two-dimensional metric principal coordinate analysis (with the Pearson’s sample-sample distance) for each study was performed with *Z*-scores of expression data.

### Cell isolation, fluorescent staining, and flow cytometry.

Whole blood leukocytes were obtained from whole blood samples by RBC lysis (2). PBMCs were isolated by centrifugation of whole blood samples with Biocoll Separating Solution (density 1.077 g/ml, 300 g, 25 minutes, Biochrom). CD14^+^ cells were isolated from PBMCs with the MACS CD14-positive selection kit (Miltenyi Biotech; purity exceeding 95%, Supplemental Figure 4A). Iron-containing MACS beads were removed from the cell surface by trypsin treatment.

Flow cytometry staining was performed as described previously (2) with antibodies listed in Supplemental Table 12 and appropriate isotype controls. The anti-human FPN1 antibody was generated by Amgen as described previously (81). Intracellular staining was performed with the Fixation/Permeabilization Kit (Becton Dickinson). For determination of LIP levels, cells were labeled with 1 μg/ml CalceinAM (Thermo Fisher) for 5 minutes at 37 °C.

Protein expression was presented as a difference in MFI between the specific and isotype staining (ΔMFI). LIP level change (ΔMFI calcein) was expressed as a difference in calcein MFI between the control (unstimulated or time point 0 minutes) and a particular sample (37, 82). For the endpoint LIP determination in study individuals, calcein fluorescence was expressed as MFI, a value that is assumed to be inversely proportional to LIP.

Data were acquired with the Gallios flow cytometer (Beckman Coulter) and analyzed with FlowJo. For details on cell isolation and flow cytometry, see the Supplemental Methods.

### Cell culture.

PBMCs and monocytic cells were cultured in 10% FCS-containing RPMI1640 medium with penicillin and streptomycin (all from Lonza). Human synthetic hepcidin was purchased from PeptaNova. To induce NTBI formation in medium, addition of 10 μM ferric iron (Fe^3+^) in the form of Fe_2_(SO_4_)_3_ (MilliporeSigma) was routinely used (Supplemental Figure 4D). Other substances (all from MilliporeSigma) used were holo-TF (20 μM), deferoxamine mesylate (100 μM), BPS (100 μM), L-ascorbate (100 μM), bafilomycin A (0.5 μM), ZnCl_2_ (100 μM), NaHCO_3_ (100 μM) and citrate (10 μM).

### RBC uptake assay.

For generation of heat-stressed RBCs, erythrocytes from the pellet fraction after PBMC isolation were incubated at 42°C for 20 minutes. Senescent circulating RBCs were isolated by centrifugation of whole blood over a Percoll gradient series (MilliporeSigma; 1.064–1.080 g/l, 2000 g at 25°C). The densest, senescent RBC-enriched fraction (1.080 g/l, “old” RBC) (83) and the pooled 1.068 and 1.066 g/l fractions (“young” RBC) were utilized in uptake experiments. RBC were labeled with PKH26 (MilliporeSigma) according to the manufacturer’s protocol.

For ex vivo erythrophagocytosis assays, PBMCs were incubated with PKH26-labeled RBCs (10 RBC/1 PBMC) and analyzed by flow cytometry at the indicated time points (2, 26, 27). For details on RBC and holo-TF uptake assays, see the Supplemental Methods.

### ^59^Fe ferritin incorporation assay.

CD14^+^ monocytes were cultured for 4 hours in 10% FSC RPMI1640 with 0.5 μM ^59^Fe^3+^ (containing TBI only) or with a mixture of 0.5 μM ^59^Fe^3+^ and 10 μM ^56^Fe^3+^ (containing NTBI, Supplemental Figure 4D). The cultures were additionally stimulated with 2 μg/ml hepcidin or vehicle. Cell lysates were resolved by electrophoresis and ^59^Fe-ferritin bands (approximately 400 kDa) visualized by autoradiography (84). For details on ^59^Fe uptake and ferritin incorporation experiments see the Supplemental Methods.

### NTBI determination and NTBI scavenging assay.

NTBI in monocyte culture medium samples was determined with the FeROS eLPI Kit (Afferix). Concentration of NTBI was expressed in AU per μl.

For NTBI scavenging experiments, CD14^+^ cells were MACS-purified from PBMCs obtained from 35 ml blood. CD14^+^ monocytes were cultured in 300 μl 10% FCS RPMI1640 with 10 μM Fe^3+^ [Fe_2_(SO_4_)_3_] in sterile Eppendorf tubes. At indicated time points, cell cultures were thoroughly mixed and 50-μl aliquots were removed. Cells were depleted from the aliquots by centrifugation, and the supernatant was used for NTBI determination. The NTBI concentration at the 0 minute time point refers to the cell-free medium. For NTBI uptake rate at the *i*th time point (*t_i_* > 0), the following formula was applied: rate*_i_* = (*C_i_* – *C*_i–1_)/(*t_i_* – *t*_i–1_), where *C_i_* stands for NTBI concentration and *t_i_* for time at the *i*th time point. The uptake rate for 0 minutes was assumed to be 0.

### TF loading assay.

The assay employs differences in absorption spectrum of TF caused by iron binding (36) (Supplemental Figure 4, B and C). MACS-purified CD14^+^ monocytes were incubated with 10 μM Fe^3+^ [Fe_2_(SO_4_)_3_] in 10% FCS RPMI1640 (Lonza) for 1 hour, pelleted by centrifugation and washed 3 times with 1% FCS RPMI1640. Next, the cells were cultured in 250 μl 1% FCS RPMI1640 with 0.5 mg/ml apo-TF (MilliporeSigma) in sterile Eppendorf tubes. At indicated time points, cultures were thoroughly mixed, and 50-μl aliquots were removed. Cells were depleted from the aliquots by centrifugation, and absorbance of the supernatant in the 250–500 nm range was measured on a Tecan Infinite200 plate reader. 1% FCS RPMI1640 medium served as blank control.

### Statistics.

For more details on data visualization and statistical analysis, see the Supplemental Methods. Data plotting and analysis was performed with R (R Foundation for Statistical Computing).

Unless otherwise indicated, data were plotted as point bar plots, where each point represents 1 measurement, bars denote mean, and error bars represent SEM.

For statistical analysis, mixed-effect linear modeling was employed. Categorical variable data were modeled with a first-order model. Rate determination and comparison for saturable kinetic processes (e.g., calcein quenching) was done by fitting experimental data to a second-order linear model, where the first-order term approximates process rate. Random terms in the models included effects the cell donor, donor-sample interaction, and/or donor-time point interaction, as appropriate.

Significance of the model estimates [*P* (β ≠ 0)] was assessed with 2-tailed *t* tests. Significance of the model terms was assessed with ANOVA and log-likelihood ratio tests. Estimate *P* values were corrected for multiple comparisons with the Benjamini-Hochberg method. Model estimates are presented with 95% CI and *P* values. *P* < 0.05 was considered significant.

### Study approval.

Human samples were collected after obtaining written informed consent of the participants. Anonymous primary participant data were accessible to all authors. The study was performed in accordance with the Declaration of Helsinki and approved by the ethics committee at the Medical University of Innsbruck (study UN3468).

## Author contributions

DH, IT, GW, and PT designed the study and interpreted the results. DH, VP, MS, and PT performed experiments and microarray data reanalysis. DH, VP, and PT analyzed anonymous primary participant data. TLA developed the anti-human FPN1 antibody. SS assisted with flow cytometry experiments. IT, DH, CF, FK, TS, RS, BS, HZ, and CT supervised the human study, recruited participants, and gathered informed consents. DH, IT, GW, and PT wrote the manuscript.

## Supplementary Material

Supplemental data

Supplemental Table 2

## Version 1 04/18/2019

Electronic publication

