# Supplementary material for: Classical and intermediate monocytes scavenge non-transferrin-bound iron and damaged erythrocytes
Source: JCI Insight. 2019 Apr 18;4(8):e98867. doi: 10.1172/jci.insight.98867 (PMC6538345; doi:10.1172/jci.insight.98867)
Supplement: Supplemental data [file jciinsight-4-98867-s173.pdf]

Supplementary Material

Classical and intermediate monocytes scavenge non-transferrin-bound iron and damaged erythrocytes

Supplementary Methods

Analysis of microarray data

Microarray data analysis was made with R. Three studies were selected for re-analysis: GSE66936(1), GSE25913(2) (both GEO Repository), M-EXP-2544(3) (Array Express) (Supplementary Table S1). The selection criterion was the availability of whole-genome expression data for all three subsets of human blood monocytes from healthy donors. A list of iron-related gene ontology terms (GO; search term ‘iron’) was obtained from the QuickGO server and the identifiers for corresponding iron-related genes were retrieved with the BioMart of EMBL (BioMart package; Supplementary Figure S1A). Expression data for the iron-related genes were extracted from normalized gene expression tables provided by the study authors.

Statistical significance of the monocyte subset-specific expression differences was assessed with one-way ANOVA with inclusion of the donor effect for each iron-linked gene. P-values for the monocyte population term were adjusted for multiple comparisons with the Benjamini-Hochberg method (see Supplementary Table S2 for p values). Genes, whose expression differences were found significant at the $p < 0.1$ level in at least two out of three studies were further investigated. Heat map visualization was accomplished with Genesis(4) with the average linkage clustering algorithm and euclidean distance measure.

To visualize relationships between samples in each microarray study, two-dimensional metric principal coordinate analysis (PCoA, with the Pearson’s sample-sample distance) was performed with Z scores of expression data. Primary reasons for choosing PCoA instead of principal component analysis (PCA) were: (1) the flexibility in selection of the sample-sample distance measure (with Pearson’s distance performing best) and (2) the a priori assumption of limited dimensionality for the visualization space (2 dimensions), since we intended to resolve only the relationships between 3 monocyte subsets.

To identify iron-related genes with the least variance in expression between monocyte subsets, subset-specific expression variance (MSS, mean sum of squares) was extracted from the ANOVA table (see above) and divided by the mean expression for each gene giving the Mean-Variance Ratio (MVR). 100 genes with the lowest absolute values of MVR and Benjamini-Hochberg-corrected p-values > 0.1 for each study were selected. Genes identified by this method in at least two studies were further investigated.

Cell isolation, fluorescent staining and flow cytometry

For determination of FPN1, TfR1, DMT1 (Divalent Metal Transporter 1), ZIP14 (Zrt- and Irt-like Protein 14), CD172a (Signal regulatory protein α) and TIM4 (T-cell Membrane protein 4) levels in monocytes, whole blood was depleted of RBC by treatment with ACK buffer (150 mM NH_4Cl, 10 mM $KHCO_3$, 0.1 mM EDTA, all from Sigma-Aldrich, Darmstadt, Germany) and the leukocyte fraction was used. For functional assays, intracellular ferritin and CD235a staining, the mononuclear blood fraction (PBMC) was obtained from supernatant interphase after gradient centrifugation at 300g for 25 minutes with Biocoll Separating Solution (density 1.077 g/ml, Biochrom, Berlin, Germany). $CD14^+$ monocytes were isolated from PBMC with the MACS CD14 positive selection kit (Miltenyi Biotech, Bergisch Gladbach, Germany). Iron-containing MACS beads were removed from the cell surface by a 10 minute treatment with 0.5% trypsin (Gibco, Thermo Fischer, Waltham, MA) followed by a culture medium wash. The final monocyte preparation contained classical and intermediate cells at the purity exceeding 95% (Supplementary Figure S4A).

Flow cytometry staining was performed as described(5) with antibodies listed in Supplementary Table S12 and appropriate isotype controls. The anti-human FPN1 antibody was generated by Amgen (Thousand Oaks, CA) as described(6). The intracellular staining for CD235a and ferritin was performed with the Fixation/Permeabilization Kit (Beckton Dickinson, Franklin Lakes, NJ). To

increase the specificity of the intracellular staining, PBMC were co-stained for CD15 along with monocyte markers in order to remove residual granulocytes. For LIP determination in cultured cells, cells were extensively washed with PBS and loaded with 1 µg/ml CalceinAM (Thermo Fisher, Waltham, MA) in PBS at 37 ºC for 5 minutes prior to culture. For the end-point LIP determination in study participants' cells, PBMC were isolated from heparinized blood (to avoid any result distortion by chelators like EDTA or citrate), loaded with calcein as described above, stained with antibodies and measured within 30 minutes.

Expression of TfR1, FPN1, CD172a, TIM4, DMT1, ZIP14, ferritin and CD235a in a particular monocyte subtype was presented as difference in median fluorescence intensity (MFI) between the specific and isotype staining (ΔMFI). In functional assays, calcein ΔMFI was calculated as a difference in calcein MFI between the control (unstimulated sample or time point 0 minutes) and a particular sample and served as a measure of iron-induced LIP change in the cell(7–9). For the end-point LIP determination in study individuals, calcein fluorescence was expressed as MFI, whose value is assumed to be inversely proportional to LIP(7).

DAPI or 7AAD counterstain served for live-dead discrimination in extracellular staining. Data were acquired with the Gallios flow cytometer (Beckman Coulter, Brea, CA) and analyzed with the FlowJo software (FlowJo LLC, Ashland, OR).

Transferrin and RBC uptake assays

For holo-TF uptake assays, PBMC were incubated with Alexa488-labeled holo-TF (10 µg/ml, Thermo Fisher, Waltham, MA). To assess true levels of endocytosed holo-TF, extracellular TF-Al488 signal was quenched by incubation with 0.1% trypan blue (Sigma-Aldrich, Darmstadt, Germany) for 5 minutes.

For generation of heat-stressed RBC, erythrocytes were obtained from the pellet fraction after Biocoll gradient centrifugation (see Cell Isolation), washed twice with PBS and incubated at 42 ºC for 20

minutes. To isolate senescent circulating RBC, whole blood was layered onto a Percoll (Sigma-Aldrich, Darmstadt, Germany) gradient series (from the tube bottom 1.080, 1.072, 1.068, 1.066, 1.064 g/l) and centrifuged at 2000 g at 25 ºC without braking. The densest RBC fraction (1.08+ g/l), described to be enriched in senescent RBC(10), and the pooled 1.068 and 1.066 g/l fractions ('young' RBC) were utilized in uptake experiments. RBC were labeled with PKH26 (Sigma-Aldrich, Darmstadt, Germany) according to the manufacturer's protocol.

For ex vivo erythrophagocytosis assays, PBMC were incubated with PKH26-labeled RBC (10 RBC: 1 PBMC) and analyzed by flow cytometry at the indicated time points(5, 11, 12). To discriminate between monocyte-adhering and phagocytosed RBC co-staining for extracellular CD235a was employed. In some experiments, PBMC: RBC co-cultures were supplemented with control 50 µg/ml rat IgG, anti-CD172a (clone SE5A5), anti-CD47 (CC2C6) or anti-TIM4 (9F4) antibodies (BioLegend, San Diego, CA) to modulate RBC uptake mediated by the respective proteins.

Radioactive ^{59}Fe uptake assay, ^{59}Fe ferritin incorporation assay

In ^{59}Fe uptake experiments, MACS-purified $CD14^+$ monocytes were cultured in 500 µl 10% FCS RPMI medium with 0.5 µM $^{59}Fe^{3+}$ ($FeCl_3$, Perkin Elmer, Waltham, MA), resulting in TBI formation, or with a mixture of 0.5 µM $^{59}Fe^{3+}$ and 10 µM $^{56}Fe^{3+}$ in form of $FeCl_3$ (NTBI) resulting in NTBI formation (Supplementary Figure S4D). After 4 hour incubation at 37 ºC, radioactivity of TBI and NTBI culture supernatant as well as cell-free media was determined with a gamma counter (Wallac, Perkin Elmer, Waltham, MA).

In ferritin incorporation experiments, MACS-purified $CD14^+$ monocytes were cultured in presence of ^{59}Fe-labeled TBI and NTBI as described above. The cultures were additionally stimulated with 2 µg/ml hepcidin or vehicle. After 4 hour incubation at 37 ºC, cells were pelleted by centrifugation, the

supernatant removed and the cell fraction washed twice with PBS. Cell lysates were resolved by electrophoresis as described(13). ^{59}Fe-ferritin bands (approx. 400 kDa) were visualized by autoradiography.

Statistics

Data plotting was accomplished with R (R Foundation for Statistical Computing, Vienna, Austria) and the ggplot2 package. Unless indicated, data were plotted as point – bar plots, where each point represents one measurement, bars denote mean, error bars represent SEM.

Statistical analysis was performed with R with the stat, lme4 and lmerTest packages. For statistical analysis of categorical variable data (study group comparison, comparison of particular experimental time points) first-order mixed-effect linear models of the general form were applied (only fixed terms are shown, example for two factors):

$$response = \beta_0 + \beta_1 variable_1 + \beta_2 variable_2 + \beta_{1:2} variable_1 variable_2 \quad ,$$

where β_o stands for the model intercept estimate, β_n for the estimate of the nth factor and $\beta_{n:m}$ for the estimate of the nth factor and mth factor interaction.

For rate determination and comparison of saturable kinetic processes (calcein quenching, RBC uptake, FPN1 induction, TF conversion rate) data were fitted to a second-order mixed-effect linear model approximating a one-phase association function with the following general formula (only fixed terms are shown):

$$response = \beta_0 + \beta_1 t + \beta_2 t^2 \quad ,$$

where t stands for time, β_1 estimates the rate of the process and β_2 controls the maximal response value.

Random terms in the models included effects of the cell donor (in case when cells from the same donor where subjected to multiple measurements), donor: sample interaction (in case when more populations

from the same sample were compared) and/or donor: time point interaction (in case when multiple samples obtained from the same donor were analyzed in each time point).

Significance of the model estimates (p ($\beta \neq 0$)) was assessed with two-tailed unpaired T tests (degrees of freedom approximated with the Kenward-Roger's algorithm), significance of the model terms was assessed with ANOVA (Kenward-Roger's degrees of freedom) and log-likelihood ratio tests. Estimate p values were corrected for multiple comparisons with the Benjamini-Hochberg method. Model diagnostics (residual normality and goodness of the fit) was accomplished by visual inspection of the distribution of model residuals and of the residual versus fitted plots. Model estimates are presented in data plots with 95% confidence intervals (CI) and p values. p values < 0.05 are considered significant.

Supplementary References

1. Liu B et al. CD14++CD16+ Monocytes Are Enriched by Glucocorticoid Treatment and Are Functionally Attenuated in Driving Effector T Cell Responses. *J. Immunol.* 2015;194(11):5150–60.

2. Wong KL et al. Gene expression profiling reveals the defining features of the classical, intermediate, and nonclassical human monocyte subsets. *Blood* 2011;118(5):e16–e31.

3. Cros J et al. Human CD14dim Monocytes Patrol and Sense Nucleic Acids and Viruses via TLR7 and TLR8 Receptors. *Immunity* 2010;33(3):375–386.

4. Sturn A, Quackenbush J, Trajanoski Z. Genesis: cluster analysis of microarray data. *Bioinformatics* 2002;18(1):207–8.

5. Theurl I et al. On-demand erythrocyte disposal and iron recycling requires transient macrophages in the liver. *Nat. Med.* 2016;22(8):945–951.

6. Ross SL et al. Identification of Antibody and Small Molecule Antagonists of Ferroportin-Hepcidin Interaction. *Front. Pharmacol.* 2017;8:838.

7. Epsztejn S, Kakhlon O, Glickstein H, Breuer W, Cabantchik ZI. Fluorescence analysis of the labile iron pool of mammalian cells. *Anal. Biochem.* 1997;248(1):31–40.

8. Prus E, Fibach E. Flow cytometry measurement of the labile iron pool in human hematopoietic cells. *Cytom. Part A* 2008;73A(1):22–27.

9. Kakhlon O, Cabantchik ZI. The labile iron pool: Characterization, measurement, and participation in cellular processes. *Free Radic. Biol. Med.* 2002;33(8):1037–1046.

10. Bizjak DA, Brinkmann C, Bloch W, Grau M. Increase in Red Blood Cell-Nitric Oxide Synthase Dependent Nitric Oxide Production during Red Blood Cell Aging in Health and Disease: A Study on Age Dependent Changes of Rheologic and Enzymatic Properties in Red Blood Cells. *PLoS One* 2015;10(4):e0125206.

11. de Almeida AC, Barbosa SM, de Lourdes Rios Barjas-Castro M, Olalla-Saad ST, Condino-Neto A. IFN-β, IFN-γ, and TNF-α decrease erythrophagocytosis by human monocytes independent of SIRP-α or SHP-1 expression. *Immunopharmacol. Immunotoxicol.* 2012;34(6):1054–1059.

12. Fendel R et al. New method to quantify erythrophagocytosis by autologous monocytes. *Cytom. Part A* 2007;71A(4):258–264.

13. Kwok JC, Richardson DR. Examination of the mechanism(s) involved in doxorubicin-mediated iron accumulation in ferritin: studies using metabolic inhibitors, protein synthesis inhibitors, and lysosomotropic agents. *Mol. Pharmacol.* 2004;65(1):181–195.

Supplementary Table S1

Characteristics of the microarray studies selected for analysis of differential expression of iron-related genes

	GSE25913	GSE66936	E-MEXP-2544
**Repository**	GEO	GEO	Array Express
**Title**	Gene expression profiling of the classical (CD14+ +CD16-), intermediate (CD14++CD16+) and non-classical (CD14+CD16+) human monocyte subsets	Expression data from intermediate monocytes from healthy donors and autoimmune uveitis patients	Transcription profiling of human monocyte subpopulations
**Study design**	Three human monocyte subsets, the CD14+ +CD16- classical, the CD14++CD16+ intermediate and CD14+CD16+ non-classical subsets were purified using fluorescence activated cell sorting from peripheral blood mononuclear cells. RNA was processed from the three monocyte subsets from 4 individual donors in duplicates, giving a total of 24 samples.	Peripheral blood from 5 autoimmune uveitis patients and 4 healthy donors were collected. Immediately after collection, we purified circulating intermediate CD14++CD16+ monocytes from 5 patients with autoimmune uveitis (Sample 13-17; titled as P intermediate 1-5) and 4 healthy donors (Sample 1, 4, 7, 10; titled as HD intermediate 1-4) by flow cytometry and isolated total RNA to proceed microarray assay. In addition, we also purified CD14+CD16++ (Sample 3, 6, 9, 12; titled HD non-classical 1-4) and CD14++CD16- (Sample 2, 5, 8, 11; titled HD classical 1-4) from 4 healthy donors to do microarray.	CD14hiCD16lo, CD14loCD16hi, and CD14hiCD16hi cells were sorted from the blood of three healthy controls (D1, D2, and D3), the samples from donor D1 are in triplicate.
**Microarray platform**	Illumina human-6 v2.0 expression beadchip	Affymetrix Human Genome U133 Plus 2.0 Array	Agilent Whole Human Genome Microarray 4x44K
**Literature link**	Wong KL, Tai JJ, Wong WC, Han H et al. Gene expression profiling reveals the defining features of the classical, intermediate, and nonclassical human monocyte subsets. Blood 2011 Aug 4;118(5):e16-31.	Liu B, Dhanda A, Hirani S, Williams EL et al. CD14+ +CD16+ Monocytes Are Enriched by Glucocorticoid Treatment and Are Functionally Attenuated in Driving Effector T Cell Responses. J Immunol 2015 Jun 1;194(11):5150-60.	Cros J, Cagnard N, Woollard K et al. Human CD14dim monocytes patrol and sense nucleic acids and viruses via TLR7 and TLR8 receptors. Immunity. 2010 Sep 24;33(3):375-86.
**Remarks**	Technical duplicates averaged (arithmetic mean) for the analysis	Only healthy donor samples analyzed	D1 triplicates averaged (arithmetic mean) for the analysis

Supplementary Table S2

Table of 523 iron-related genes, whose differential expression in monocyte subsets was investigated. The table contains Gene Bank identifiers, HGNC symbols and Benjamini-Hochberg corrected ANOVA p values for the monocyte subpopulation term. Shaded cells indicate p values less than 0.1. The Table can be found online as an Excel file.

Supplementary Table S3

ANOVA statistics for the first-order linear models used to evaluate monocyte subset-specific differences in iron turnover proteins in Figure 3B and Supplementary Figure S3B.

Marker	Figure	Df	F	P
FPN1	Figure 3	2, 18	47	< 0.0001
TFR1		2, 18	45	< 0.0001
Ferritin		2, 6	82	< 0.0001
DMT1		2, 12	2.1	ns
ZIP14		2, 12	6.3	0.013
CD172a		2, 10	9.4	0.0050
TIM4		2, 10	9.2	0.0053
FPN1	Supplementary Figure S3	2, 6	51	0.00017
TFR1		2, 6	10	0.011
Ferritin		2, 6	31	0.00071
DMT1		2, 6	65	< 0.0001
ZIP14		2, 6	24	0.0014
CD172a		2, 6	14	0.0056

Supplementary Table S4

ANOVA statistics for the second-order linear models used to determine rates of calcein quenching in Figures 5A and B.

Model description	Figure	Monocyte subset	Term	Df	F	P
quenching in monocyte subtypes	Figure 5A	NA	rate	1, 27	230	< 0.0001
			rate:subtype	2, 27	87	< 0.0001
quenching in ctrl vs. hepcidin treated cells	Figure 5B	classical	rate	1, 10	9.8	0.011
			rate:hepcidin	1, 13	11	0.0048
		intermediate	rate	1, 10	30	0.00027
			rate:hepcidin	1, 13	9.7	0.0083
		non-classical	rate	1, 10	34	0.00016
			rate:hepcidin	1, 13	0.073	ns

n: m stands for interaction between factor n and factor m

Supplementary Table S5

ANOVA statistics for the second-order linear models used to determine rates of calcein quenching in Figures 7 and 8.

Substance	Figure	Term	Df	F	P
DFO	Figure 7A	rate	1, 21	3.1	ns (0.092)
		rate:stimulation	1, 22	12	0.0022
Ascorbate	Figure 7B	rate	1, 21	4.3	0.05
		rate:stimulation	1, 22	12	0.0021
BPS	Figure 7C	rate	1, 21	4.2	ns (0.052)
		rate:stimulation	1, 22	3.2	ns (0.089)
Bafilomycin	Figure 8A	rate	1, 21	5.3	0.032
		rate:stimulation	1, 22	7.8	0.011
ZnCl2	Figure 8B	rate	1, 21	6.4	0.02
		rate:stimulation	1, 22	7	0.015
Bicarbonate	Figure 8C	rate	1, 21	4.5	0.045
		rate:stimulation	1, 22	8.4	0.0084
Citrate	Figure 8D	rate	1, 21	5.3	0.031
		rate:stimulation	1, 14	12	0.0032

rate: stimulation stands for interaction between rate and stimulation with the given substance

Supplementary Table S6

ANOVA statistics for the first-order linear models used to determine differences in FPN1 expression, and calcein fluorescence between study populations (Figures 9 and 10).

Compared study groups	Response	Figure	Monocyte subset	Df	F	P
healthy vs. HH1	FPN1	Figure 9A	classical	2, 17	3.3	ns (0.061)
			intermediate	2, 15	2.7	ns (0.098)
			non-classical	2, 19	13	<0.0001
healthy vs. iron overload	FPN1	Figure 9A	classical	1, 15	7	0.018
			intermediate	1, 14	2.8	ns
			non-classical	1, 18	5.5	0.031
healthy vs. HH1	calcein	Figure 9B	classical	2, 7.9	1.6	ns
			intermediate	2, 7.5	2.9	ns
			non-classical	2, 8	1.4	ns
healthy vs. iron overload	calcein	Figure 9B	classical	1, 8.7	3.5	ns (0.094)
			intermediate	1, 8.4	6.6	0.032
			non-classical	1, 8.8	3.1	ns
healthy vs MDS FT strata	FPN1	Figure 10A	classical	1, 20	3	ns (0.098)
			intermediate	1, 11	0.061	ns
			non-classical	1, 27	0.5	ns
	calcein	Figure 10B	classical	1, 14	7.1	0.019
			intermediate	1, 11	3.4	ns (0.092)
			non-classical	1, 14	1.3	ns

Supplementary Table S7

ANOVA statistics for the second-order linear models used to determine differences RBC uptake rates between monocyte subsets and differences in uptake rates for young and senescent RBC (Figures 11 and 12).

Model description	Figure	Monocyte subset	Term	Df	F	P
RBC uptake rate in monocyte subtypes	Figure 11	NA	rate	1, 20	52	< 0.0001
			rate:subtype	2, 40	86	< 0.0001
Uptake of young vs senescent RBC	Figure 12	classical	rate	1, 30	38	< 0.0001
			rate:RBC age	1, 30	8.5	0.0066
		intermediate	rate	1, 10	35	0.00015
			rate:RBC age	1, 15	15	0.0014
		non-classical	rate	1, 30	46	< 0.0001
			rate:RBC age	1, 30	11	0.0026

n: m stands for interaction between factor n and factor m

Supplementary Table S8

ANOVA statistics for the first-order linear models used to determine differences in FPN1 expression and intracellular CD235a levels between control and transfused individuals (Figure 13).

Compared study groups	Response	Figure	Monocyte subset	Df	F	P
healthy vs transfusion	FPN1	Figure 13A	classical	1, 27	7.5	0.011
			intermediate	1, 27	0.13	ns
			non-classical	1, 27	1	ns
	CD235a	Figure 13B	classical	1, 27	5.4	0.028
			intermediate	1, 27	4.6	0.042
			non-classical	1, 27	1.9	ns

Supplementary Table S9

Demographic characteristics and blood parameters of healthy individuals and genetic iron overload study participants

	healthy				HH1				p (healthy vs HH1)	statistical test	FPN1 mut
	Value/Mean	*SD*	*Min*	*Max*	*Value/Mean*	*SD*	*Min*	*Max*			*Value/Mean*
**Sex (F/M)**	6/12	NA	NA	NA	1/7	NA	NA	NA	ns	Chi sqared	0/1
**Age**	33	8	24	46	48	14	29	72	0.016	Two-tailed T test	60
**Hb (g/L)**	150	13	130	171	158	10	144	174	ns	Two-tailed T test	161
**Hct**	42.7	3.4	37.7	47.8	44.7	3.0	40.0	48.9	ns	Two-tailed T test	47.0
**MCV (fL)**	85	5	69	91	94	3	87	99	<0.0001	Two-tailed T test	90
**RBC (10^6/µl)**	5.0	0.4	4.3	5.5	4.8	0.3	4.4	5.3	ns	Two-tailed T test	5.2
**WBC (10^3/µl)**	6.5	1.4	5.0	10.3	7.2	1.0	6.0	9.1	ns	Two-tailed T test	5.4
**Ferritin (µg/L)**	100	75	8	312	243	281	32	901	ns	Mann-Whitney test	2409
**TF (mg/dL)**	278	42	228	381	223	40	175	308	0.0064	Two-tailed T test	212
**TF Sat (%)**	30	10	11	50	44	25	11	87	ns	Two-tailed T test	19
**CRP (mg/L)**	0.07	0.02	0.06	0.11	0.2	0.2	0.1	0.7	0.026	Mann-Whitney test	0.2
**Creatinin (mg/dL)**	0.93	0.15	0.66	1.25	0.9	0.2	0.7	1.3	ns	Two-tailed T test	1.1

Hb – hemoglobin, Hct – hematocrit, MCV – mean corpuscular volume, RBC – red blood cell count, WBC – white blood cell count, TF – transferrin, TF Sat – TF saturation, CRP – C-reactive protein

Supplementary Table S10

Demographic characteristics and blood parameters of healthy individuals and MDS subject stratified according to serum ferritin levels (MDS FT^{lo}: serum ferritin < 400 ng/ml, MDS FT^{hi}: serum ferritin > 400 ng/ml)

	healthy				MDS Ftlo				p (healthy vs MDS Ftlo)	MDS Fthi				p (healthy vs MDS Fthi)	statistical test
	Value/Mean	*SD*	*Min*	*Max*	*Value/Mean*	*SD*	*Min*	*Max*		*Value/Mean*	*SD*	*Min*	*Max*		
**Sex (F/M)**	6\12	NA	NA	NA	3\4	NA	NA	NA	ns	1\4	NA	NA	NA	ns	Chi sqared
**Age**	33	8	24	46	75	9	63	87	<0.0001	65	9	55	75	0.00051	Two-tailed T test
**Hb (g/L)**	150	13	130	171	107	31	75	143	0.0094	101	25	68	124	0.0095	Two-tailed T test
**Hct**	42.73	3.35	37.70	47.80	41.01	24.09	22.10	92.00	ns	29.60	7.74	19.00	37.70	0.017	Two-tailed T test
**MCV (fL)**	85	5	69	91	82	26	28	109	ns	96	13	84	115	ns	Two-tailed T test
**RBC (10^6/µl)**	5.0	0.4	4.3	5.5	3.5	1.2	2.0	5.2	0.018	3.1	0.6	2.3	3.7	0.00074	Two-tailed T test
**WBC (10^3/µl)**	6.5	1.4	5.0	10.3	6.7	9.2	1.4	27.4	ns	4.2	3.0	2.2	9.3	ns	Two-tailed T test
**Ferritin (µg/L)**	100	75	8	312	215	144	29	391	ns	1001	509	552	1704	0.00091	Mann-Whitney test
**TF (mg/dL)**	278	42	228	381	221	97	79	374	ns	202	54	134	257	0.029	Two-tailed T test
**TF Sat (%)**	30	10	11	50	41	20	21	72	ns	57	29	24	92	ns	Two-tailed T test
**CRP (mg/L)**	0.07	0.02	0.06	0.11	2.7	6.1	0.1	16.5	0.0032	1.6	2.9	0.1	6.7	0.022	Mann-Whitney test
**Creatinin (mg/dL)**	0.93	0.15	0.66	1.25	1.3	0.5	0.7	2.1	ns	1.1	0.3	0.8	1.6	ns	Two-tailed T test
**IPSSR**	NA	NA	NA	NA	2.3	1.7	0.5	5.5	NA	5.3	2.0	3.5	7	0.029	Mann-Whitney test *

* MDS FT^{lo} and MDS FT^{hi} groups compared

Hb – hemoglobin, Hct – hematocrit, MCV – mean corpuscular volume, RBC – red blood cell count, WBC – white blood cell count, TF – transferrin, TF Sat – TF saturation, CRP – C-reactive protein, IPSSR – Revised International Scoring System

Supplementary Table S11

Demographic characteristics and blood parameters of healthy individuals and transfusion-receiving patients

	healthy				transfusion				p (healthy vs transfusion)	statistical test
	Value/Mean	*SD*	*Min*	*Max*	*Value/Mean*	*SD*	*Min*	*Max*		
**Sex (F/M)**	7/9				5/10				ns	Chi sqared
**Age**	35	8	26	53	63	14	44	86	p<0.0001	Two-tailed T test
**Hb (g/L)**	145	12	123	165	89	11	73	107	p<0.0001	Two-tailed T test
**Hct**	0.41	0.03	0.35	0.45	0.27	0.03	0.21	0.31	p<0.0001	Two-tailed T test
**MCV (fL)**	86	5	78	98	84	7	70	96	ns	Two-tailed T test
**RBC (10^6/µl)**	4.9	0.4	4.2	5.5	3.2	0.5	2.4	4.4	p<0.0001	Two-tailed T test
**WBC (10^3/µl)**	6.2	0.9	5.0	7.6	5.3	3.6	0.8	12.4	ns	Two-tailed T test
**Ferritin (µg/L)**	88	54	11	202	1029	1941	13	7482	0.013	Mann-Whitney test
**TF (mg/dL)**	270	43	224	390	221	85	69	356	ns (0.067)	Two-tailed T test
**TF Sat (%)**	34	15	17	71	28	19	7	72	ns	Two-tailed T test
**CRP (mg/L)**	0.12	0.14	0.06	0.58	7.6	8.2	0.1	25.4	p<0.0001	Mann-Whitney test
**Creatinin (mg/dL)**	0.93	0.15	0.66	1.25	1.3	0.5	0.7	2.4	0.018	Two-tailed T test

Hb – hemoglobin, Hct – hematocrit, MCV – mean corpuscular volume, RBC – red blood cell count, WBC – white blood cell count, TF – transferrin, TF Sat – TF saturation, CRP – C-reactive protein

Supplementary Table S12

Antibodies used in flow cytometry experiments.

Antigen	Clone or Order No.	Dilution	Provider	Secondary staining
CD3	OKT3	1:200	Biolegend (San Diego, CA) or Thermo Fisher (Waltham, MA)	none
CD14	M5E2	1:200 – 1:400		
CD16	3G8	1:200		
CD15	HI98	1:400		
CD19	HIB19	1:200		
CD56	CMSSB	1:200		
TfR1/CD71	CY1G4	1:200		
CD172a	15-414	1:200		
CD235a	HIR2	1:200		
TIM4	9F4	1:200		
HLA-DR	TU36	1:200		
DMT1	ab55735	1:200	Abcam (Cambridge, United Kingdom)	Goat anti-mouse Alexa488 (Poly4053, Biolegend, San Diego, CA)
ZIP14	ab106568	1:200	Abcam (Cambridge, United Kingdom)	Donkey anti-rabbit DyLight488 (Poly4064, Biolegend, San Diego, CA)
Ferritin	F5012	1:400	Sigma-Aldrich (Darmstadt, Germany)	
FPN1*	38G6	1:100	Amgen (Thousand Oaks, CA)	none

* labeling with the APEX Pacific Blue Antibody labeling kit (Thermo Fisher)

Supplementary Figure S1

Scheme of the study selection process and re-analysis of publicly available microarray data. Identification of genes with common expression across the monocyte subsets.

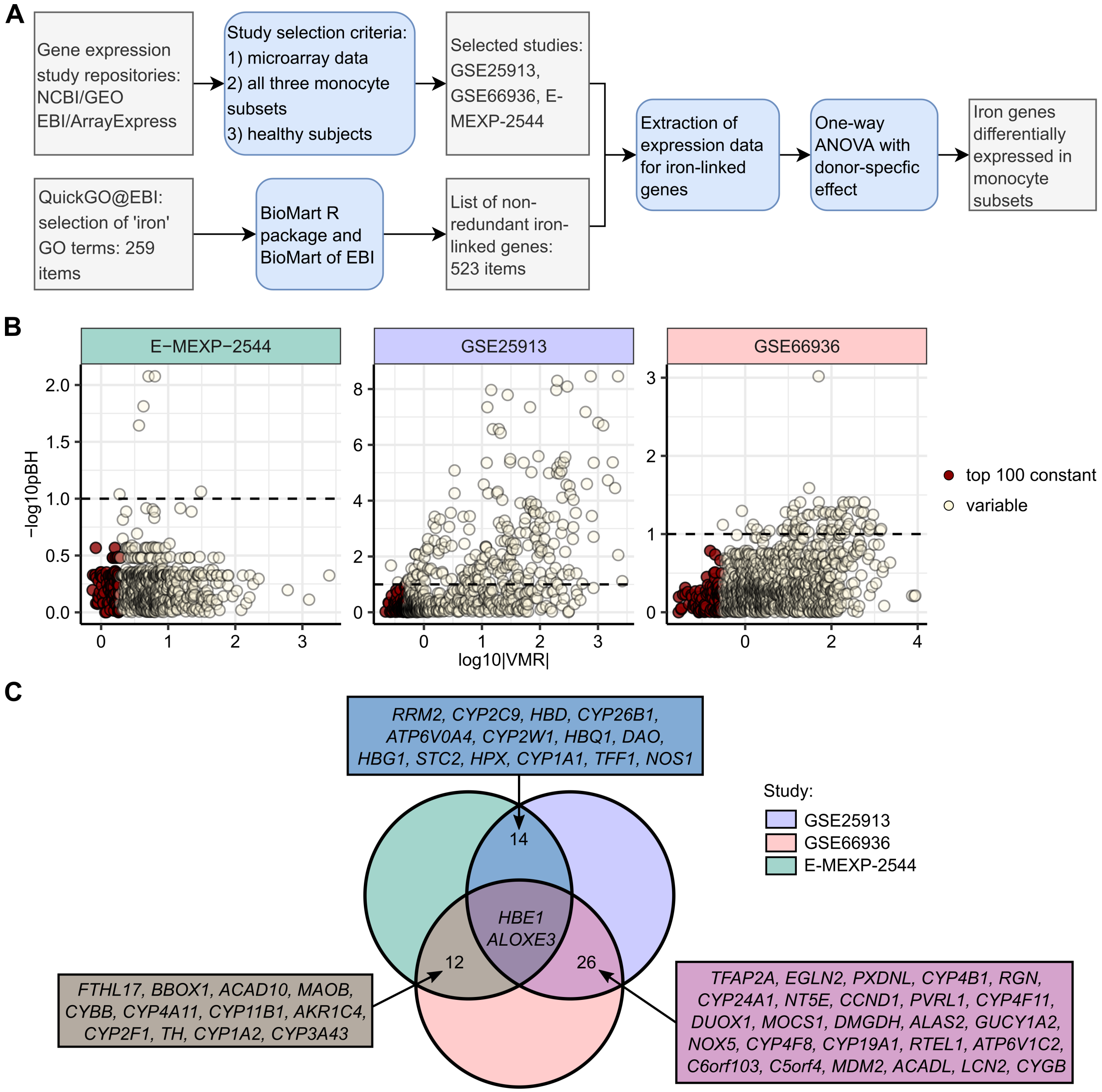

(A) GEO and Array Express repositories were screened for studies investigating monocyte gene expression. The selection criteria were: availability of data for all three monocyte subsets, data obtained with healthy individuals and usage of microarray technique. Next, a list of iron-related gene ontology terms (GO) was extracted from the EBI QuickGo server. The GO list was then converted to a non-redundant gene ID list using the BioMart of EBI. The corresponding expression values were extracted

from the selected study data (expression values normalized by the authors) and screened for the significantly differentially expressed genes with one-way ANOVA with a donor effect adjustment.
(B) Identification of top 100 genes with the least expression variance between monocyte subsets in each study. Subset-specific expression level variance (MSS, mean sum of squares) was extracted from the ANOVA table (A) and divided by the mean expression for each iron-related gene giving the Mean-Variance Ratio (MVR). 100 genes with the lowest absolute values of MVR and Benjamini-Hochberg-corrected p-values > 0.1 (A) for each study were selected. Graphs visualize $-\log_{10}$ Benjamini-Hochberg-corrected p-values plotted against $\log_{10}$ absolute values of MVR; each point denotes a single iron-linked gene, the top 100 least variable genes are colored with red. Horizontal dashed lines indicate $p = 0.1$ for each study.
(C) Genes with the least expression variance between monocyte subsets identified in at least two studies with the method presented in (B) are presented in a Venn diagram.

Supplementary Figure S2

Gating strategy used to define monocyte subsets by flow cytometry.

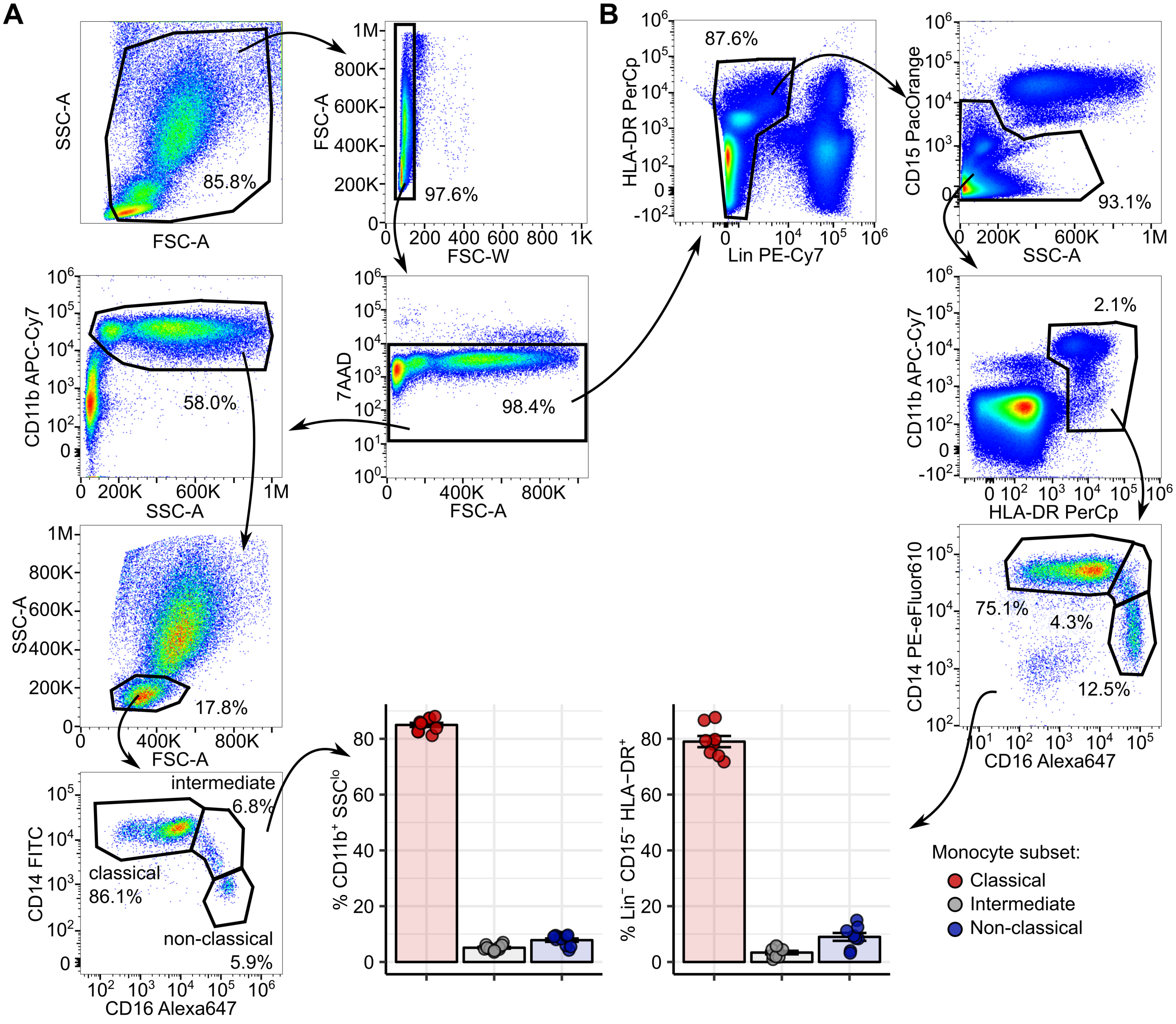

(A) Gating strategy used in Figures 3, 4, 5, 7, 8, 11 and 12. Classical monocytes are defined as $CD11b^{+}$ SSC^{lo} $CD14^{+}$ $CD16^{-/lo}$, intermediate as $CD11b^{+}$ SSC^{lo} $CD14^{+}$ $CD16^{+}$, non-classical as $CD11b^{+}$ SSC^{lo} $CD14^{-}$ $CD16^{+}$ cells. The graph displays percentages (n = 9 healthy individuals) of classical (red), intermediate (gray) and non-classical monocytes (blue) within the $7AAD^{-}$ $CD11b^{+}$ SSC^{lo} subset: each point represents one measurement, bars denote mean, error bars represent SEM.

(B) Gating strategy used in Supplementary Figure S3. Lineage stands for CD3, CD19 and CD56 staining. Classical monocytes are defined as $Lineage^{-}$ $CD15^{-}$ $SSC\text{-}A^{lo}$ $HLA\text{-}DR^{+}$ $CD14^{+}$ $CD16^{-/lo}$, intermediate as $Lineage^{-}$ $CD15^{-}$ $SSC\text{-}A^{lo}$ $HLA\text{-}DR^{+}$ $CD14^{+}$ $CD16^{+}$, non-classical as $Lineage^{-}$ $CD15^{-}$ $SSC\text{-}A^{lo}$ $HLA\text{-}DR^{+}$ $CD14^{-}$ $CD16^{+}$ cells. The graph shows percentages (n = 8 healthy individuals) of classical, intermediate and non-classical monocytes within the $Lineage^{-}$ $CD15^{-}$ $SSC\text{-}A^{lo}$ $HLA\text{-}DR^{+}$ subset: each point represents one measurement, bars denote mean, error bars represent SEM.

Supplementary Figure S3

Expression of iron metabolism proteins in blood monocytes defined by HLA-DR expression.

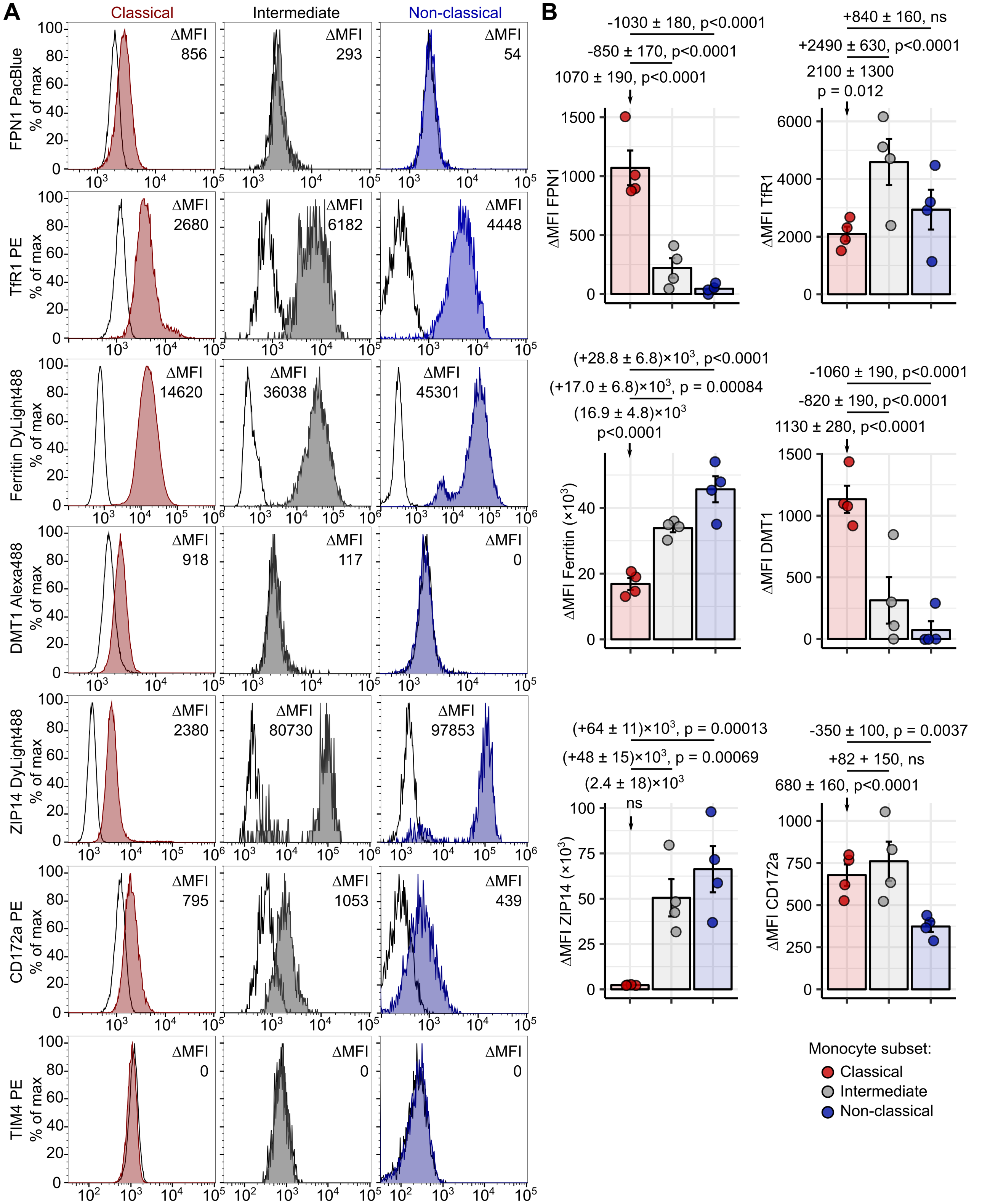

Blood monocyte populations were defined as presented in Supplementary Figure S2B. Whole blood samples and PBMC were obtained from healthy individuals (n = 4). Expression of surface FPN1, TfR1, DMT1, ZIP14, CD172a, TIM4 and intracellular ferritin in monocyte subsets (classical monocytes: red, intermediate: gray, non-classical: blue) was measured by flow cytometry.
(A) Representative signal histograms (open histograms: isotype, tinted histograms: specific antibody). For TIM4 no specific signal on cell surface could be detected in any of the monocyte subsets.
(B) Graphs with ΔMFI values; each point represents one measurement, bars denote mean, error bars represent SEM. Statistical significance was assessed with first-order linear modeling. Estimates for protein expression in classical monocytes and for differences in expression between subsets as presented with 95% CI. Estimate p values were calculated with two-tailed T test. ANOVA statistics are shown in Supplementary Table S3.

Supplementary Figure S4

Purification of classical and intermediate monocytes with MACS. Monitoring apo-TF to holo-TF conversion by absorbance measurements. NTBI content of Fe^{3+} spiked monocyte culture medium.

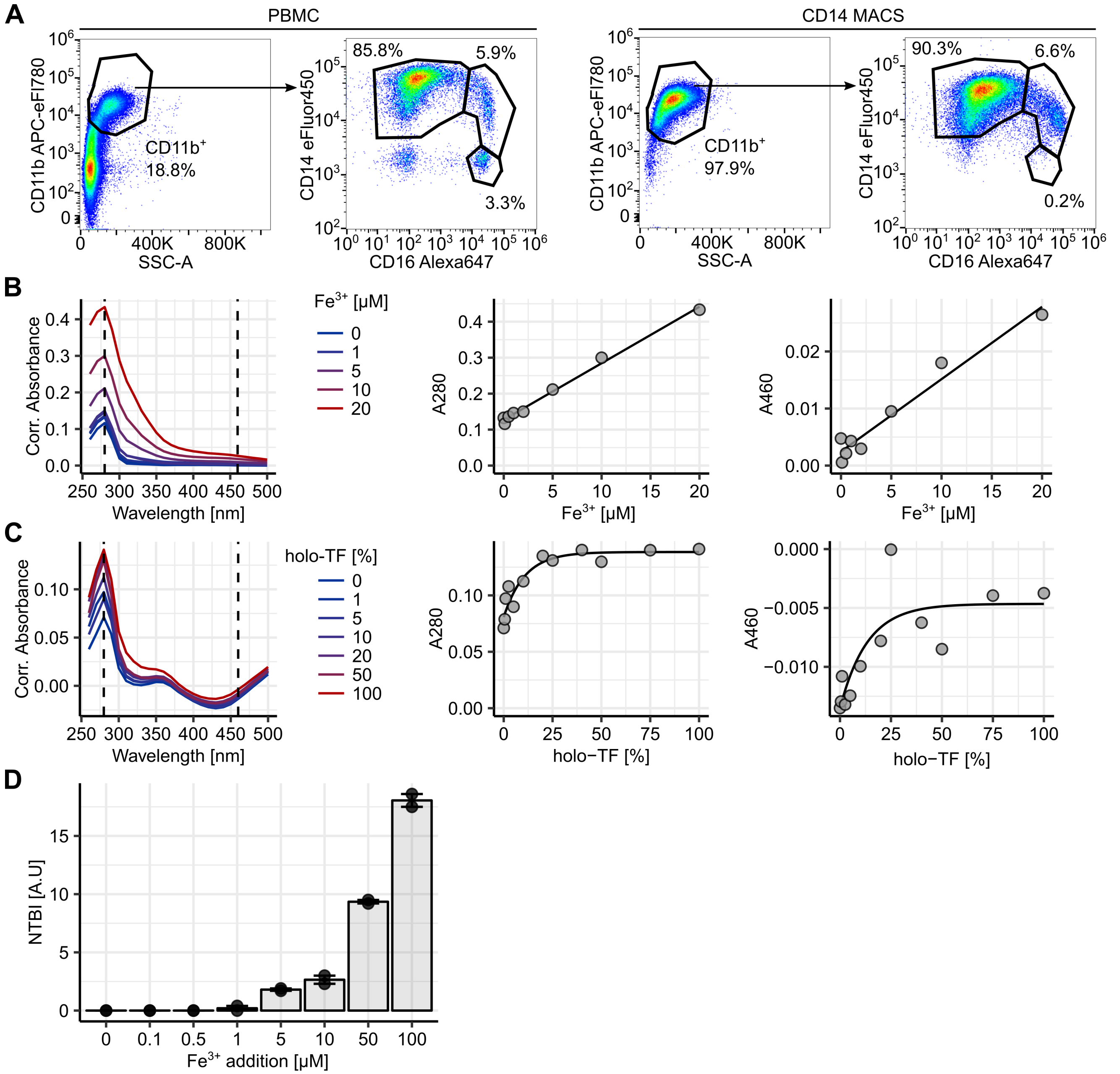

(A) $CD14^{+}$ cells were isolated by MACS from PBMC (n = 3 healthy volunteers). Monocyte population composition in the source PBMCs and $CD14^{+}$ fraction was investigated by flow cytometry. Representative results for one cell donor out of three examined are shown. Monocyte subtypes were identified as presented in Supplementary Figure S2A.

(B) Absorbance of solutions containing the indicated percentages of holo-TF in RPMI medium supplemented with 1% FCS and apo-TF (0.5 mg/ml total TF) was measured in the range of 250 – 500 nm. 1% FCS RPMI medium served as a blank sample. Representative absorbance spectra are shown.

Graphs display mean absorbance values at 280 and 460 nm as points (each points is a mean of two technical replicates) with one-phase association trends (solid line). Representative data from one out of three experiments performed are shown.
(C) 0.5 mg/ml apo-TF solution in 1% FCS RPMI medium was supplemented with the indicated concentrations of Fe^{3+} ($Fe_2(SO_4)_3$) and incubated for 10 minutes at room temperature. Absorbance was measured in the 250 – 500 nm range. 1% FCS RPMI medium served as a blank sample. Representative absorbance spectra are shown. Graphs display absorbance values at 280 and 460 nm as points (each points is a mean of two technical replicates) with linear trends (solid line). Representative data from one out of three experiments performed are shown.
(D) 10% FCS-containing RPMI medium was supplemented with the indicated Fe3+ concentrations (Fe2(SO4)3). NTBI was measured after 10 min incubation at room temperature as described in Methods. Representative data from one out of three experiments performed are shown (2 technical replicates within one experiment); each point represents one measurement, bars denote mean, error bars represent SEM.

Supplementary Figure S5

Kinetics of labile iron accumulation in human leukocyte populations.

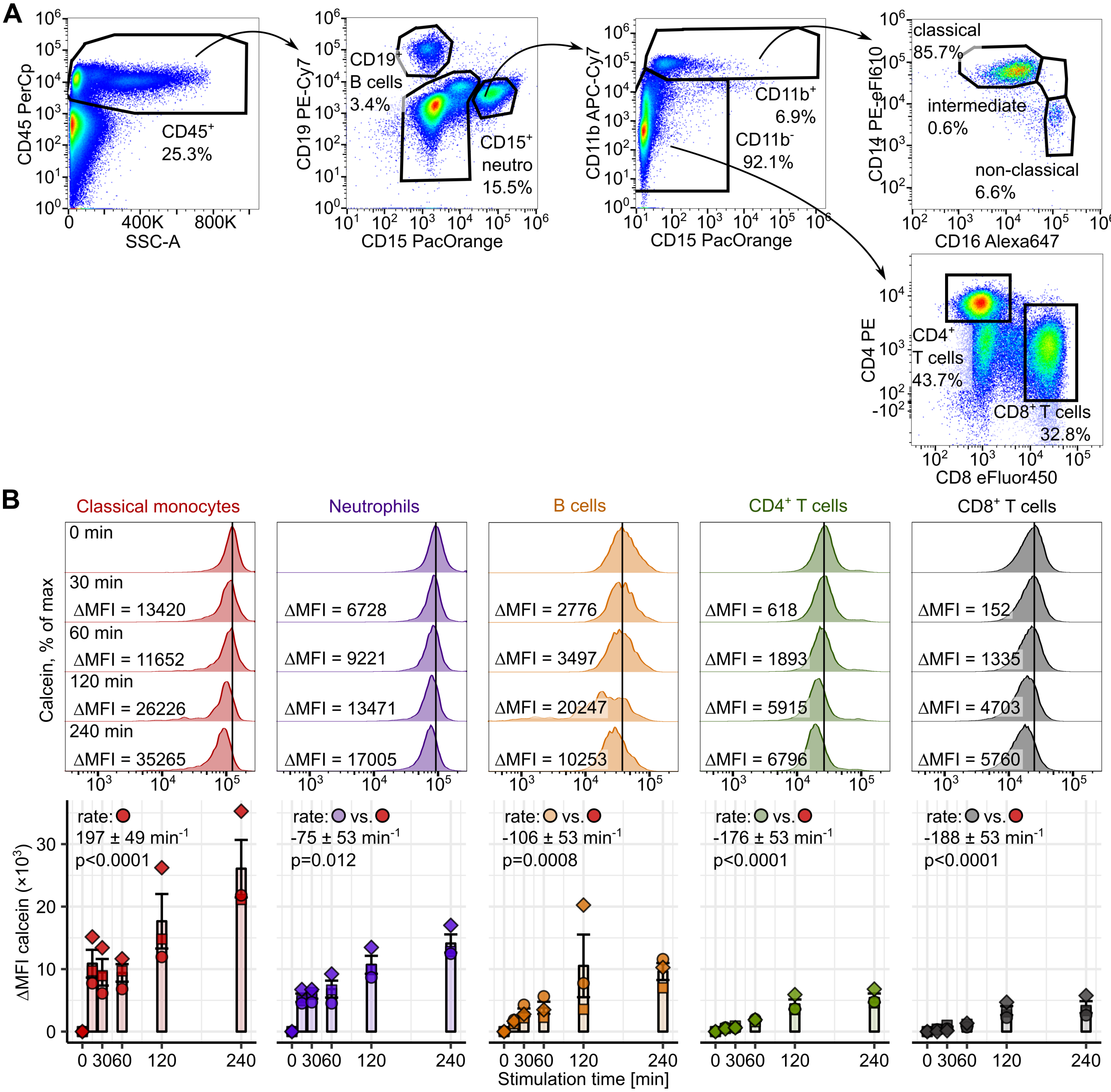

Leukocytes were isolated from RBC-lysed whole blood (n = 4 healthy donors), labeled with calcein and incubated with 10 µM Fe^{3+} ($Fe_2(SO_4)_3$) for the indicated time points.

(A) Gating strategy for identification of blood leukocyte populations. Viable single cells were identified as presented in Supplementary Figure S2. B cells were defined as $CD45^+$ $CD19^+$, neutrophils as $CD45^+$ $CD15^+$, classical monocytes as $CD45^+$ $CD19^-$ $CD15^-$ $CD11b^+$ $CD14^+$ $CD16^{-/lo}$, $CD4^+$ T lymphocytes as $CD45^+$ $CD19^-$ $CD15^-$ $CD4^+$, $CD8^-$ T lymphocytes as $CD45^+$ $CD19^-$ $CD15^-$ $CD4^-$ $CD8^+$ cells.

(B) Calcein fluorescence in blood leukocytes (classical monocytes: red, neutrophils: violet, B cells: orange, CD4+ T cells: green, CD8+ cells: gray) was measured by flow cytometry. Blood leukocyte

populations were identified as presented in Supplementary Figure S5A. Representative calcein histograms are shown. Graphs depicts calcein ΔMFI values in leukocyte populations: each point represents one measurement, bars denote mean, error bars represent SEM. Symbol shape codes for the cell donor. Rate of calcein quenching in classical monocytes and differences in quenching rate between a particular leukocyte type and classical monocytes were determined with a second-order linear model. Model estimates are shown with 95% CI. Estimate p values were calculated with two-tailed T test. ANOVA for the rate and rate: leukocyte type interaction terms: $p_{rate} = 0.0005$ ($F_{1, 13} = 21$), $p_{rate: leukocyte type} < 0.0001$ ($F_{4, 64} = 15$).

Supplementary Figure S6

Uptake of transferrin-bound iron by human monocytes.

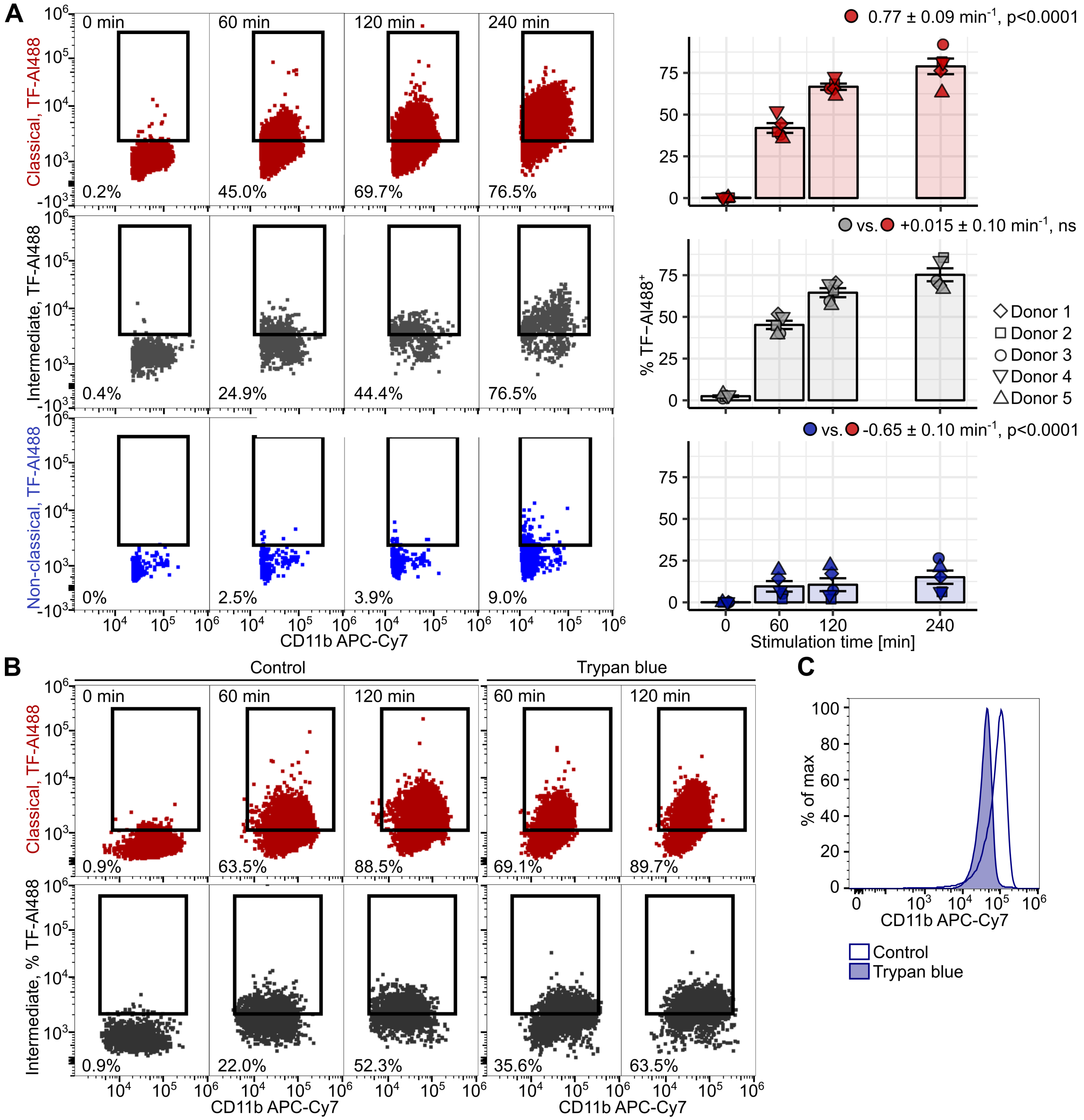

(A) PBMC (n = 5 healthy donors) were incubated for the indicated time points with 10 µg/ml Alexa488-labeled holo-transferrin (TF-Al488) or medium alone. Monocyte subtypes were identified as presented in Supplementary Figure S2A. Alexa488-positivity in monocyte subpopulations (classical monocytes: red, intermediate: gray, non-classical: blue) was measured by flow cytometry. Representative cytometry plots are presented. Graphs depict percentages of TF-Al488-positive cells in each monocyte subset: each point represents one measurement, bars denote mean, error bars represent

SEM. Symbol shape codes for the cell donor. Rate of change in percent of Alexa488$^+$ cells in classical monocytes and differences in rate between particular monocyte subsets were determined with a second-order linear model. Model estimates are shown with 95% CI. Estimate p values were calculated with two-tailed T test. ANOVA for the rate and rate: monocyte subset interaction terms - $p_{rate} < 0.0001$ ($F_{1, 13} = 270$), $p_{rate: leukocyte type} < 0.0001$ ($F_{2, 36} = 93$).

(B) To differentiate between cell surface-bound TF-Al488 and internalized TF-Al488, extracellular fluorescence was quenched by incubation with trypan blue. Note no reduction of the signal intensity indicating intracellular localization of the TF-Al488 in classical monocytes. Representative results for one cell donor out of five examined are shown.

(C) Signal reduction for CD11b APC-Cy7 after trypan blue incubation served as positive control for quenching efficiency. Representative results for one cell donor out of five examined are shown (open histogram: control cells, blue tinted histogram: after trypan blue treatment).

Supplementary Figure S7

LIP accumulation and FPN1 regulation in classical monocytes upon TBI and NTBI stimulation. Preferential uptake of NTBI by classical monocytes as compared to TBI.

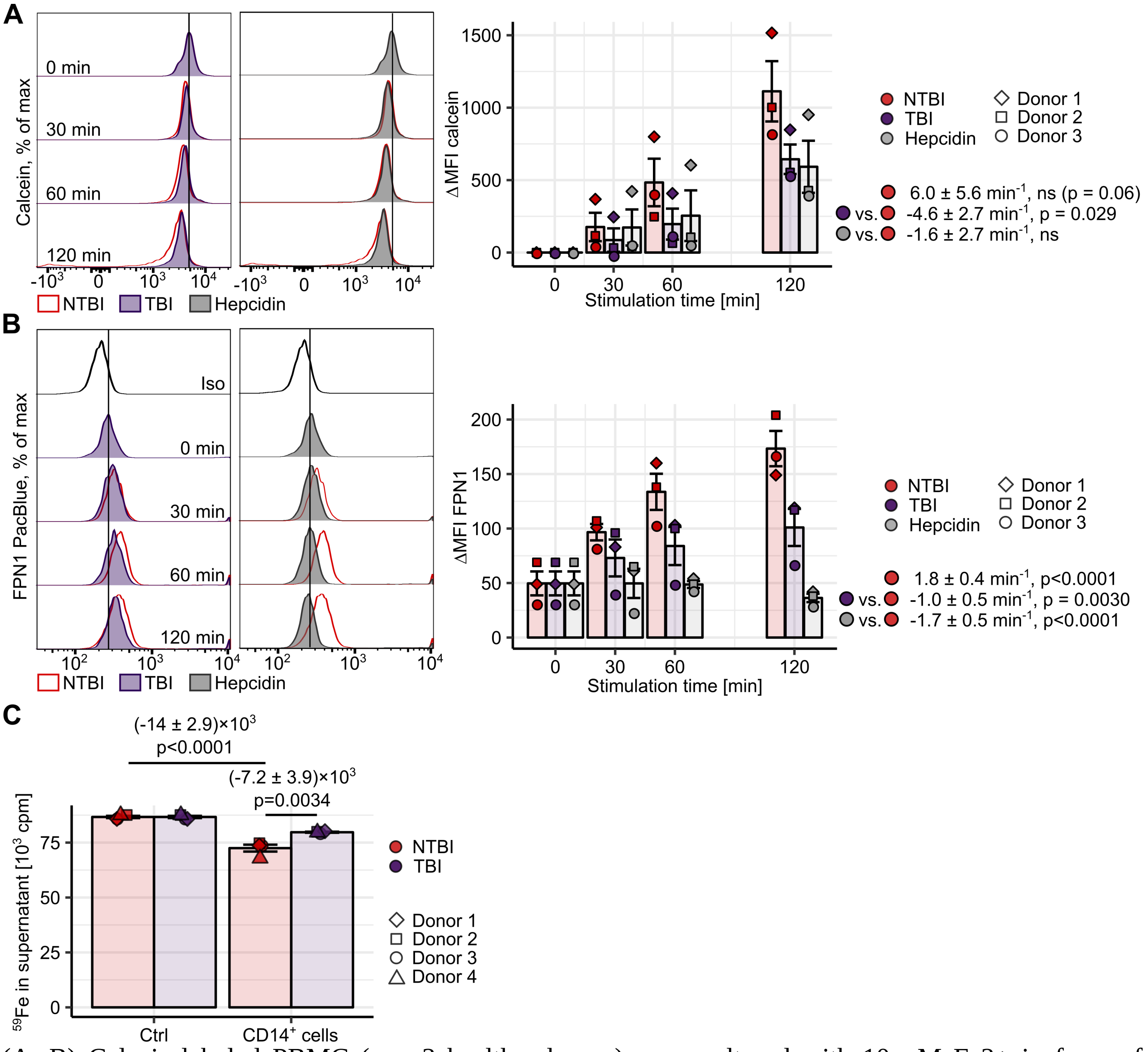

(A, B) Calcein-labeled PBMC (n = 3 healthy donors) were cultured with 10 µM Fe3+ in form of Fe2(SO4)3 (NTBI), holo-TF (TBI) or with 2 µg/ml hepcidin for the indicated time points. Classical monocytes were identified as presented in Supplementary Figure S2A. Calcein fluorescence and surface FPN1 expression in classical monocytes was measured by flow cytometry. Representative signal histograms are shown (red open histograms: NTBI, tinted violet histograms: TBI, tinted gray histograms: hepcidin). Graph show ΔMFI values: each point represents one measurement, bars denote mean, error bars represent SEM (NTBI: red, TBI: violet, hepcidin: gray). Symbol shape codes for the cell donor. Rates of calcein quenching and of FPN1 induction were determined with a second-order linear model. Estimates are shown with 95% CI. Estimate p values were calculated with two-tailed T test. (A) Estimates for calcein quenching rate in NTBI-stimulated monocytes and for differences in

quenching rate between cells stimulated with NTBI and particular substance. ANOVA for the rate and rate: stimulation interaction model terms: p_{rate} = ns ($F_{1, 7}$ = 2.0), $p_{rate: stimulation}$ = 0.018 ($F_{2, 20}$ = 5.0). (B) Estimates for FPN1 induction rate in NTBI-stimulated monocytes and for differences in induction rate between cells stimulated with NTBI and particular substance. ANOVA for the rate and rate: stimulation interaction model terms: p_{rate} = 0.013 ($F_{1, 7}$ = 27), $p_{rate: stimulation}$ < 0.0001 ($F_{2, 20}$ = 19).
(C) MACS-purified CD14+ monocytes (n = 4 healthy donors) were cultured with 0.5 µM 59Fe3+ (TBI) or 0.5 µM 59Fe3+ together with 10 µM 56Fe3+ (NTBI) for 4 hours. Iron content in supernatant was determined by 59Fe radioactivity measurement (counts per minute, CPM) for the TBI- and NTBI-containing media alone (Ctrl) and monocyte culture supernatant (CD14+ cells). Graph depicts levels of Fe in supernatant: each point represents one measurement, bars denote mean, error bars represent SEM (NTBI: red, TBI: violet). Symbol shape codes for the cell donor. Statistical significance for differences in culture conditions were determined with a first-order linear model. Model estimates are presented with 95% CI. Estimate p values were calculated with two-tailed T test. ANOVA statistics for the $CD14^+$ cells and $CD14^+$ cells: iron form interaction terms: p_{CD14+} < 0.0001 ($F_{1, 11}$ = 146), $p_{CD14+:iron\ form}$ = 0.0017 ($F_{1, 11}$ = 17).

Supplementary Figure S8

Levels of intracellular ferritin in monocyte subsets upon short-term NTBI and hepcidin stimulation.

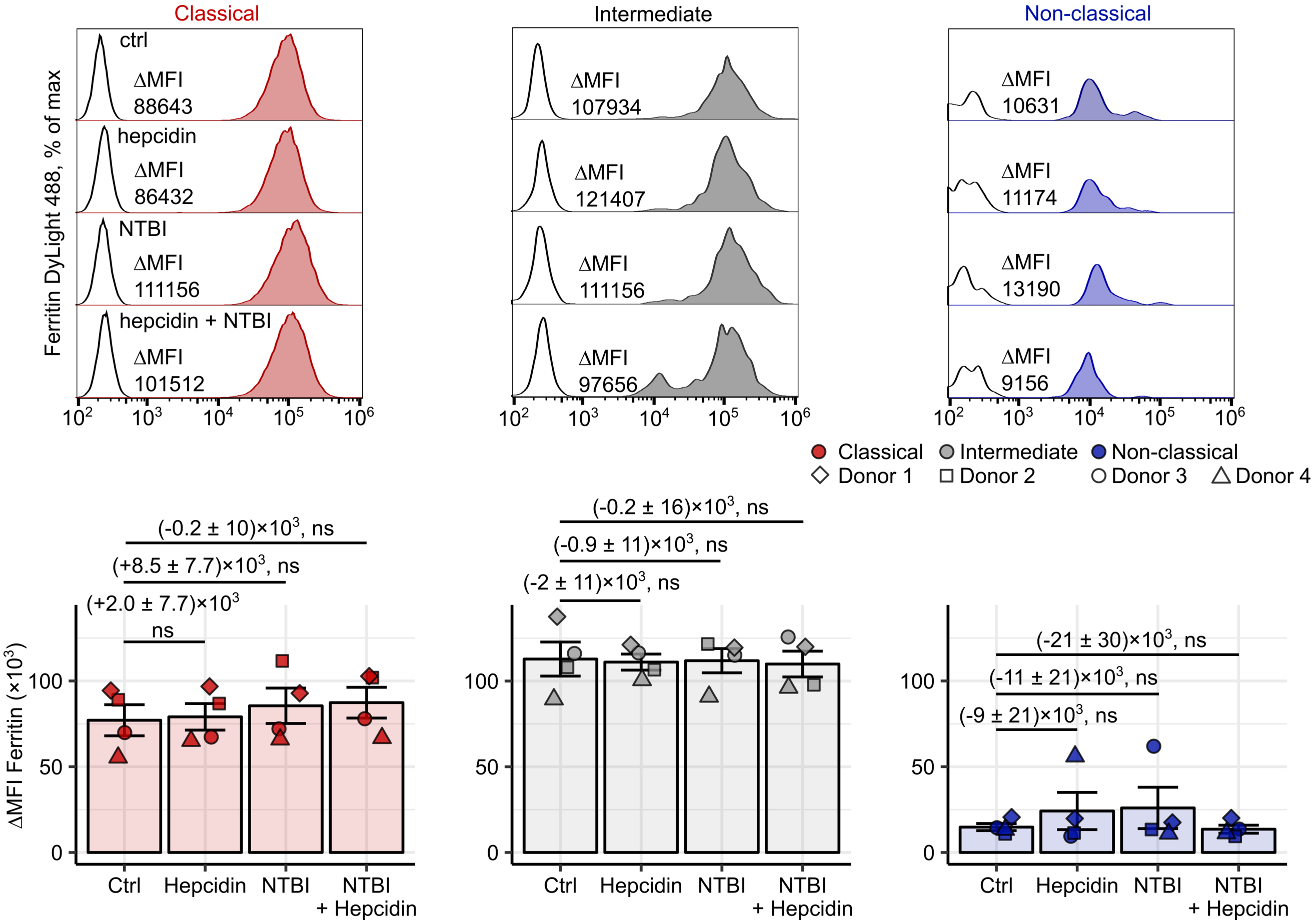

PBMCs (n = 4 healthy donors) were cultured for 4 hours in 10% FCS RPMI medium supplemented with 10 µM Fe3+ and/or 2 µg/ml hepcidin. Monocyte subtypes were identified as presented in Supplementary Figure S2A. Intracellular ferritin in monocyte subpopulations (classical monocytes: red, intermediate: gray, non-classical: blue) was measured by flow cytometry. Representative ferritin signal histograms are shown (open histograms: isotype, tinted histograms: ferritin). Graphs display ΔMFI values: each point represents one measurement, bars denote mean, error bars represent SEM (NTBI: red, TBI: violet). Symbol shape codes for the cell donor. Statistical significance for the differences between control and stimulated cells were determined with first-order linear models. A separate model was applied to each monocyte subset. Model estimates are shown with 95% CI. Estimate p values were calculated with two-tailed T test. ANOVA for the model terms: classical monocytes - p_{iron} = 0.020 ($F_{1, 9}$ = 8.0), $p_{hepcidin}$ = ns ($F_{1, 9}$ = 0.4), $p_{iron: hepcidin}$ = ns ($F_{1, 9}$ = 0.010); intermediate monocytes - p_{iron} = 0.020 ($F_{1, 9}$ = 0.62), $p_{hepcidin}$ = ns ($F_{1, 9}$ = 0.20), $p_{iron: hepcidin}$ = ns ($F_{1, 9}$ = 0.0005); non-classical monocytes - p_{iron} = ns ($F_{1, 9}$ = 0.013), $p_{hepcidin}$ = ns ($F_{1, 9}$ = 0.32), $p_{iron: hepcidin}$ = ns ($F_{1, 9}$ = 1.7).

Supplementary Figure S9

Pathways of cellular iron uptake and their modulators. Minimal effects of zinc alone on calcein fluorescence in classical monocytes.

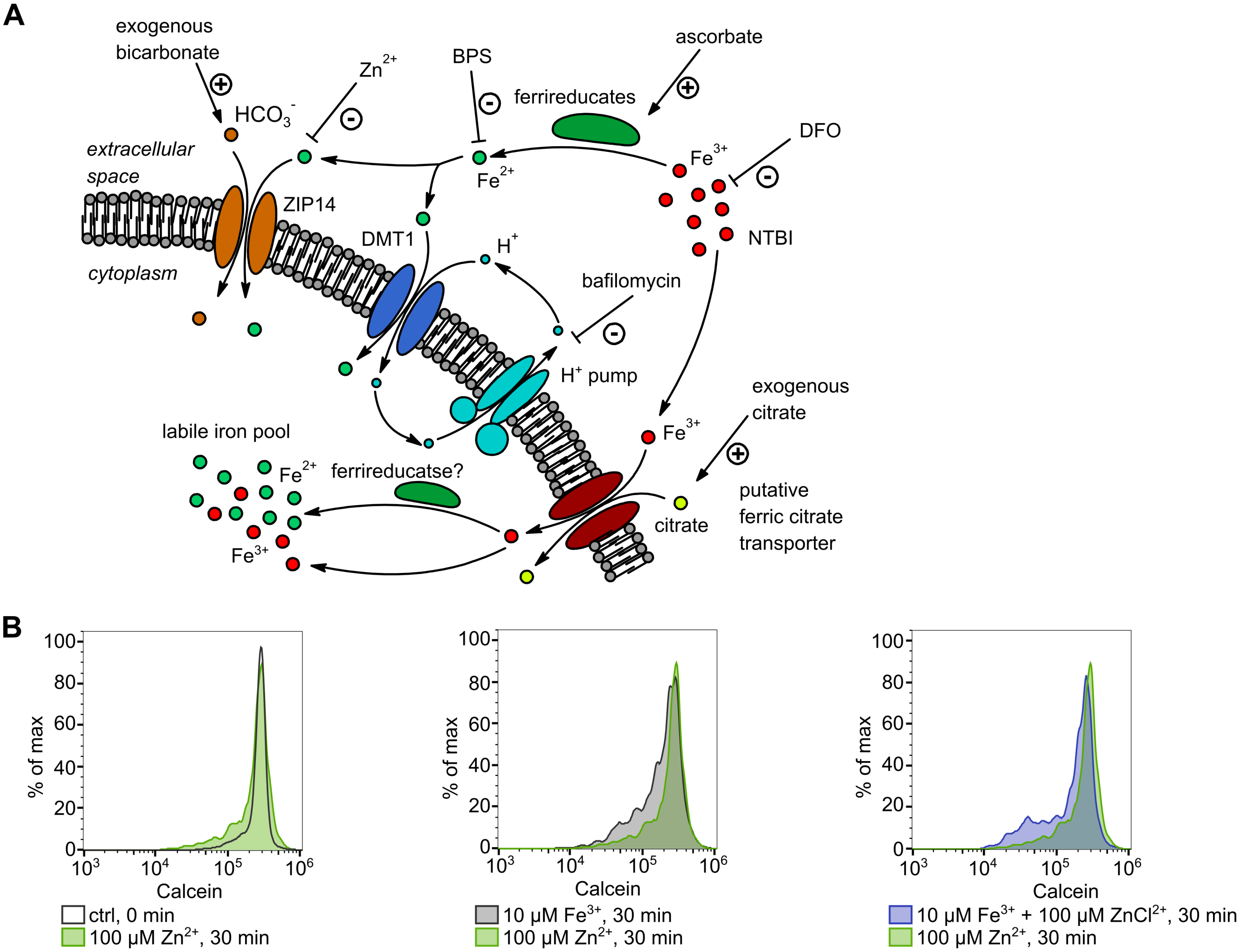

(A) Scheme of cellular Iron uptake pathways with their exogenous activators and inhibitors.
(B) Calcein-labeled PBMC (n = 2 healthy donors) were stimulated with 100 µM $ZnCl_2$, 10 µM Fe^{3+} ($Fe_2(SO_4)_3$) or with the combination thereof for 30 minutes. Calcein fluorescence in classical monocytes was measured by flow cytometry. Classical monocytes were identified as presented in Supplementary Figure S2A. Representative calcein histogram overlays are shown (one cell donor out of two examined); note far less calcein fluorescence quenching in the zinc alone sample as compared to the Fe^{3+}/Zn^{2+}-stimulated classical monocytes.

Supplementary Figure S10

Supplementary Figure S10. Gating strategies for identification of monocyte and leukocyte populations in the patient study comparing healthy, iron overload and MDS individuals.

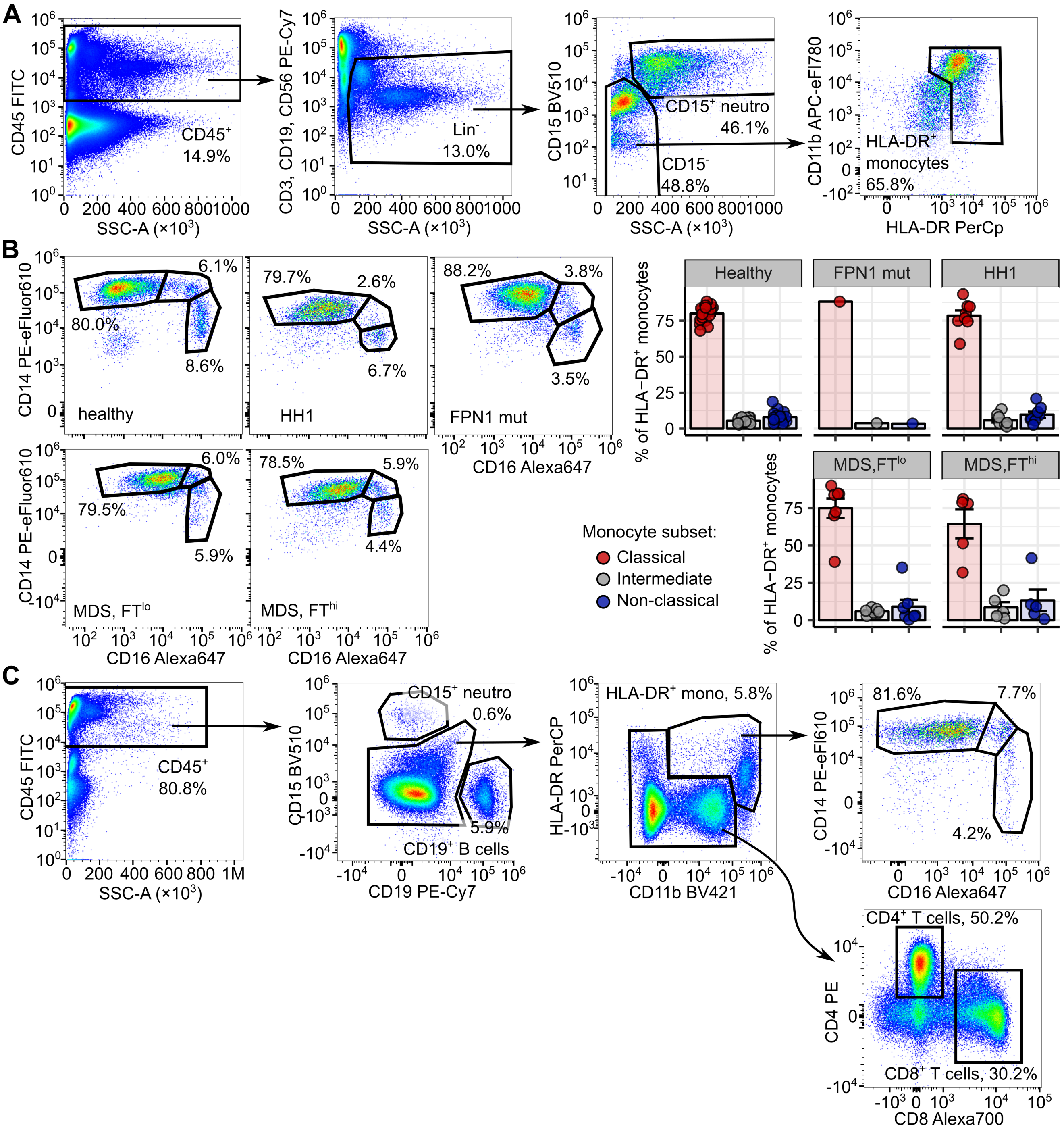

Viable single cells were identified as presented in Supplementary Figure S2. Lineage staining encompasses CD3, CD19 and CD56.

(A) Gating strategy for identification of monocyte subsets and determination of calcein fluorescence (in PBMC) and FPN1 expression (in whole-blood monocytes) in Figures 9 and 10. Pan-monocytes were defined as Lineage^{-} CD15^{-} HLA-DR^{+} cells. Classical subset was defined as CD14^{+} CD16$^{-/lo}$, intermediate as CD14^{+} CD16^{+}, non-classical as CD14^{-} CD16^{+} monocytes.
(B) Monocyte subset distribution pattern in healthy (n = 18), HH1 (n = 6), FPN1 mutant (n = 1), MDS FTlo (serum ferritin < 400 ng/ml, n = 7) and MDS FThi (serum ferritin > 400 ng/ml, n = 5) study participants as defined with the strategy presented in (A). Representative cytometry results are shown (gating for Lineage- CD15- HLA-DR+ cells). Graphs display percentages of monocyte subsets within Lineage- CD15- HLA-DR+: each point represents one measurement, bars denote mean, error bars represent SEM (classical monocytes: red, intermediate: gray, non-classical: blue).
(C) Gating strategy for measurement of calcein fluorescence in PBMC leukocyte populations (Supplementary Figure 11C). Neutrophils were defined as CD45^{+} CD15^{+} CD19^{-} cells, B cells as CD45^{+} CD15^{-} CD19^{+}, classical monocytes as CD45^{+} CD15^{-} CD19^{-} HLA-DR^{+} CD14^{+} CD16$^{-/lo}$, intermediate monocytes as CD45^{+} CD15^{-} CD19^{-} HLA-DR^{+} CD14^{+} CD16^{+}, non-classical monocytes as CD45^{+} CD15^{-} CD19^{-} HLA-DR^{+} CD14^{+} CD16^{-}, CD4^{+} T cells as CD45^{+} CD15^{-} CD19^{-} HLA-DR^{-} CD4^{+} CD8^{-}, CD8^{+} T cells as CD45^{+} CD15^{-} CD19^{-} HLA-DR^{-} CD4^{-} CD8^{+}.

Supplementary Figure S11

Systemic iron homeostasis parameters in healthy participants, genetic iron overload and MDS subjects. Leukocyte LIP levels in healthy and genetic iron overload participants.

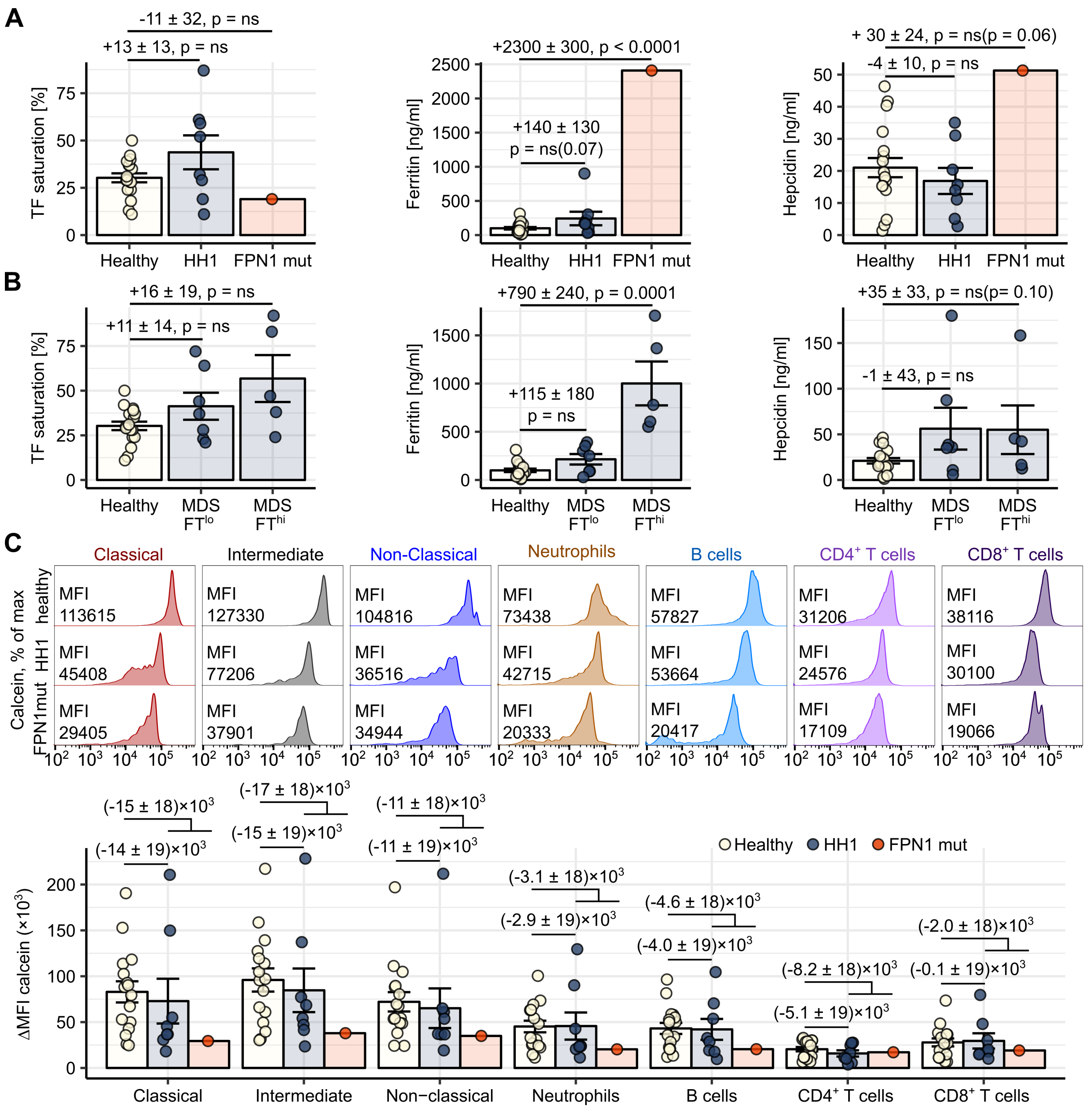

Blood samples were obtained from healthy controls (n = 18), type 1 hemochromatosis patients (HH1, n = 8), a FPN1 loss-of-function heterozygous individual (n = 1) and myelodysplastic syndrome patients with or without hyperferritinemia (MDS FT^{lo}: serum ferritin < 400 ng/ml, n = 7 and MDS FT^{lo}: serum ferritin >400 ng/ml, n = 5).

(A, B) TF saturation, ferritin and serum hepcidin concentration: each point represents one measurement, bars denote mean, error bars represent SEM. Statistical significance for differences between healthy controls and a particular patient group were determined with first-order linear models. Model estimates are shown with 95% CI. Estimate p values were calculated with two-tailed T test. (A) ANOVA statistics: TF saturation - p = ns ($F_{2, 24}$ = 2.4), ferritin - p < 0.0001 ($F_{2, 24}$ = 92), hepcidin - p = 0.048 ($F_{2, 24}$ = 3.4). (B) ANOVA statistics: TF saturation - p = ns ($F_{2, 27}$ = 2.5), ferritin - p < 0.0001 ($F_{2, 27}$ = 39), hepcidin - p = ns ($F_{2, 27}$ = 0.0027).
(C) PBMC from healthy, HH1 and FPN1 mutant study participant were stained with calcein for LIP determination. Blood leukocyte populations were defined as presented in Supplementary Figure S10C. Calcein fluorescence in particular leukocyte types was determined by flow cytometry. Calcein MFI is assumed to be inversely proportional to LIP. Representative calcein fluorescence histograms are presented. Graphs depict calcein MFI values in the leukocyte types: each point represents one measurement, bars denote mean, error bars represent SEM. Statistical significance was assessed with first-order linear models: a model determining LIP differences in a particular leukocyte type between healthy and HH1 subjects and a model determining LIP differences in a particular leukocyte type between healthy and iron overload participants (HH1 and FPN1 mutant). Estimates for the study group: leukocyte type terms are shown with 95% CI, neither of the estimates reached statistical significance calculated with two-tailed T test. ANOVA statistics for the group: leukocyte type terms: healthy vs. HH1 – p = ns ($F_{7, 80}$ = 0.56); healthy vs. overload – p = ns ($F_{7, 86}$ = 0.84).

Supplementary Figure S12

Specificity control for the in vitro erythrophagocytosis assay.

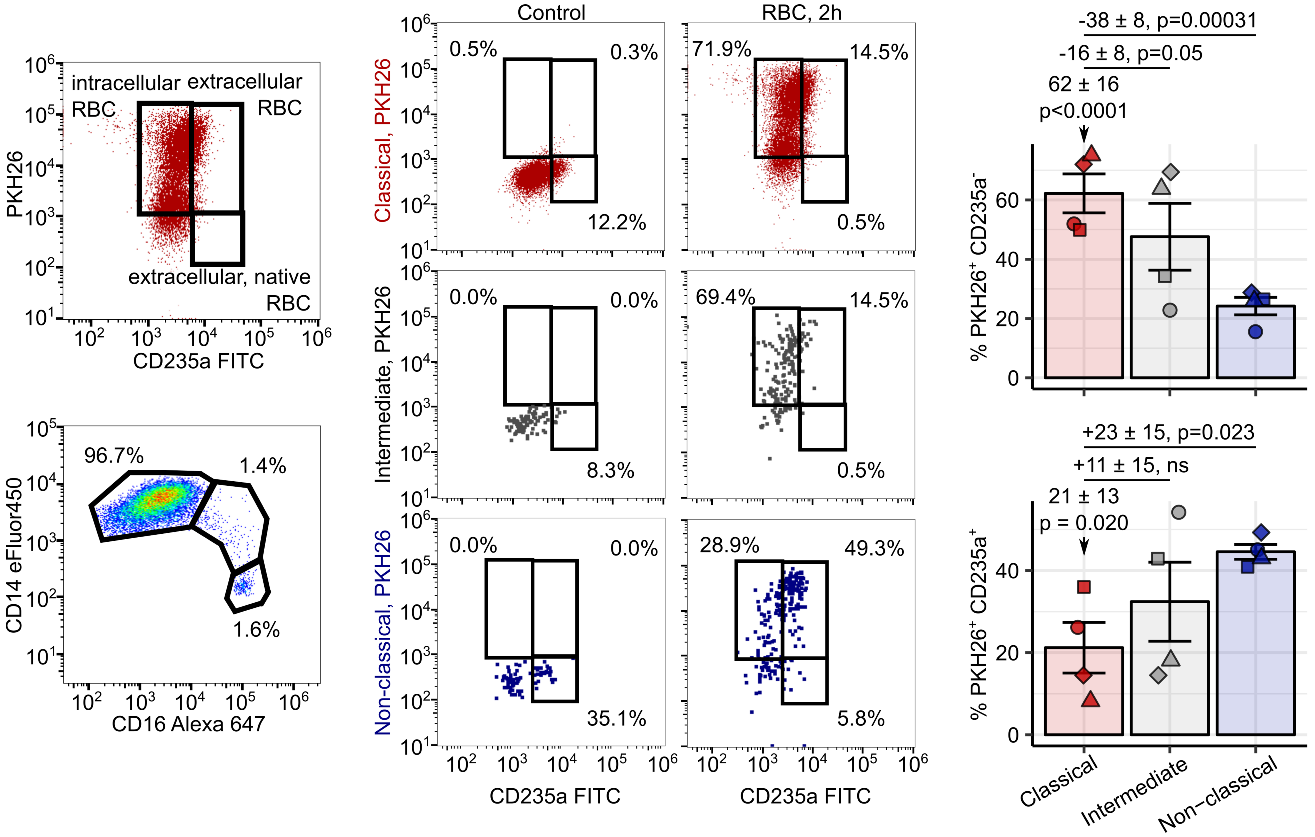

PBMCs (n = 5 healthy donors) were incubated with autologous PKH26-labeled heat-stressed erythrocytes (10 RBC: 1 PBMC) for 2 hours. Monocyte subtypes (classical: red, intermediate: gray, non-classical: blue) were identified as presented in Supplementary Figure S2A. Percentages of monocytes positive for extracellular erythrocyte marker CD235a and PKH26 were measured by flow cytometry. The gates for the CD235a-positive populations were set in respect to the isotype control. Monocytes, which associate with erythrocytes but do not phagocytose them are labeled with the anti-CD235a antibody and could be hence defined as $CD235a^+$ $PKH26^+$ monocyte-erythrocyte multiplets. Monocytes containing phagocytosed erythrocytes are negative for extracellular CD235a and hence defined as $CD235a^-$ $PKH26^+$ cells. Monocytes associating with endogenous, unlabeled erythrocytes are positive for extracellular CD235a but negative for PKH26. Representative cytometry data are shown.

Graphs show percentages of PKH26+ CD235- and PKH26+ CD235+ cells within each monocyte subsets: each point represents one measurement, bars denote mean, error bars represent SEM. Symbol shape codes for the cell donor. Statistical significance was determined with first-order linear models. Estimates are shown with 95% CI. Estimate p values were calculated with two-tailed T test. ANOVA statistics for the percentage of $PKH26^+$ $CD235^-$ cells - p = 0.0043 ($F_{2, 6}$ = 15), for the percentage of $PKH26^+$ $CD235^+$ cells - p = ns (0.074) ($F_{2, 6}$ = 4.1).

Supplementary Figure S13

Effects of CD172a, TIM4 and CD47 blocking on uptake of damaged and physiologically senescent RBC in classical and intermediate monocytes.

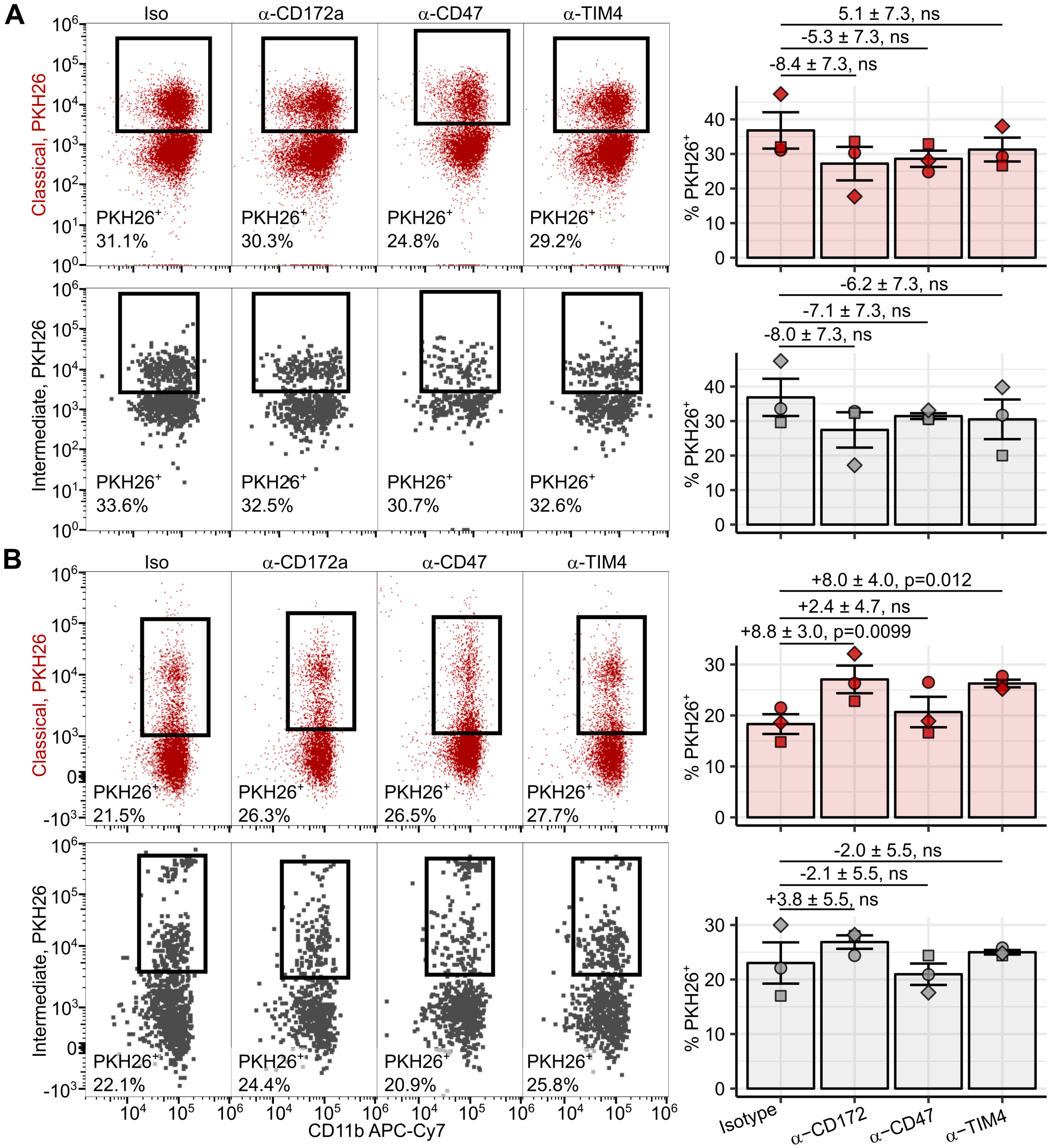

(A, B) PBMCs (n = 3 healthy donors) were incubated with autologous PKH26-labeled heat-stressed (A) or senescent RBC (B) in a ratio of 10 RBC: 1 PBMC for 2 hours. Cultures were supplemented with 50 µg/ml anti-CD172, anti-CD47, anti-TIM4 antibodies or control rat IgG. Monocyte subtypes were identified as presented in Supplementary Figure S2A. PKH26-positivity in classical and intermediate monocytes was estimated by flow cytometry (classical monocytes: red, intermediate: gray). Representative cytometry plots are shown. Graphs display percentages of PKH26+ cells within the

particular monocyte subset: each point represents one measurement, bars denote mean, error bars represent SEM. Symbol shape codes for the cell donor. Statistical significance for differences between the control IgG and a particular blocking antibody sample was determined with first-order linear models. A separate model was applied to each monocyte subset. Estimates are shown with 95% CI. Estimate p values were calculated with two-tailed T test. (A) ANOVA statistics: classical monocytes – p = 0.021 ($F_{3, 18}$ = 4.2), intermediate monocytes - p = ns ($F_{3, 18}$ = 1.7). (B) ANOVA statistics: classical monocytes – p = 0.039 ($F_{3, 6}$ = 5.3), intermediate monocytes - p = ns ($F_{3, 6}$ = 1.3).

Supplementary Figure S14

Supplementary Figure S14. Gating strategy used for the intracellular CD235a staining (Figure 12). Extracellular CD235a expression in monocyte subsets.

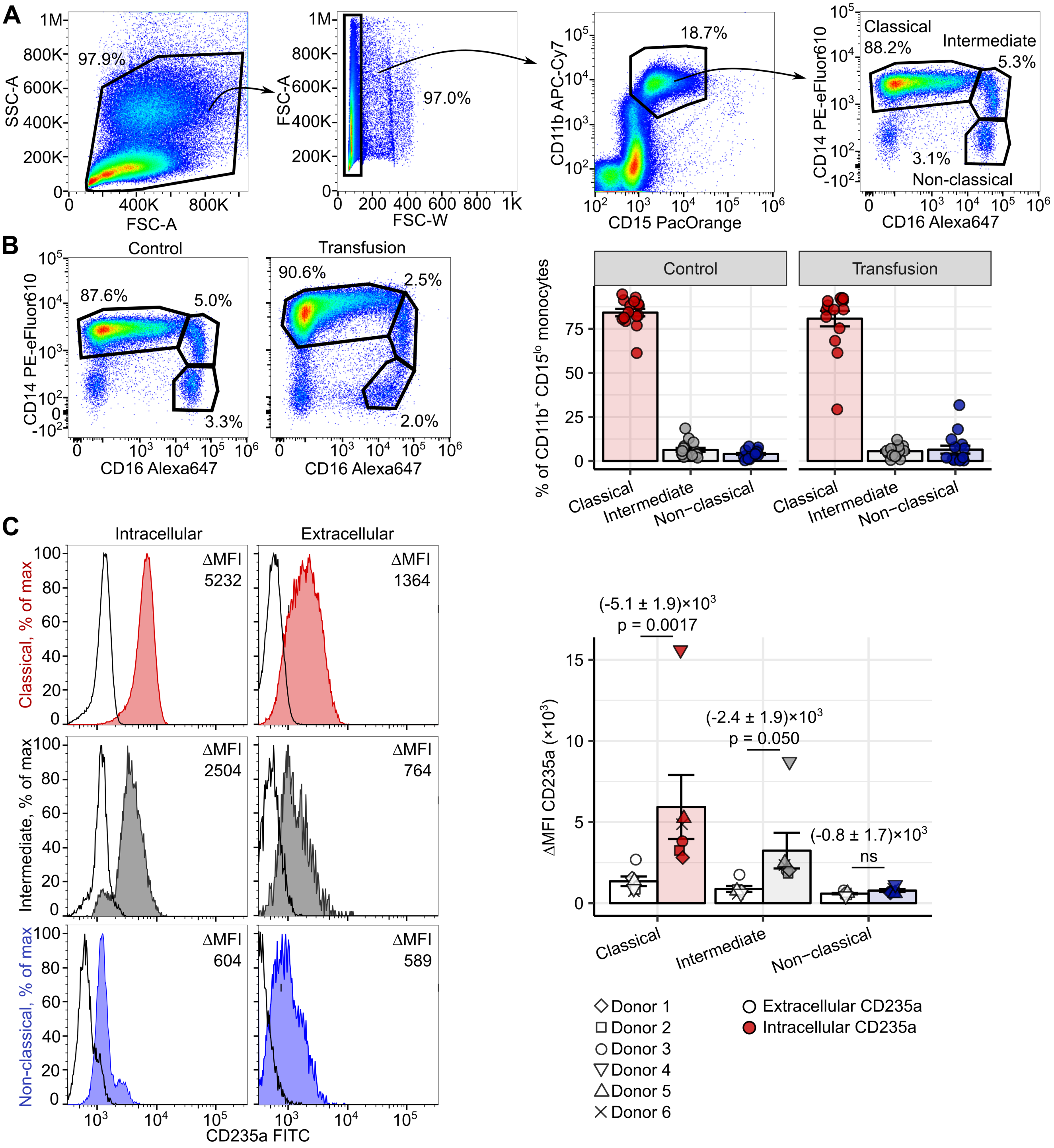

(A) Gating strategy used to define monocyte subpopulations for the intracellular CD235a staining. PBMC were co-labeled with the anti-CD15 antibody to exclude residual $CD15^+$ granulocytes in PBMC preparations.

(B) Monocyte subtype distribution in healthy (n = 16) and transfusion individuals (n = 15). Monocytes subpopulations were identified as presented in Supplementary Figure S14A. Representative cytometry results (gated for $CD15^{lo}$ $CD11b^{+}$ monocytes) are shown. Graphs show percentages of classical, intermediate and non-classical monocytes (classical monocytes: red, intermediate: gray, non-classical: blue) within the $CD15^{lo}$ $CD11b^{+}$ monocytes: **e**ach point represents one measurement, bars denote mean, error bars represent SEM.
(C) Comparison of the intracellular and extracellular CD235a signals in human monocyte subsets. PBMCs (n = 6 healthy donors) were stained for surface monocyte markers and surface CD235a or intracellular CD235a. Samples were measured with the same cytometer settings and intra- and extracellular CD235a signals presented on the same scale. Monocyte subtypes were identified as presented in Supplementary Figure S14A. As seen in the representative signal histograms (open histograms: isotype control, tinted histograms: CD235a) only a minority of CD235a can be detected on the surface of the monocyte. Graph depicts CD235a ΔMFI values for the intra- and extracellular staining in each monocyte subset: each point represents one measurement, bars denote mean, error bars represent SEM (open symbols: extracellular staining, colored symbols: intracellular staining). Symbol shape codes for the cell donor. Statistical significance for differences between intra- and extracellular CD235a staining in each monocyte subset was determined with a first-order linear model. Model estimates are shown with 95% CI. Estimate p values were calculated with two-tailed T test. ANOVA statistics for the CD235a localization: monocyte subset interaction term - p = 0.00010 ($F_{5, 25}$ = 5.9).
